# An Easy Access to Furan-Fused Polyheterocyclic Systems

**DOI:** 10.3390/molecules27103147

**Published:** 2022-05-14

**Authors:** Alice Benzi, Lara Bianchi, Gianluca Giorgi, Massimo Maccagno, Giovanni Petrillo, Domenico Spinelli, Cinzia Tavani

**Affiliations:** 1 Dipartimento di Chimica e Chimica Industriale, Università degli Studi di Genova, Via Dodecaneso 31, 16146 Genova, Italy; alice.benzi93@gmail.com (A.B.); lara.bianchi@unige.it (L.B.); massimo.maccagno@unige.it (M.M.); giovanni.petrillo@unige.it (G.P.); 2 Dipartimento di Biotecnologie, Chimica e Farmacia, Università di Siena, Via A. Moro, 53100 Siena, Italy; gianluca.giorgi@unisi.it; 3 Dipartimento di Chimica “G. Ciamician”, Alma Mater Studiorum-University of Bologna, Via F. Selmi 2, 40126 Bologna, Italy; domenico.spinelli@unibo.it

**Keywords:** nitrostilbenes, naphthofurans, cyclization, electrocyclization

## Abstract

Nitrostilbenes characterized by two different or differently substituted aryl moieties can be obtained from the initial ring-opening of 3-nitrobenzo[*b*]thiophene with amines. Such versatile building blocks couple the well-recognized double electrophilic reactivity of the nitrovinyl moiety (addition to the double bond, followed by, e.g., intramolecular replacement of the nitro group) with the possibility to exploit a conjugated system of double bonds within an electrocyclization process. Herein, nitrostilbenes are reacted with different aromatic enols provided by a double (carbon and oxygen) nucleophilicity, leading to novel, interesting naphthodihydrofurans. From these, as a viable application, aromatization and electrocyclization lead in turn to valuable polycondensed, fully aromatic *O*-heterocycles.

## 1. Introduction

**Figure 1:**
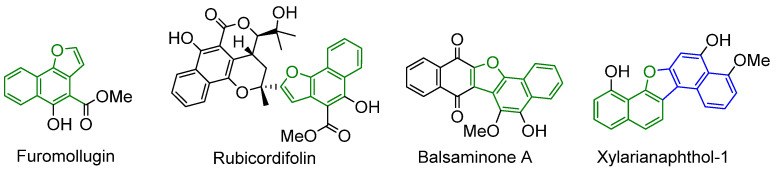
Representative examples of active natural compounds characterized by fused furans.

Benzo- and naphtho-fused furans and dihydrofurans are structural motifs abundantly distributed in natural products characterized by a wide spectrum of different biological activities [1]. For instance, Furomollugin and Rubicordifolin, isolated from the roots of *Rubia Cordifolia*, show activity against the hepatitis B virus and cytotoxicity against some tumoral cell lines [2]; Balsaminone A, isolated from the fruit of *Impatiens balsamina L.*, is used for the treatment of articular rheumatism, bruises, and beriberi in Chinese traditional medicine [3], while Xylarianaphthol-1, isolated from a marine sponge-derived fungus of order *Xylariales*, is a p21 activator promoter and contributes to cancer prevention and treatment [4] (Figure 1).

**Figure 2:**
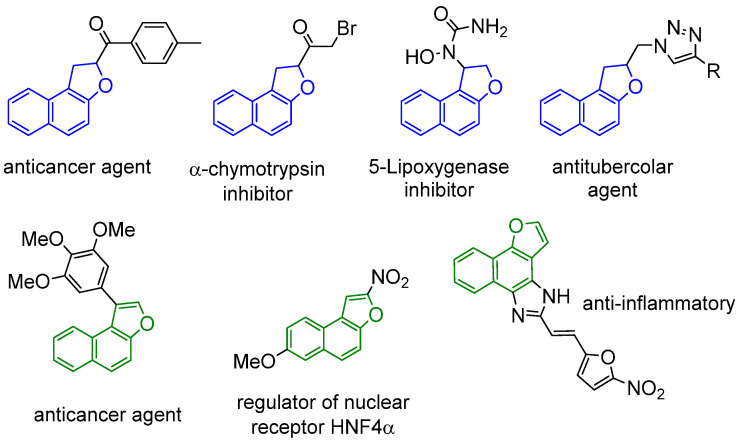
Examples of pharmacologically relevant naphthofurans.

Although in nature naphtho[1,2-*b*]furans and 2,3-dihydronaphtho[1,2-*b*]furans, such as those in Figure 1, are more common, in synthetic pharmacologically-active compounds, naphtho- and 1,2-dihydronaphtho[2,1-*b*]furan derivatives are more diffuse and exhibit a broad spectrum of different biological properties, such as anticancer [5,6]; anti-inflammatories [7] or antitubercular agents [8]; regulators of nuclear receptor HNF4-α [9]; and inhibitors of α-chymotrypsin, lipoxygenase, and C_17,20_ lyase [10,11,12] (Figure 2).

**Figure 3:**
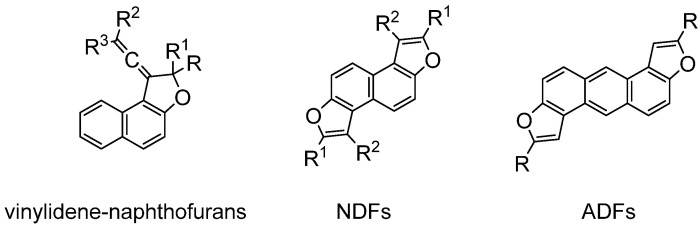
Optically interesting naphthofurans.

Furthermore, the importance of these scaffolds is also due to the optical properties of some particular compounds, such as, for instance, vinylidene-naphthofurans, which show photochromism at room temperature in the solid state when adsorbed on silica gel or exposed to UV or sunlight [13], or NDFs (naphthodifurans) and ADFs (anthradifurans), which have been studied for their applications as hole-transporting materials and photosensitizers [14] (Figure 3).

**Scheme 1:**
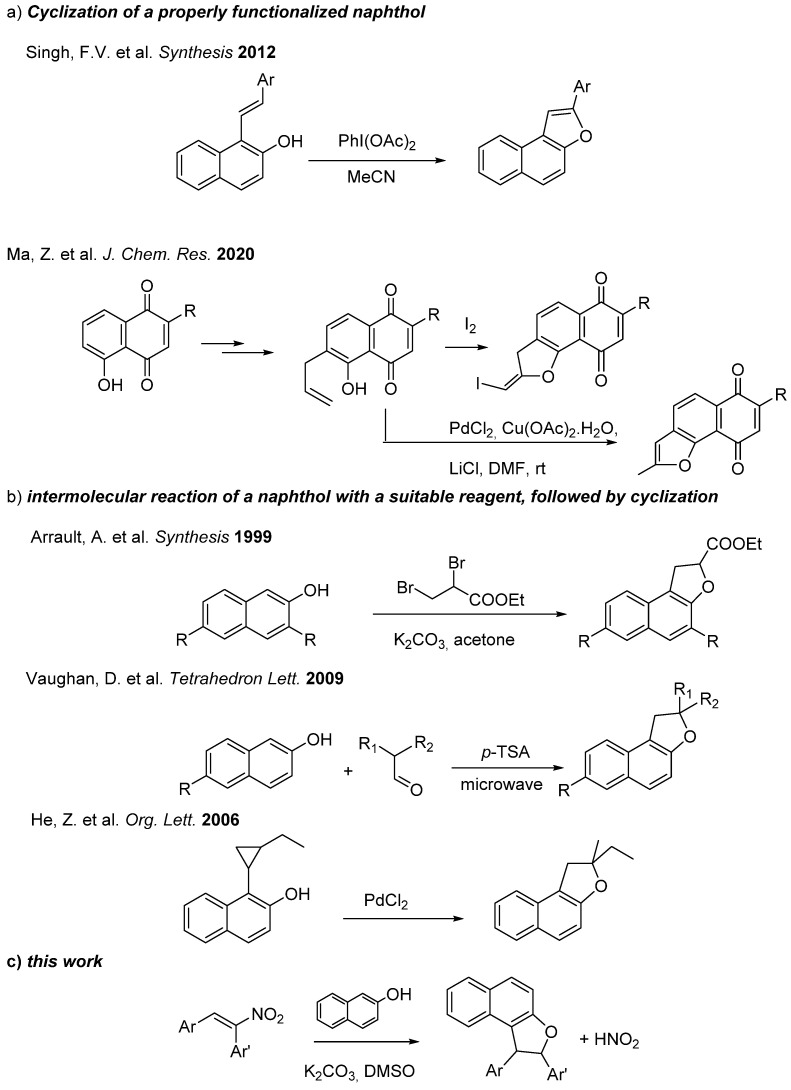
Literature-reported methods [15,17,18,19,21] (**a)**, (**b**) and the approach of the present work (**c**).

Due to their wide range of applications, many synthetic methods for the preparation of naphtho- and dihydronaphthofuran derivatives have been reported (Scheme 1)—the most common involving either the cyclization of a properly functionalized naphthol [15,16,17] or the two-step intermolecular reaction of a naphthol with a suitable reagent, followed by cyclization to give the final heterocyclic system. More often, basic conditions are employed to improve the reactivity of naphthol as double nucleophile, as, for instance, in the classical approach of the annulation of 2-naphthol with dihalo compounds [18]. However, the use of *p*-TSA [19,20] or of metal catalysis [21,22,23,24,25,26,27,28] has also been pursued.

However, since most of the protocols reported in the literature [29,30] suffer from some limitations, such as, for instance, the substrate scope and the possibility to obtain differently functionalized products, the development of more versatile methods is still a topic of interest. In this context, nitrostyrenes have been considered as useful annulation reagents in the reaction with naphthols, because of their ability to react as double electrophiles with the bidentate naphthol enolate, generated in situ under basic conditions. Particular functionalization of the nitro-compound, as, for example, in Morita-Baylis-Hillman [31], together with different reaction conditions, could lead to naphthodihydrofuran or to naphthofuran derivatives, in this case, occasionally with the help of an increase of temperature.

As a matter of fact, starting from a pioneering report [32], during our previous studies, while exploring the reactivity of different nucleophiles towards nitrobutadienes generated by an initial ring-opening of nitrothiophenes [33,34,35], we obtained a great variety of nitrogen-containing heterocycles, such as methyleneazetidines [36], pyrroles [37], pyrazoles [38], carbazoles [39], imidazo[1,2-*a*]pyridines [40], and nitroindoles [41]. However, the involvement of nucleophiles able to generate O-heterocycles, as, for instance, enols, has not been investigated so far. In order to fill the gap and to obtain furan-fused derivatives, the reaction between nitrobutadienic building blocks and aromatic enolates has been now thoroughly investigated and relevant results are reported hereinafter.

## 2. Results

**Scheme 2:**
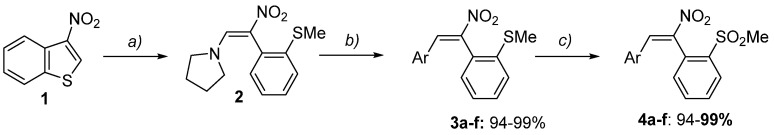
Synthetic sequence to prepare sulfanes **3a–f** and sulfones **4a–f**: (**a**) Pyrrolidine (2 mol eq.)/AgNO_3_ (2 mol. eq.), abs. EtOH, rt, overnight; then MeI (excess), 0 °C to rt, 2h; (**b**) ArMgX or ArLi (1.1 mol eq.), THF, −78 °C, 15–45 min, then acidic quenching; (**c**) MCPBA (2 mol eq.), DCM, rt.

Within this context, we considered of particular interest the hypothesizable outcome of the double nucleophilic attack of *β*-naphthol on **4a** (Ar = *p*-tol). This particular substrate actually is not exactly a nitrobutadiene, but rather a nitrostilbene, deriving from the initial ring-opening of 3-nitrobenzo[b]thiophene with pyrrolidine [42] (Scheme 2). From the ring-opening product **2**, sulfane **3a** (Ar = *p*-tol) originates by reaction with *p*-tolyl magnesium bromide; the following oxidation at sulfur with MCPBA produces sulfone **4a**. Therefore, the nitrobutadienes **3a** and **4a** retain the reactivity of the nitrovinyl moiety, gaining at the same time stability, thanks to the inclusion of the conjugated double bond within a benzene ring.

**Scheme 3:**
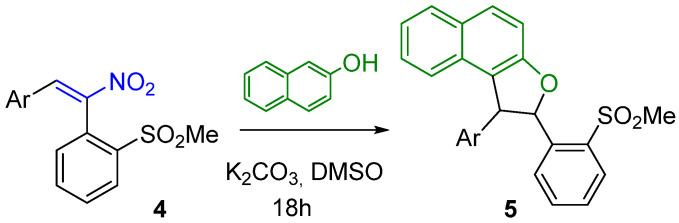
Reaction conditions to obtain naphthodihydrofurans **5a–f**.

Quite rewardingly, a TLC analysis of the reaction mixture obtained after 18 h in the conditions of Scheme 2 showed the absence of the substrate and the presence of a single product as a white solid. Spectroscopic analysis (^1^H and ^13^C NMR, MS) revealed the structure of **5a** (Scheme 3, Ar = *p*-tol), isolated in excellent yields as a single diastereoisomer, without need of purification by column chromatography.

**Figure 4:**
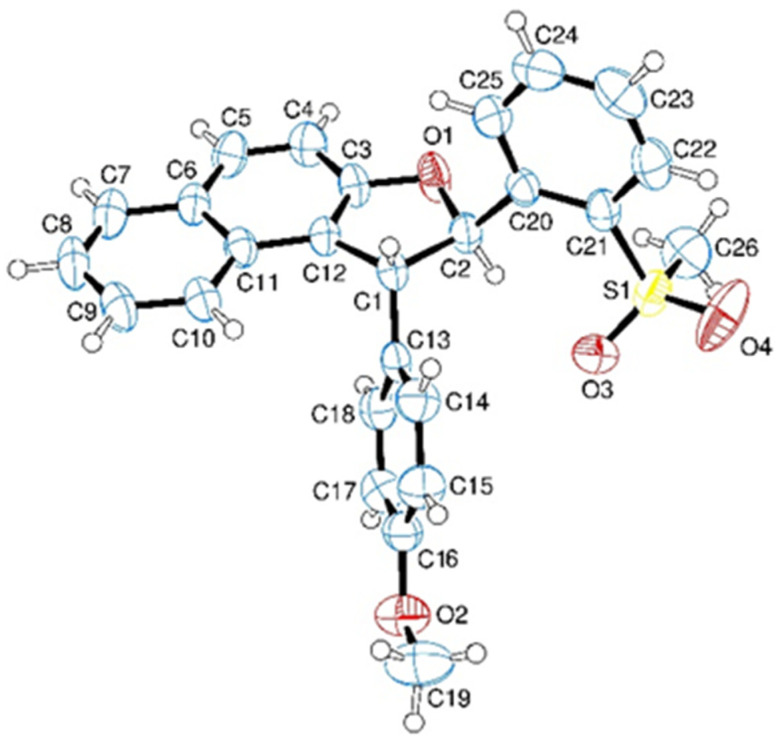
ORTEP obtained by X-Ray analysis of compound **5a**.

We thus considered it worthwhile to investigate the scope of this reaction as to the Ar moiety, introducing groups with different electronic or steric features. Sulfanes **3b–f** and sulfones **4b–f** were prepared according to Scheme 1, and the latter were initially reacted with *β*-naphthol (Scheme 3). The obtained results, reported in Table 1, show a generally very good behaviour of **4**, whatever the aryl substituent. In each case, the obtained diastereoisomer revealed at the ^1^HNMR analysis a *J* of about 5–7 Hz for the dihydrofuran moiety, corresponding presumably to the anti-configuration, as expected on the grounds of a process under thermodynamic control. In order to ascertain without any doubt the configuration of the isolated diastereoisomer, the structure of compound **5a** was confirmed by X-ray analysis (Figure 4).

**Scheme 4:**
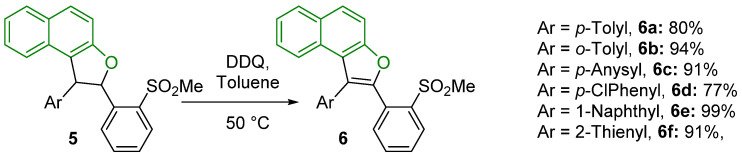
Conditions employed for the oxidative aromatization of **5a–f** to **6a–f**. Yields are given in chromatographically pure compounds (1 spot).

Having these (2,3)-diaryl substituted naphthodihydrofurans in hand, we thought it could be of interest to attempt their oxidative aromatization to the corresponding naphthofurans, with the aim of extending the conjugated system, in search of better optical properties. The aromatization to **6a–f** proceeded very easily with 3 mol eq. of DDQ in toluene at 50 °C, and furnished generally, after chromatographic separation, rather satisfying yields (Scheme 4).

**Figure 5:**
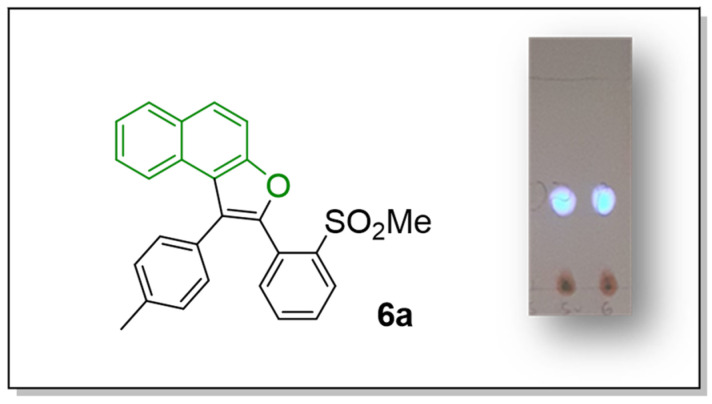
Fluorescence of compound **6a**.

As hoped, after the extension of the conjugated system, we actually observed the appearance of optical properties for the aromatized compounds. The model **6a** revealed a good fluorescence when irradiated under the UV lamp (Figure 5), which encourages a deeper study in the future of the fluorescence properties of these new naphthofuran derivatives.

**Scheme 5:**
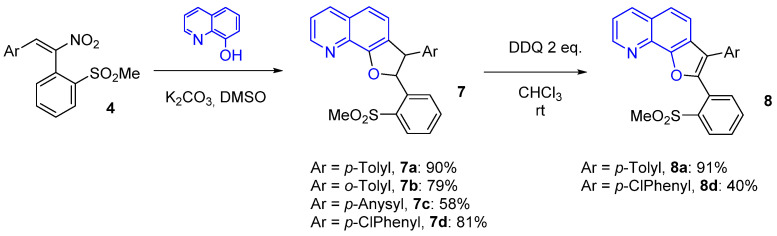
Extension of procedures illustrated in Scheme 2 and Scheme 3, employing 8-hydroxyquinoline as double nucleophile.

Encouraged to proceed in our study, we changed the aromatic enol moving to a quinoline derivative, so as to introduce a further heteroatom in the system. 8-Hydroxyquinoline was our next choice as promising reagent to obtain angular dihydrofuro- and furo[3,2-*h*]quinolines, a kind of fusion not so often reported in the literature. Under the same conditions employed for 2-naphthol, the reactions proceeded faster, but yields in the desired product were generally lower (Scheme 5; cf. yields in Table 1), due to the presence in the final mixture of minor components that were necessary to remove by chromatographic purification.

When trying to aromatize the obtained dihydrofuroquinoline **7a** according to the conditions reported in Scheme 4, the reaction proceeded quickly, but a TLC analysis of the final reaction mixture revealed an extensive decomposition. Such failure could probably be attributed to the presence of the oxidizable aza group. A search for adequate conditions compatible with the presence of the N-heterocycle brought us to use only 2 mol eq. of the same oxidant DDQ, in a different solvent (CHCl_3_), at room temperature; the desired furoquinoline was thus obtained within 24 h after chromatography in 91% yields. These successful conditions were then applied to dihydrofuroquinoline **7d**, furnishing in this case only a lower yield of **8d** (Scheme 5).

**Figure 6:**
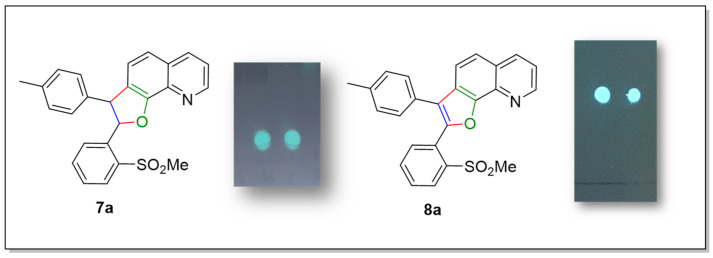
Fluorescence of compounds **7a** and **8a**.

It is interesting to note that both dihydrofuroquinoline **7a** and furoquinoline **8a** show fluorescent properties under UV light, at a different wavelength compared to the naphthofuran derivative **6a** (Figure 6). We intend to develop the study of fluorescence for derivatives **7** and **8** in the future.

**Scheme 6:**
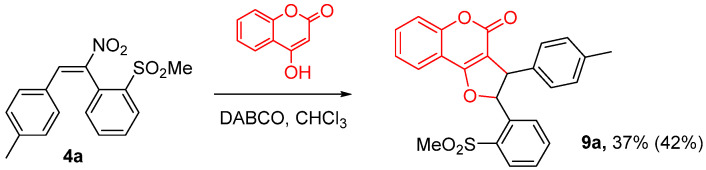
Reaction of compound **4a** with 4-hydroxycoumarin (42% yield on the reacted substrate).

In order to obtain a different polyheterocyclic system, we turned our attention to another phenolic nucleophile, e.g., 4-hydroxycoumarin, whose behaviour was studied with the model nitrostilbene **4a**. Under the usual reaction conditions (K_2_CO_3_ in DMSO) the process proved to be very slow, and the desired product was not present in the final reaction mixture, probably due to the prevalence of alternative pathways, such as, for instance, the hydrolysis of the coumarin ring. Searching for a suitable system, we performed the reaction in the presence of an organic base (DABCO) in a less polar solvent (CHCl_3_), observing in this case the dihydro-4H-furochromenone derivative **9a** in a complex reaction mixture, containing also unreacted starting material (13%); the reaction is still under investigation in order to optimize the conditions and effectively improve the yield (Scheme 6).

**Scheme 7:**
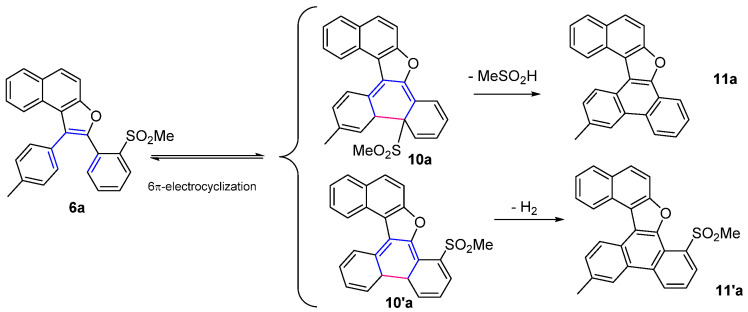
Hypothesizable electrocyclized products **10a** and **10′a** from **6a**, and their following evolution to aromatized **11a** and **11′a**.

As a further evolution for naphthofurans **6a–f** and furoquinolines **8a,d** we devised an electrocyclization process in order to increase the complexity of our polyheterocycles systems. To undergo the pericyclic reaction, the 6π-conjugated system includes three “aromatic” double bonds, e.g., that of the furan ring, and those of the 3-aryl and the 2-(2-methylsulfonyl)phenyl substituents. Due to the presence of two non-equivalent *ortho* positions in the sulfonylated ring, two different electrocyclization products could in principle be expected (**10a** and **10′a**) (Scheme 7), from which two aromatized systems (**11a** and **11′a**) could in turn originate, via methanesulfinic acid elimination or rather by dehydrogenation, respectively. Both processes seemed to be of potential interest, so we proceeded to find the adequate conditions.

**Scheme 8:**
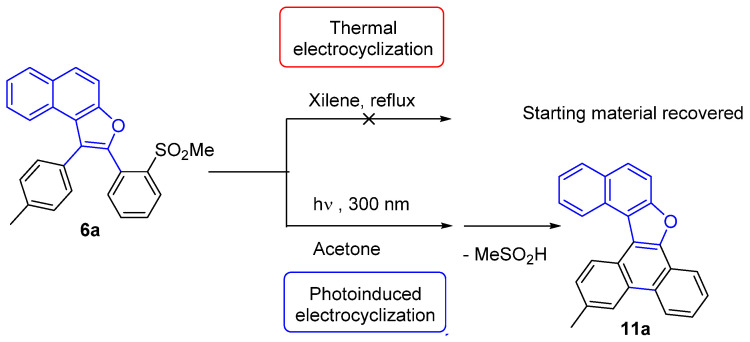
First attempts to obtain electrocyclization products from **6a** by a thermally or a photochemically induced reaction.

A thermic approach on the model substrate **6a**, treated in xylene at reflux, after 24 h, revealed by TLC that only starting material was present; evidently, the attainable temperature (140 °C) was not sufficient to induce the pericyclic process. We thus turned to the alternative photoinduction and, on the grounds of our previous experience and of literature data, we dissolved **6a** in acetone and introduced the sample in a Rayonet reactor (λ = 300 nm): under these conditions, the pericyclic process went straight on, and, thanks to MeSO_2_H elimination, the aromatized polyheterocycle **11a** was directly generated in satisfactory yield (63%) within the same (24 h) period of time (Scheme 8).

**Figure 7:**
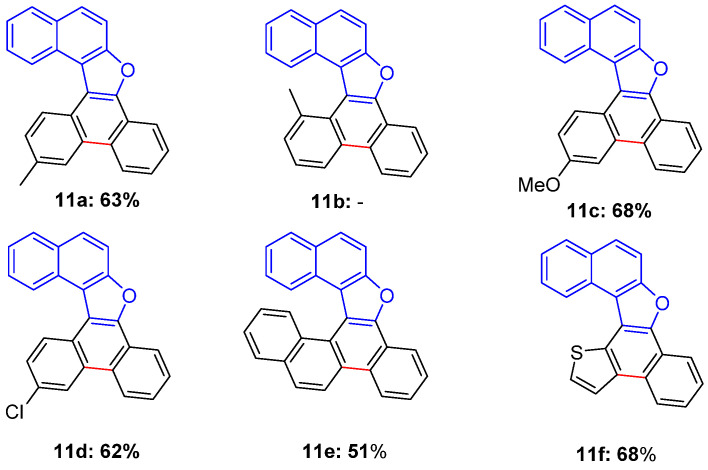
Products obtained from the photochemically induced electrocyclization of compounds **6a, c–f**, followed by aromatization, thanks to MeSO_2_H elimination. Yields refer to chromatographically purified products (1 spot in TLC).

The photoinduced process was then extended to the whole **6b–f** series, obtaining in each case (but for **6b**) good yields in the aromatized products **11c–f** (Figure 7), without isolation of the alternative polycycles **11′c–f** or of the corresponding cyclohexadiene intermediates **10** or **10′**.

**Scheme 9:**
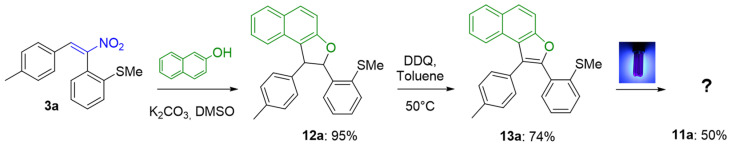
Applying the described sequence to sulfane **3a**.

In order to test the role of the methylsulfonyl group of **6** in the electrocyclization, we prepared the corresponding sulfane derivative **13a** by applying to **3a** the procedure of Scheme 3 and aromatizing the resulting **12a**. Interestingly enough, when we applied the photoinduced process to **13a**, the product isolated was identical to that isolated from **6a**, e.g., the full aromatic polycycle **11a** (Scheme 9).

## 3. Materials and Methods

^1^H NMR and ^13^C NMR spectra were recorded with a JEOL JNM-ECZR 400 spectrometer, at 400 and 100 MHz, respectively; chemical shifts (TMS as internal reference) are reported as δ values (ppm). Signals are designated as follows: s, singlet; d, doublet; dd, doublet of doublets; ddd, doublet of doublet of doublets; t, triplet; tt, triplet of triplets; m, multiplet; and br, broad. Gas chromatography–mass spectrometry (GC-MS) was performed on HP 5890/5971 (EI 70 eV) system equipped with a HP-1 MS capillary column (12 m × 0.2 mm i.d × 0.33 μm). High-resolution mass spectra (HRMS) were obtained with an Agilent MSD TOF mass spectrometer and recorded in positive ion mode with an electrospray (ESI) source. IR spectra were recorded on a Perkin Elmer Spectrum 65 FT-IR and wave numbers are reported in cm^−1^. Melting points were determined with a Büchi 535 apparatus and are uncorrected. Petroleum ether and light petroleum refer to the fractions with bp 40–60 °C and 80–100 °C, respectively. Silica gel 230–400 mesh or neutral alluminium oxide Acros Organics (50–200 μm), were used for column chromatography. TLC analysis were performed on commercially prepared 60 F254 silica gel plates and visualized by UV irradiation (eluant: petroleum ether—ethyl acetate). All commercially available reagents were used as received. The 3-nitrobenzothiophene **1** [43], (*E*)-1-(2-(2-(methylthio)phenyl)-2-nitrovinyl)pyrrolidine **2 [42]**, nitrovinyl derivatives **3a, b, d–f** and **4a, b, d–f** were obtained usually in high yields, as already reported [44]. Compounds **3c** and **4c** were prepared following the same procedure.

(*E*)-(2-(2-(4-Methoxyphenyl)-1-nitrovinyl)phenyl)(methyl)sulfane (**3c**)

Yellow solid. M.p. 87.3–88.4 °C (taken-up with petroleum ether/DCM). ^1^H NMR (400 MHz, CDCl_3_) δ 2.41 (s, 3H), 3.78 (s, 3H), 6.76 (AA’ of AA’BB’, *J* = 8.0 Hz, 2H), 7.05 (BB’ of AA’BB’, *J* = 8.0 Hz, 2H), 7.21 (app dd, 1H), 7.27 (app td, 1H), 7.42 (app dd, 1H), 7.51 (app td, 1H), 8.30 (s, ^1^H). ^13^C NMR (101 MHz, CDCl_3_) δ 15.98 (CH_3_), 55.58 (OCH_3_), 114.57 (Ar CH), 114.89 (Ar CH), 116.11 (Ar CH), 123.59 (Ar C), 126.01 (Ar CH), 126.71 (Ar CH), 130.31 (Ar C), 130.85 (Ar CH), 131.27 (Ar CH), 133.23(Ar CH), 136.49 (Ar CH), 140.28 (Ar C), 162.21 (Ar C-OMe). HRMS (ESI) *m*/*z* calculated [M + H]^+^ C_16_H_16_NO_3_S^+^ 302.0846, found 302.0932.

(*E*)-1-(2-(4-Methoxyphenyl)-1-nitrovinyl)-2-(methylsulfonyl)benzene (**4c**)

Yellow solid. M.p. 142.3–143.5 °C (ethanol). ^1^H NMR (400 MHz, CDCl_3_) δ 2.96 (s, 3H), 3.78 (s, 3H), 6.75 (AA’ of AA’BB’, *J* = 8.0 Hz, 2H), 6.95 (BB’ of AA’BB’, *J* = 8.0 Hz, 2H), 737–7.42 (m, 1H), 7.72–7.80 (m, 2H), 8.21–8.25 (m, 1H), 8.35 (s, 1H). ^13^C NMR (101 MHz, CDCl_3_) δ 44.08, 55.63, 114.88, 123.16, 130.95, 131.06, 131.52, 133.63, 133.66, 134.91, 136.40, 140.51, 144.45, 162.37. HRMS (ESI) *m*/*z* calculated [M + H]^+^ C_16_H_16_NO_5_S^+^ 334.0744, found 334.0623.

### 3.1. General Procedure for the Reactions of Substrates **4** with β-Naphthol

In a flask, the relevant nitrostilbene **4** (0.30 mmol) was dissolved in DMSO (2.0 mL), then 2-naphthol (0.30 mmol, 43.4 mg) and K_2_CO_3_ (0.38 mmol, 53 mg) were added as solids under magnetic stirring. The reaction was stirred at room temperature and monitored by TLC. Upon completion (24 h), the mixture was diluted with 20 mL of ethyl acetate, washed with HCl 1M (2 × 5 mL), NaHCO_3_ (2 × 5 mL), water (1 × 5 mL) and brine (1 × 5 mL). The organic phase was dried with anhydrous Na_2_SO_4_, and the solvent evaporated, to give the products **5a–f**, with no need of further purification.

#### 2-(2-(Methylsulfonyl)phenyl)-1-(*p*-tolyl)-1,2-dihydronaphtho[2,1-*b*]furan (**5a**)

White solid. M.p. 171.2–173.0 °C (taken-up with petroleum ether/DCM). ^1^H NMR (400 MHz, CDCl_3_) δ 2.29 (s, 3H), 2.85 (s, 3H), 5.03 (d, *J* = 5.9 Hz, 1H), 6.66 (d, *J* = 5.9 Hz, 1H), 7.11 (q, *J* = 7.9 Hz, 4H), 7.23–7.34 (m, 5H), 7.49–7.56 (m, 1H), 7.57–7.64 (m, 1H), 7.64–7.70 (m, 1H), 7.79–7.87 (m, 2H), 8.10 (d, *J* = 7.9 Hz, 1H). ^13^C NMR (101 MHz, CDCl_3_) δ 21.28, 45.48, 57.52, 88.07, 112.07, 120.84, 122.97, 123.34, 127.05, 128.10, 128.73, 129.00, 129.22, 129.53, 129.75, 130.27, 130.63, 130.76, 134.52, 137.18, 138.40, 139.14, 141.19, 157.39. HRMS (ESI) *m*/*z* calculated [M + H]^+^ C_26_H_23_O_3_S^+^ 415.1368, found 415.1380.

#### 2-(2-(Methylsulfonyl)phenyl)-1-(*o*-tolyl)-1,2-dihydronaphtho[2,1-*b*]furan (**5b**)

White solid. M.p. 224.0–226.4 °C (taken-up with petroleum ether/DCM). ^1^H NMR (400 MHz, CDCl_3_) δ 2.20 (s, 3H), 2.84 (s, 3H), 5.28–5.52 (m, 1H), 6.53–6.69 (m, 1H), 7.01 (s, 1H), 7.08 (t, *J* = 6.9 Hz, 1H), 7.11–7.21 (m, 3H), 7.22–7.33 (m, 3H), 7.55 (td, *J* = 7.7, 1.4 Hz, 1H), 7.65 (td, *J* = 7.6, 1.5 Hz, 1H), 7.77 (dd, *J* = 7.9, 1.4 Hz, 1H), 7.82–7.88 (m, 2H), 8.13 (dd, *J* = 8.1, 1.5 Hz, 1H). ^13^C NMR (101 MHz, CDCl_3_) δ 20.04, 45.35, 53.12, 87.84, 112.17, 120.82, 123.05, 123.39, 127.12, 127.37, 128.05, 128.87, 129.04, 129.40, 129.53, 130.25, 130.47, 130.72, 134.65, 135.76, 138.60, 140.21, 141.19, 154.96, 157.69 (two carbons are accidentally isochronous). HRMS (ESI) *m*/*z* calculated [M + H]^+^ C_26_H_23_O_3_S^+^ 415.1362, found 415.1393.

#### 1-(4-Methoxyphenyl)-2-(2-(methylsulfonyl)phenyl)-1,2-dihydronaphtho[2,1-*b*]furan (**5c**)

White solid. M.p. 178.2–178.5 (ethanol). ^1^H NMR (400 MHz, CDCl_3_) δ 2.87 (s, 3H), 3.76 (s, 3H), 5.03 (d, *J* = 6.0 Hz, 1H), 6.65 (d, *J* = 6.0 Hz, 1H), 6.82 (d, *J* = 8.7 Hz, 2H), 7.16 (d, *J* = 8.7 Hz, 2H), 7.23–7.33 (m, 4H), 7.53 (ddd, *J* = 7.8, 7.2, 1.5 Hz, 1H), 7.60 (td, *J* = 7.5, 1.4 Hz, 1H), 7.66 (dd, *J* = 7.8, 1.5 Hz, 1H), 7.80–7.86 (m, 2H), 8.11 (dd, *J* = 8.0, 1.4 Hz, 1H). ^13^C NMR (101 MHz, CDCl_3_) δ 45.54, 55.33, 57.16, 88.12, 112.09, 114.41, 120.89, 122.99, 123.37, 127.06, 128.76, 129.03, 129.23, 129.32, 129.57, 130.33, 130.65, 130.79, 134.26, 134.53, 138.48, 141.22, 157.36, 158.98. HRMS (ESI) *m*/*z* calculated [M + H]^+^ C_26_H_23_O_4_S^+^ 431.1317, found 431.1332.

#### 1-(4-Chlorophenyl)-2-(2-(methylsulfonyl)phenyl)-1,2-dihydronaphtho[2,1-*b*]furan (**5d**)

Whitish solid. M.p. 140.1–142.5 °C (ethanol/dioxane). ^1^H NMR (400 MHz, CDCl_3_) δ 2.98 (s, 3H), 4.99 (d, *J* = 5.1 Hz, 1H), 6.59 (d, *J* = 5.1 Hz, 1H), 7.17 (d, *J* = 8.5 Hz, 2H), 7.22–7.32 (m, 6H), 7.49–7.62 (m, 3H), 7.80–7.87 (m, 2H), 8.07–8.12 (m, 1H). ^13^C NMR (101 MHz, CDCl_3_) δ 45.58, 57.01, 87.88, 112.00, 120.49, 122.81, 123.58, 127.30, 128.36, 129.10, 129.16, 129.36, 129.58, 129.70, 130.34, 130.50, 131.13, 133.30, 134.58, 138.16, 140.83, 140.97, 157.46. HRMS (ESI) *m*/*z* calculated [M + H]^+^ C_25_H_20_ClO_3_S^+^ 435.0822, found 435.0844.

#### 2-(2-(Methylsulfonyl)phenyl)-1-(naphthalen-1-yl)-1,2-dihydronaphtho[2,1-*b*]furan (**5e**)

Waxy solid. ^1^H NMR (400 MHz, CDCl_3_) δ 2.52 (s, 3H), 6.07 (d, *J* = 6.2 Hz, 1H), 6.69 (d, *J* = 6.2 Hz, 1H), 7.10–7.24 (m, 3H), 7.27–7.37 (m, 4H), 7.47 (t, *J* = 7.5 Hz, 1H), 7.56–7.63 (m, 1H), 7.63–7.74 (m, 2H), 7.77 (d, *J* = 8.2 Hz, 1H), 7.83–7.95 (m, 4H), 8.10 (d, *J* = 7.8 Hz, 1H). ^13^C NMR (101 MHz, CDCl_3_) δ 45.16, 51.85, 87.99, 112.48, 120.30, 122.85, 123.43, 125.99, 126.15, 126.23, 126.57, 127.12, 128.07, 128.85, 129.10, 129.27, 129.61, 129.77, 130.32, 130.54, 130.96, 131.59, 134.05, 134.81, 137.94, 139.07, 140.92, 157.89 (one carbon is accidentally isochronous). HRMS (ESI) *m*/*z* calculated [M + H]^+^ C_29_H_23_O_3_S^+^ 451.1367, found 451.1390.

#### 2-(2-(Methylsulfonyl)phenyl)-1-(thiophen-2-yl)-1,2-dihydronaphtho[2,1-*b*]furan (**5f**)

White solid. M.p. 126.1–127.7 °C (ethanol). ^1^H NMR (CDCl_3_, 400 MHz) δ 2.97 (s, 3H), 5.40 (d, *J* = 5.8 Hz, 1H), 6.72 (d, *J* = 5.9 Hz, 1H), 6.89–6.99 (m, 2H), 7.19 (dd, *J* = 4.9, 1.4 Hz, 1H), 7.24 (d, *J* = 8.9 Hz, 1H), 7.27–7.38 (m, 2H), 7.45 (dd, *J* = 8.1, 1.5 Hz, 1H), 7.50–7.64 (m, 3H), 7.85 (d, *J* = 8.3 Hz, 2H), 8.13 (dd, *J* = 7.8, 1.4 Hz, 1H). ^13^C NMR (CDCl_3_, 101 MHz) δ 45.52, 52.44, 88.04, 112.10, 120.13, 122.70, 123.53, 125.25, 125.95, 127.14, 127.25, 128.65, 129.06, 129.42, 129.71, 130.28, 130.60, 131.26, 134.52, 138.60, 140.42, 145.48, 157.12. HRMS (ESI) *m*/*z* calculated [M + H]^+^ C_23_H_19_O_3_S_2_^+^ 407.0775, found 407.0799.

### 3.2. General Procedure for the Oxidative Aromatization Reaction of Dihydronaphthofurans **5a–f** to Naphthofurans **6a–f**

The relevant dihydrofuran **5** (0.17 mmol) was dissolved in toluene (3.0 mL) and DDQ (0.51 mmol, 116 mg) was added. The reaction was stirred at 50 °C in an oil bath and monitored by TLC. After the disappearance of the starting material (24 h), the mixture was filtered, and then the filtrate was washed with dichloromethane and toluene, and then with saturated Na_2_CO_3_ (2 × 5 mL), water (1 × 5 mL) and brine (1 × 5 mL). The organic phase was dried with anhydrous Na_2_SO_4_ and the solvent evaporated to give the crude **7**, which was purified by column chromatography (petroleum ether/ethyl acetate 1:1 to 1:2).

#### 2-(2-(Methylsulfonyl)phenyl)-1-(*p*-tolyl)naphtho[2,1-*b*]furan (**6a**)

White solid. M.p. 208.5–209.5 °C (ethanol). ^1^H NMR (CDCl_3_, 400 MHz) δ 2.41 (s, 3H), 3.41 (s, 3H), 7.21 (d, *J* = 7.7 Hz, 2H), 7.28–7.37 (m, 2H), 7.39–7.50 (m, 4H), 7.51–7.57 (m, 1H), 7.67 (d, *J* = 8.9 Hz, 1H), 7.80 (d, *J* = 8.7 Hz, 1H), 7.87 (dd, *J* = 8.4, 0.7 Hz, 1H), 7.94 (dd, *J* = 8.1, 0.7 Hz, 1H), 8.21 (dd, *J* = 7.9, 1.4 Hz, 1H). ^13^C NMR (CDCl_3_, 101 MHz) δ 21.52, 45.37, 112.00, 122.26, 122.58, 123.52, 124.63, 126.23, 126.58, 128.69, 129.06, 129.52, 129.62, 129.94, 130.38, 130.54, 130.79, 131.19, 133.19, 133.90, 137.72, 140.70, 148.61, 152.16. HRMS (ESI) *m*/*z* calcd. For C_26_H_21_O_3_S [M + H]^+^: 413.1211, found 413.1230.

#### 2-(2-(Methylsulfonyl)phenyl)-1-(*o*-tolyl)naphtho[2,1-*b*]furan (**6b**)

White solid. M.p. 221.4–222.9 °C (ethanol). ^1^H NMR (CDCl_3_, 400 MHz) δ 2.21 (s, 3H), 3.51 (s, 3H), 7.22 (td, *J* = 6.9, 6.4, 2.6 Hz, 1H), 7.27–7.36 (m, 4H), 7.38–7.55 (m, 5H), 7.69 (d, *J* = 8.9 Hz, 1H), 7.81 (d, *J* = 8.9 Hz, 1H), 7.94 (d, *J* = 8.8 Hz, 1H), 8.21 (dd, *J* = 7.8, 1.4 Hz, 1H). ^13^C NMR (CDCl_3_, 101 MHz) δ 20.28, 45.57, 112.03, 121.37, 122.78, 122.88, 124.68, 126.28, 126.63, 128.54, 128.78, 128.96, 129.54, 130.33, 130.70, 131.03, 131.54, 132.45, 132.89, 133.23, 138.43, 140.13, 148.36, 152.14 (two carbons are accidentally isochronous). HRMS (ESI) *m*/*z* calcd. For C_26_H_21_O_3_S [M + H]^+^: 413.1211, found 413.1242.

#### 1-(4-Methoxyphenyl)-2-(2-(methylsulfonyl)phenyl)naphtho[2,1-*b*]furan (**6c**)

White solid. M.p. 199.8–200.2 °C (ethanol). ^1^H NMR (CDCl_3_, 400 MHz) δ 3.42 (s,3H), 3.86 (s, 3H), 6.94 (d, *J* = 7.9 Hz, 2H), 7.29 (dt, *J* = 7.7, 1.2 Hz, 1H), 7.31–7.37 (m, 1H), 7.40–7.50 (m, 4H), 7.50–7.58 (m, 1H), 7.67 (dd, *J* = 8.9, 0.9 Hz, 1H), 7.80 (d, *J* = 9.0 Hz, 1H), 7.87 (dt, *J* = 8.4, 1.1 Hz, 1H), 7.94 (d, *J* = 8.2 Hz, 1H), 8.21 (d, *J* = 7.8 Hz, 1H). ^13^C NMR (CDCl_3_, 101 MHz) δ 45.39, 55.38, 112.01, 114.25, 121.93, 122.67, 123.46, 124.64, 125.09, 126.27, 126.56, 128.70, 129.07, 129.61, 130.40, 130.57, 131.18, 132.09, 133.20, 133.90, 140.71, 148.69, 152.14, 159.39. HRMS (ESI) *m*/*z* calcd. for C_26_H_21_O_4_S [M + H]^+^: 429.1161, found 429.1182.

#### 1-(4-Chlorophenyl)-2-(2-(methylsulfonyl)phenyl)naphtho[2,1-*b*]furan (**6d**)

White solid. M.p. 215.0–216.3 °C (ethanol). ^1^H NMR (CDCl_3_, 400 MHz) δ 3.42 (s, 3H), 7.23–7.27 (m, 1H), 7.34–7.41 (m, 3H), 7.43–7.53 (m, 4H), 7.58 (td, *J* = 7.7, 1.4 Hz, 1H), 7.67 (d, *J* = 9.0 Hz, 1H), 7.78–7.84 (m, 2H), 7.96 (dt, *J* = 8.2, 1.0 Hz, 1H), 8.22 (dd, *J* = 7.9, 1.4 Hz, 1H). ^13^C NMR (CDCl_3_, 101 MHz) δ 45.47, 111.97, 121.09, 122.20, 123.26, 124.82, 126.47, 126.84, 128.44, 129.10, 129.21, 129.99, 130.11, 130.52, 131.22, 131.69, 132.32, 133.37, 133.80, 134.11, 140.83, 148.93, 152.24. HRMS (ESI) *m*/*z* calcd. for C_25_H_18_ClO_3_S [M + H]^+^: 433.0665, found 433.0696.

#### 2-(2-(Methylsulfonyl)phenyl)-1-(naphthalen-1-yl)naphtho[2,1-*b*]furan (**6e**)

Waxy solid. ^1^H NMR (CDCl_3_, 400 MHz) δ 3.52 (s, 3H), 7.02–7.20 (m, 4H), 7.35 (ddt, *J* = 8.3, 3.4, 1.5 Hz, 2H), 7.41–7.53 (m, 3H), 7.62 (dd, *J* = 7.0, 1.3 Hz, 1H), 7.75 (d, *J* = 8.9 Hz, 1H), 7.82–7.98 (m, 5H), 8.17 (dd, *J* = 8.0, 1.3 Hz, 1H). ^13^C NMR (CDCl_3_, 101 MHz) δ 45.63, 100.06, 112.01, 120.10, 123.51, 124.62, 125.93, 126.31, 126.34, 126.40, 126.80, 126.94, 128.38, 128.49, 128.81, 128.86, 129.19, 129.50, 129.65, 130.29, 130.54, 131.13, 132.82, 133.22, 133.68, 140.22, 149.64, 152.23. HRMS (ESI) *m*/*z* calcd. for C_29_H_21_O_3_S [M + H]^+^: 449.1211, found 449.1246.

#### 2-(2-(Methylsulfonyl)phenyl)-1-(thiophen-2-yl)naphtho[2,1-*b*]furan (**6f**)

Waxy solid. ^1^H NMR (CDCl_3_, 400 MHz) δ 3.34 (s, 3H), 7.11 (dd, *J* = 5.2, 3.4 Hz, 1H), 7.22 (dd, *J* = 3.5, 1.2 Hz, 1H), 7.36–7.52 (m, 4H), 7.52–7.63 (m, 2H), 7.68 (d, *J* = 8.9 Hz, 1H), 7.82 (d, *J* = 8.9 Hz, 1H), 7.90–7.98 (m, 2H), 8.22 (dd, *J* = 7.6, 1.6 Hz, 1H). ^13^C NMR (CDCl_3_, 101 MHz) δ 45.27, 111.99, 115.12, 122.74, 123.32, 124.89, 126.52, 126.92, 127.28, 127.64, 128.38, 129.09, 129.69, 129.89, 130.12, 130.33, 131.23, 133.10, 133.23, 133.73, 140.78, 150.17, 152.09. HRMS (ESI) *m*/*z* calcd. for C_23_H_17_O_3_S_2_ [M + H]^+^: 405.0619, found 405.0656.

### 3.3. General Procedure for the Reaction of Substrates **4** with 8-Hydroxyquinoline

In a flask, the relevant nitrostilbene **4** (0.30 mmol) was dissolved in DMSO (2 mL) and 8-hydroxyquinoline (0.30 mmol, 44 mg) and K_2_CO_3_ (0.38 mmol, 53 mg) were added as solids under magnetic stirring. The reaction was stirred at room temperature and monitored by TLC. Upon completion (4–5 h), the mixture was diluted with 20 mL of ethyl acetate, washed with HCl 1M (2 × 5 mL), NaHCO_3_ (2 × 5 mL), water (1 × 5 mL), and brine (1 × 5 mL). The organic phase was dried with anhydrous Na_2_SO_4_, and the solvent evaporated. The crude was purified by column chromatography (petroleum ether/ethyl acetate 1:1 to 1:2), thus obtaining compounds **7a–d**. 

#### 2-(2-(Methylsulfonyl)phenyl)-3-(*p*-tolyl)-2,3-dihydrofuro[3,2-*h*]quinoline (**7a**)

White solid. M.p. 202.2–203.0 °C (taken-up with petroleum ether/DCM). ^1^H NMR (CDCl_3_, 400 MHz) δ 2.33 (s, 3H), 3.04 (s, 3H), 4.95 (d, *J* = 7.3 Hz, 1H), 6.71 (d, *J* = 7.3 Hz, 1H), 7.08–7.17 (m, 4H), 7.21 (dd, *J* = 8.2, 0.8 Hz, 1H), 7.40–7.47 (m, 2H), 7.48–7.66 (m, 3H), 8.12 (dt, *J* = 7.8, 1.1 Hz, 1H), 8.18 (dd, *J* = 8.3, 1.7 Hz, 1H), 8.88–8.93 (m, 1H). ^13^C NMR (CDCl_3_, 101 MHz) δ 21.30, 45.73, 58.92, 89.57, 121.38, 121.55, 123.79, 128.35, 129.17, 129.24, 129.54 129.72, 129.98, 134.31, 135.90, 136.37, 137.27, 138.51, 138.65, 140.84, 150.24 (two carbons are accidentally isochronous). HRMS (ESI) *m*/*z* calcd. for C_25_H_22_NO_3_S [M + H]^+^: 416.1320, found 416.1346.

#### 2-(2-(Methylsulfonyl)phenyl)-3-(*o*-tolyl)-2,3-dihydrofuro[3,2-*h*]quinoline (**7b**)

White solid. M.p. 147.0–148.0 °C (ethanol). ^1^H NMR (CDCl_3_, 400 MHz) δ 2.09 (s, 3H), 3.02 (s, 3H), 5.35 (d, *J* = 7.7 Hz, 1H), 6.66 (d, *J* = 7.1 Hz, 1H), 7.16 (s, 4H), 7.21 (d, *J* = 8.2 Hz, 1H), 7.40–7.48 (m, 2H), 7.54 (td, *J* = 7.6, 1.5 Hz, 1H), 7.61 (td, *J* = 7.6, 1.5 Hz, 1H), 7.70 (d, *J* = 7.7 Hz, 1H), 8.12 (dd, *J* = 7.9, 1.5 Hz, 1H), 8.19 (dd, *J* = 8.4, 1.7 Hz, 1H), 8.91 (dd, *J* = 4.2, 1.7 Hz, 1H). ^13^C NMR (CDCl_3_, 101 MHz) δ 19.94, 29.83, 31.37, 45.62, 121.41, 121.54, 123.67, 126.93, 127.49, 128.37, 129.20, 129.34, 129.48, 129.94, 130.61, 134.41, 135.93, 136.38, 136.53, 138.47, 140.90, 150.25, 154.84 (two carbons are accidentally isochronous). HRMS (ESI) *m*/*z* calcd. for C_25_H_22_NO_3_S [M + H]^+^: 416.1320, found 416.1358.

#### 2-(2-(Methylsulfonyl)phenyl)-3-(*p*-anysyl)-2,3-dihydrofuro[3,2-h]quinoline (**7c**)

Yellow solid. ^1^H NMR (CDCl_3_, 400 MHz) δ 3.05 (s, 3H), 3.78 (s, 3H), 4.95 (d, *J* = 7.4 Hz, 1H), 6.68 (d, *J* = 7.4 Hz, 1H), 6.85 (d, *J* = 8.7 Hz, 2H), 7.14 (d, *J* = 8.7 Hz, 2H), 7.21 (dd, *J* = 8.2, 0.7 Hz, 1H), 7.43 (dt, *J* = 8.4, 2.2 Hz, 2H), 7.49–7.65 (m, 3H), 8.12 (dd, *J* = 7.8, 1.4 Hz, 1H), 8.19 (dd, *J* = 8.4, 1.7 Hz, 1H), 8.90 (dd, *J* = 4.3, 1.7 Hz, 1H). ^13^C NMR (CDCl_3_, 101 MHz) δ 45.72, 55.34, 58.51, 89.63, 114.32, 121.38, 121.55, 123.76, 128.41, 129.17, 129.23, 129.50, 129.53, 129.96, 133.64, 134.30, 135.89, 136.37, 138.45, 140.61, 150.24, 154.52, 159.09. HRMS (ESI) *m*/*z* calcd. for C_25_H_22_NO_4_S [M + H]^+^: 432.1269, found 432.1297.

#### 3-(4-Chlorophenyl)-2-(2-(methylsulfonyl)phenyl)-2,3-dihydrofuro[3,2-*h*]quinoline (**7d**)

Yellow solid. M.p. 147.0–148.3 °C (ethanol). ^1^H NMR (CDCl_3_, 400 MHz) δ 3.10 (s, 3H), 4.92 (d, *J* = 6.7 Hz, 1H), 6.63 (d, *J* = 6.7 Hz, 1H), 7.12–7.20 (m, 3H), 7.23–7.33 (m, 3H), 7.39–7.48 (m, 2H), 7.48–7.61 (m, 2H), 8.10 (dt, *J* = 7.5, 1.2 Hz, 1H), 8.19 (dd, *J* = 8.4, 1.7 Hz, 1H), 8.91 (dd, *J* = 4.2, 1.6 Hz, 1H). ^13^C NMR (CDCl_3_, 101 MHz) δ 45.72, 58.60, 89.31, 121.65, 121.74, 123.62, 127.70, 128.86, 129.14, 129.39, 129.63, 129.72, 130.04, 133.51, 134.40, 135.85, 136.43, 138.21, 140.34, 150.38, 154.63 (two carbons are accidentally isochronous). HRMS (ESI) *m*/*z* calcd. for C_24_H_19_ClNO_3_S [M + H]^+^: 436.0774, found 436.0798.

### 3.4. General Procedure for the Oxidative Aromatization Reaction of Dihydrofuroquinolines **7a, d** to Furoquinolines **8a, d**

The relevant dihydrofuran **6** (0.12 mmol) was dissolved in chloroform (3.0 mL) and DDQ (0.24 mmol, 54.5 mg) was added. The reaction was maintained under magnetic stirring at room temperature, until completion (24 h, monitored by TLC). Then, an aqueous saturated NaHCO_3_ solution (8 mL) was added, and the resulting mixture was extracted with CHCl_3_ (3 × 15 mL). The combined organic layers were dried over anhydrous Na_2_SO_4_, filtered, and concentrated in vacuo. The reaction mixture was purified by column chromatography (petroleum ether/ethyl acetate 1:1) to afford the desired products **8a–d**.

#### 2-(2-(Methylsulfonyl)phenyl)-3-(*p*-tolyl)furo[3,2-*h*]quinoline (**8a**)

White solid. M.p. 203.2–205.2 °C (ethanol). ^1^H NMR (CDCl_3_, 400 MHz) δ 2.37 (s, 3H), 3.63 (s, 3H), 7.19 (d, *J* = 7.8 Hz, 2H), 7.35–7.43 (m, 3H), 7.46 (dd, *J* = 8.2, 4.3 Hz, 1H), 7.54 (td, *J* = 7.6, 1.4 Hz, 1H), 7.62 (td, *J* = 7.7, 1.5 Hz, 1H), 7.70 (d, *J* = 8.6 Hz, 1H), 7.85 (d, *J* = 8.5 Hz, 1H), 8.29 (d, *J* = 8.1 Hz, 2H), 8.93 (dd, *J* = 4.4, 1.7 Hz, 1H). ^13^C NMR (CDCl_3_, 101 MHz) δ 21.44, 46.15, 120.12, 120.83, 120.95, 123.58, 126.87, 128.01, 128.43, 129.57, 129.72, 130.02, 130.71, 130.82, 133.42, 133.49, 136.49, 137.10, 137.58, 141.15, 149.38, 149.42, 150.33. HRMS (ESI) *m*/*z* calcd. For C_25_H_20_NO_3_S [M + H]^+^: 414.1163, found 414.1187.

#### 3-(4-Chlorophenyl)-2-(2-(methylsulfonyl)phenyl)furo[3,2-*h*]quinoline (**8d**)

Pale yellow solid. M.p. 227.3–229.0 °C (ethanol). ^1^H NMR (CDCl_3_, 400 MHz) δ 3.63 (s, 3H), 7.26 (s, 2H), 7.33–7.40 (m, 3H), 7.43 (d, *J* = 8.5 Hz, 2H), 7.46–7.51 (m, 1H), 7.57 (td, *J* = 7.5, 1.4 Hz, 1H), 7.65 (td, *J* = 7.8, 1.4 Hz, 1H), 7.73 (d, *J* = 8.6 Hz, 1H), 7.81 (d, *J* = 8.6 Hz, 1H), 8.30 (dd, *J* = 8.2, 1.5 Hz, 2H), 8.94 (dd, *J* = 4.3, 1.7 Hz, 1H).^13^C NMR (CDCl_3_, 101 MHz) δ 46.19, 119.69, 119.94, 121.02, 123.92, 126.97, 127.58, 129.30, 130.01, 130.36, 130.83, 130.99, 133.40, 133.58, 133.82, 136.57, 137.06, 141.27, 149.48, 149.87, 150.50. HRMS (ESI) *m*/*z* calcd. For C_24_H_17_ClN_2_O_3_S [M + H]^+^: 434.0612, found 434.0658.

### 3.5. Procedure for the Reaction of Substrate **4a** with 4-Hydroxycoumarin

In a round-bottom flask the substrate **4a** (0.157 mmol) was dissolved in chloroform (1.5 mL), then 4-hydroxycoumarin (0.157 mmol) and DABCO (0.047 mmol, 30%) were added. The reaction was stirred at room temperature for four days. The crude residue was purified by preparative TLC (petroleum ether/ethyl acetate 1:1).

#### 2-(2-(Methylsulfonyl)phenyl)-3-(*p*-tolyl)-2,3-dihydro-4H-furo[3,2-*c*]chromen-4-one (**9a**) 

Waxy solid. ^1^H NMR (CDCl_3_, 400 MHz) δ 2.31 (s, 3H), 2.75 (s, 3H), 4.67 (d, *J* = 6.4 Hz, 1H), 6.99 (d, *J* = 6.4 Hz, 1H), 7.11–7.22 (m, 4H), 7.35 (dd, *J* = 7.6, 1.1 Hz, 1H), 7.43 (dd, *J* = 8.5, 1.0 Hz, 1H), 7.63 (ddt, *J* = 9.5, 8.1, 1.3 Hz, 3H), 7.73 (ddd, *J* = 18.7, 7.6, 1.5 Hz, 2H), 8.13 (dd, *J* = 8.0, 1.4 Hz, 1H). ^13^C NMR (CDCl_3_, 101 MHz) δ 21.29, 45.59, 55.39, 90.14, 105.69, 112.30, 117.28, 123.25, 124.31, 127.65, 128.54, 129.92, 129.99, 130.06, 133.10, 134.84, 136.46, 137.95, 138.50, 138.96, 155.59, 159.55, 166.13. HRMS (ESI) *m*/*z* calcd. for C_25_H_21_O_5_S [M + H]^+^: 433.1109, found 433.1125.

### 3.6. General Procedure for the 6π-Electrocyclization of Naphthofurans **6a–f**

The relevant naphthofuran **6a–f** (0.072 mmol) was introduced in a 25 mL quartz tube and dissolved in 3.5 mL of acetone (spectroscopic grade); the tube was sealed and placed within a Rayonet photochemical reactor equipped with fourteen 300 nm lamps. The reaction, followed by TLC, required 24–48 h; at the end, the solvent was removed, and the crude **residue** was dissolved in DCM, washed with a NaHCO_3_ saturated solution and water, and then dried over Na_2_SO_4_; after filtration, the solvent was removed under reduced pressure and the residue purified by chromatography on column (petroleum ether/ethyl acetate mixtures as eluent).

#### 13-Methylnaphtho[2,1-*b*]phenanthro[9,10-*d*]furan (**11a**)

White solid. M.p. 137.2–138.4 °C (taken-up with petroleum ether/DCM). ^1^H NMR (CDCl_3_, 400 MHz) δ 2.70 (s, 3H), 7.55–7.67 (m, 2H), 7.68–7.77 (m, 3H), 7.91 (d, *J* = 8.9 Hz, 1H), 7.95 (d, *J* = 8.9 Hz, 1H), 8.08 (dd, *J* = 8.1, 1.4 Hz, 1H), 8.55 (dd, *J* = 7.1, 2.0 Hz, 1H), 8.63 (s, 1H), 8.77 (dd, *J* = 7.4, 1.9 Hz, 1H), 9.04 (d, *J* = 8.3 Hz, 1H), 9.12 (d, *J* = 8.5 Hz, 1H). ^13^C NMR (CDCl_3_, 101 MHz) δ 22.06, 112.92, 116.82, 120.20, 121.58, 122.52, 123.38, 124.08, 124.55, 125.75,126.04, 126.10, 126.27, 126.91, 127.28, 127.81, 128.20, 128.71, 129.01, 129.60, 130.28, 131.50, 134.92, 150.88, 154.25. HRMS (ESI) *m*/*z* calcd. for C_25_H_17_O [M + H]^+^: 333.1279, found 333.1297.

#### 13-Methoxynaphtho[2,1-*b*]phenanthro[9,10-*d*]furan (**11c**)

White solid. M.p. 188.7–190.1 °C (taken-up with petroleum ether/DCM). ^1^H NMR (CDCl_3_, 400 MHz) δ 4.09 (s, 3H), 7.44 (dd, *J* = 9.0, 2.6 Hz, 1H), 7.59 (ddd, *J* = 8.1, 6.9, 1.2 Hz, 1H), 7.66–7.78 (m, 3H), 7.90 (d, *J* = 8.9 Hz, 1H), 7.95 (d, *J* = 8.9 Hz, 1H), 8.08 (dd, *J* = 8.2, 1.6 Hz, 1H), 8.24 (d, *J* = 2.7 Hz, 1H), 8.55 (dd, *J* = 7.7, 1.8 Hz, 1H), 8.69 (dd, *J* = 8.0, 1.4 Hz, 1H), 9.08 (dd, *J* = 8.7, 4.0 Hz, 2H).^13^C NMR (CDCl_3_, 101 MHz) δ 55.74, 106.60, 112.93, 115.56, 116.86, 120.07, 121.65, 122.51, 122.71, 123.44, 124.54, 125.58, 126.16, 126.75, 127.52, 127.68, 127.81, 128.70, 129.62, 129.94, 130.46, 131.48, 150.12, 154.25, 157.36. HRMS (ESI) *m*/*z* calcd. For C_25_H_17_O_2_ [M + H]^+^: 349.1228, found 349.1259.

#### 13-Chloronaphtho[2,1-*b*]phenanthro[9,10-*d*]furan (**11d**)

White solid. M.p. 231.4–232.1 °C (ethanol).^1^H NMR (CDCl_3_, 400 MHz) δ 7.59 (app t,1 H), 7.66–7.85 (m, 4H), 7.88–7.99 (m, 2H), 8.07 (d, *J* = 7.6 Hz, 1H), 8.54 (d, *J* = 7.6 Hz, 1H), 8.67 (d, *J* = 8.0 Hz, 1H), 8.76 (s, 1H), 8.99 (d, *J* = 8.0 Hz, 1H), 9.05 (d, *J* = 8.8 Hz, 1H). ^13^C NMR (CDCl_3_, 101 MHz) δ 100.12, 105.98, 112.95, 116.57, 119.88, 121.78, 122.74, 123.53, 123.95, 124.82, 125.48, 126.45, 127.01, 127.41, 127.69, 128.12, 128.30, 128.57, 129.57, 129.81, 130.29, 131.31, 131.62, 151.31, 154.44. HRMS (ESI) *m*/*z* calcd. for C_24_H_14_ClO [M + H]^+^: 353.0728, found 353.0752.

#### Chryseno[6,5-b]naphtho[1,2-*d*]furan (**11e**)

White solid. M.p. 212.1–213.6 °C (taken-up with petroleum ether/DCM). ^1^H NMR (CDCl_3_, 400 MHz) δ 7.28–7.40 (m, 2H), 7.48 (ddd, *J* = 8.1, 6.8, 1.2 Hz, 1H), 7.62 (ddd, *J* = 8.0, 6.8, 1.2 Hz, 1H), 7.70–7.82 (m, 3H), 7.95–8.15 (m, 5H), 8.42 (d, *J* = 8.3 Hz, 1H), 8.60–8.73 (m, 1H), 8.74–8.85 (m, 2H). ^13^C NMR (CDCl_3_, 101 MHz) δ 112.79, 114.96, 120.66, 121.48, 121.65, 121.94, 123.69, 124.41, 124.74, 124.92, 125.83, 126.31, 126.74, 127.11, 127.14, 127.31, 127.61, 128.25, 128.52, 128.60, 129.01, 129.90, 130.09, 130.51, 131.22, 132.63, 152.38, 154.20. HRMS (ESI) *m*/*z* calcd. for C_28_H_17_O [M + H]^+^: 369.1279, found 369.1299.

#### Naphtho[2,1-*b*]thieno[2’,3’:3,4]naphtho[2,1-*d*]furan (**11f**)

White solid. M.p. 200.2–201.3 °C (taken-up with petroleum ether/DCM). ^1^H NMR (CDCl_3_, 400 MHz) δ 7.55–7.73 (m, 4H), 7.80 (t, *J* = 7.7 Hz, 1H), 7.91 (d, *J* = 8.9 Hz, 1H), 7.96 (d, *J* = 8.9 Hz, 1H), 8.06 (d, *J* = 8.1 Hz, 1H), 8.16 (d, *J* = 5.5 Hz, 1H), 8.39–8.48 (m, 1H), 8.55–8.63 (m, 1H), 9.47 (d, *J* = 8.4 Hz, 1H). ^13^C NMR (CDCl_3_, 101 MHz) δ 112.94, 115.65, 118.96, 120.24, 121.76, 122.92, 122.98, 124.24, 124.67, 125.57, 126.09, 126.67, 126.84, 128.17, 128.25, 128.39, 129.53, 130.78, 131.17, 133.49, 150.39, 153.88. HRMS (ESI) *m*/*z* calcd. for C_22_H_13_OS [M + H]^+^: 325.0687, found 325.0698.

#### 2-(2-(Methylthio)phenyl)-1-(*p*-tolyl)-1,2-dihydronaphtho[2,1-*b*]furan (**12a**)

White solid. M.p. 75.2–76.1 °C (ethanol). ^1^H NMR (DMSO, 400 MHz) δ 7.93–7.86 (2 part. overlapped d, 2H), 7.47–7.19 (m, 9H), 7.15–7.08 (m, 3H), 5.96 (d, *J* = 4.4 Hz, 1H), 4.80 (d, *J* = 4.4 Hz, 1H), 2.47 (s, 3H), 2.25 (s, 3H). ^13^C NMR (DMSO, 101 MHz) δ 15.54, 20.54, 54.53, 89.40, 111.85, 121.18, 122.41, 122.92, 124.80, 124.84, 126.22, 126.70, 127.36, 128.49, 128.65, 129.13, 129.49, 129.84, 130.24, 135.79, 135.87, 138.29, 140.12, 156.50. HRMS (ESI) *m*/*z* calcd. for C_26_H_23_OS [M + H]^+^: 383.1470, found 383.1498.

#### 2-(2-(Methylthio)phenyl)-1-(*p*-tolyl)naphtho[2,1-*b*]furan (**13a**)

White solid. M.p. 92.5–93.6 °C (ethanol). ^1^H NMR (CDCl_3_, 400 MHz) δ 2.42 (s, 6H), 7.06 (td, *J* = 8.00 and 4.00 Hz), 7.20–7.45 (m, 10H), 7.77 (AB system, *J* = 8.8 Hz), 7.90 (d, *J* = 8.8 Hz), 7.95 (d, *J* = 8.0 Hz). ^13^C NMR (CDCl_3_, 101 MHz) δ 16.23, 21.62, 112.73, 121.81, 122.47, 123.55, 124.40, 124.49, 125.75, 126.01, 126.03, 128.64, 129.10, 129.42, 129.62, 129.67, 130.63, 130.67, 131.09, 131.95, 137.41, 140.58, 150.96, 152.21. HRMS (ESI) *m*/*z* calcd. for C_26_H_21_OS [M + H]^+^: 381.1313, found 383.1320.

The described NMR spectra plots of all synthesized compounds are collected in the Supplementary Materials.

### 3.7. Crystal Structure of **5a**

A single crystal of compound **5a** was submitted to X-ray data collection on an Oxford-Diffraction Xcalibur Sapphire 3 diffractometer with a graphite monochromated Mo-Kα radiation (λ = 0.71073 Å) at 293 K. The structure was solved by direct methods implemented in SHELXS-97 program [45]. The refinement was carried out by full-matrix anisotropic least-squares on F2 for all reflections for non-H atoms by means of the SHELXL-97 program (Version 2019/2) [46]. Crystallographic data for this structure have been deposited with the Cambridge Crystallographic Data Centre as supplementary publication no. CCDC 2166047. Copies of the data can be obtained, free of charge, on application to CCDC, 12 Union Road, Cambridge CB2 1EZ, UK; (Fax: +44-(0)-1223-336-033; or e-mail: deposit@ccdc.cam.ac.uk).

## Figures and Tables

**Table 1:** Results obtained in the reactions of sulfones **4a–f** with 2-naphthol/K_2_CO_3_ in DMSO at rt.

4	Ar in 4	5 (Yields %) ^a^
**4a**	*p*-MeC_6_H_4_	**5a**, 92
**4b**	*o*-MeC_6_H_4_	**5b**, 89
**4c**	*p*-MeOC_6_H_4_	**5c**, 94
**4d**	*p*-ClC_6_H_4_	**5d**, 93
**4e**	1-Naphthyl	**5e**, 87
**4f**	2-Thienyl	**5f**, 96

^a^ Yields refer to products isolated by filtration from the final reaction mixture.

## Data Availability

Not applicable.

## Supplementary Materials

The following supporting information can be downloaded at: https://www.mdpi.com/article/10.3390/molecules27103147/s1, ^1^H and ^13^C NMR spectra for all compounds.

## Sample Availability

Samples of all of the compounds are available from the authors.
