# Supplementary material for: An Easy Access to Furan-Fused Polyheterocyclic Systems
Source: Molecules. 2022 May 14;27(10):3147. doi: 10.3390/molecules27103147 (PMC9143548; doi:10.3390/molecules27103147)

Supplementary Materials

An Easy Access to Furan Fused Polyheterocyclic Systems

Alice Benzi[1], Lara Bianchi[2], Gianluca Giorgi[2], Massimo Maccagno[1], Giovanni Petrillo[1], Domenico Spinelli[3] and Cinzia Tavani[1,]*

[1] Dipartimento di Chimica e Chimica Industriale, Università degli Studi di Genova, Via Dodecaneso 31, 16146 Genova, Italy; cinzia.tavani@unige.it (C.T.); massimo.maccagno@unige.it (M.M.); lara.bianchi@unige.it (L.B.); giovanni.petrillo@unige.it
[2] Dipartimento di Biotecnologie, Chimica e Farmacia, Università di Siena, Via A. Moro, 53100 Siena, Italy; gianluca.giorgi@unisi.it
[3] Dipartimento di Chimica "G. Ciamician", Alma Mater Studiorum-University of Bologna, via F. Selmi 2, 40126 Bologna, Italy; domenico.spinelli@unibo.it

* Correspondence: cinzia.tavani@unige.it

Table of Contents

^{1}H and ^{13}C NMR spectra for all compounds S2-29

IGDS1_ff11-16_RP_EP_CM
single_pulse

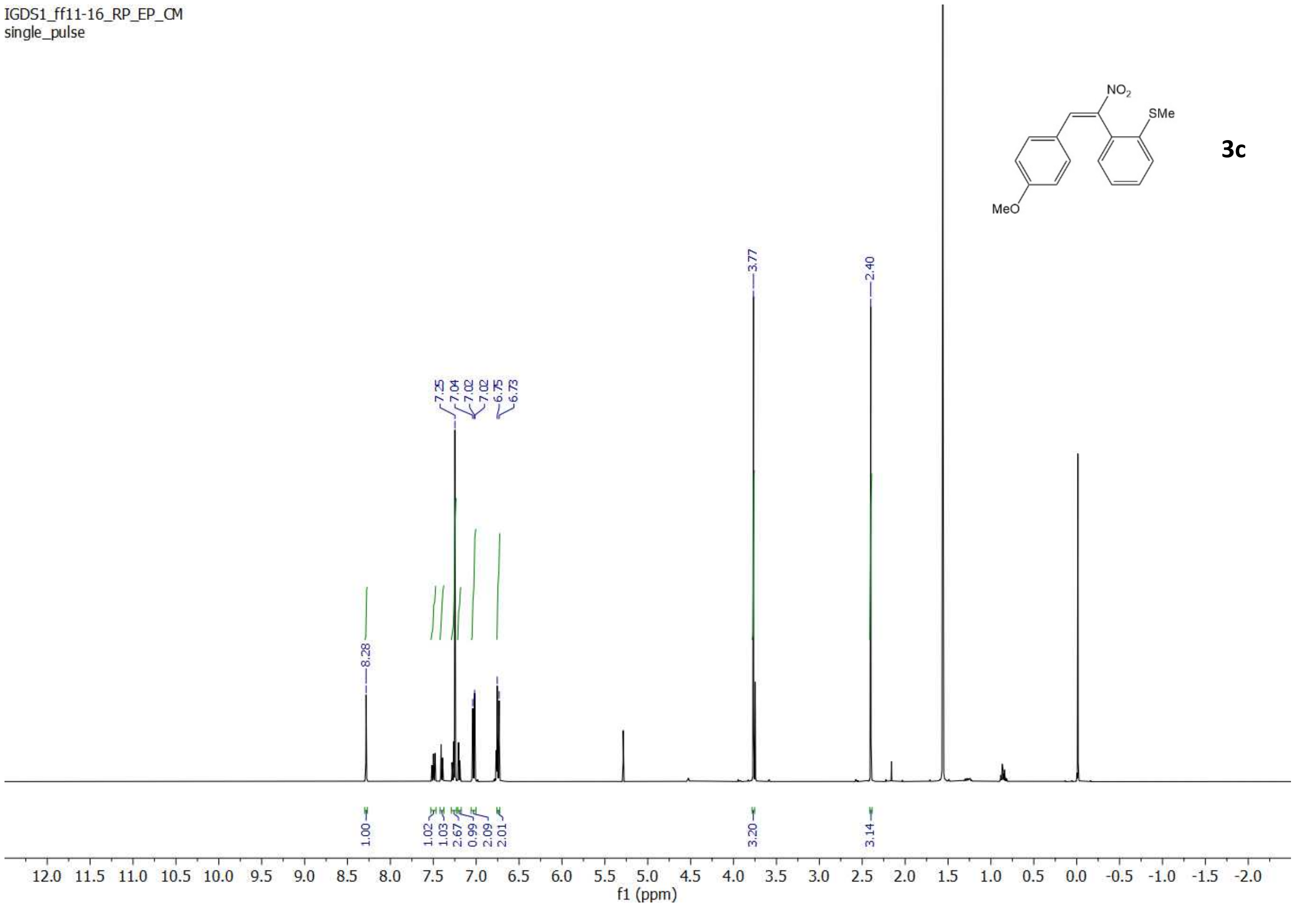
3c

IISAS6_ripreso
single pulse decoupled gated NOE

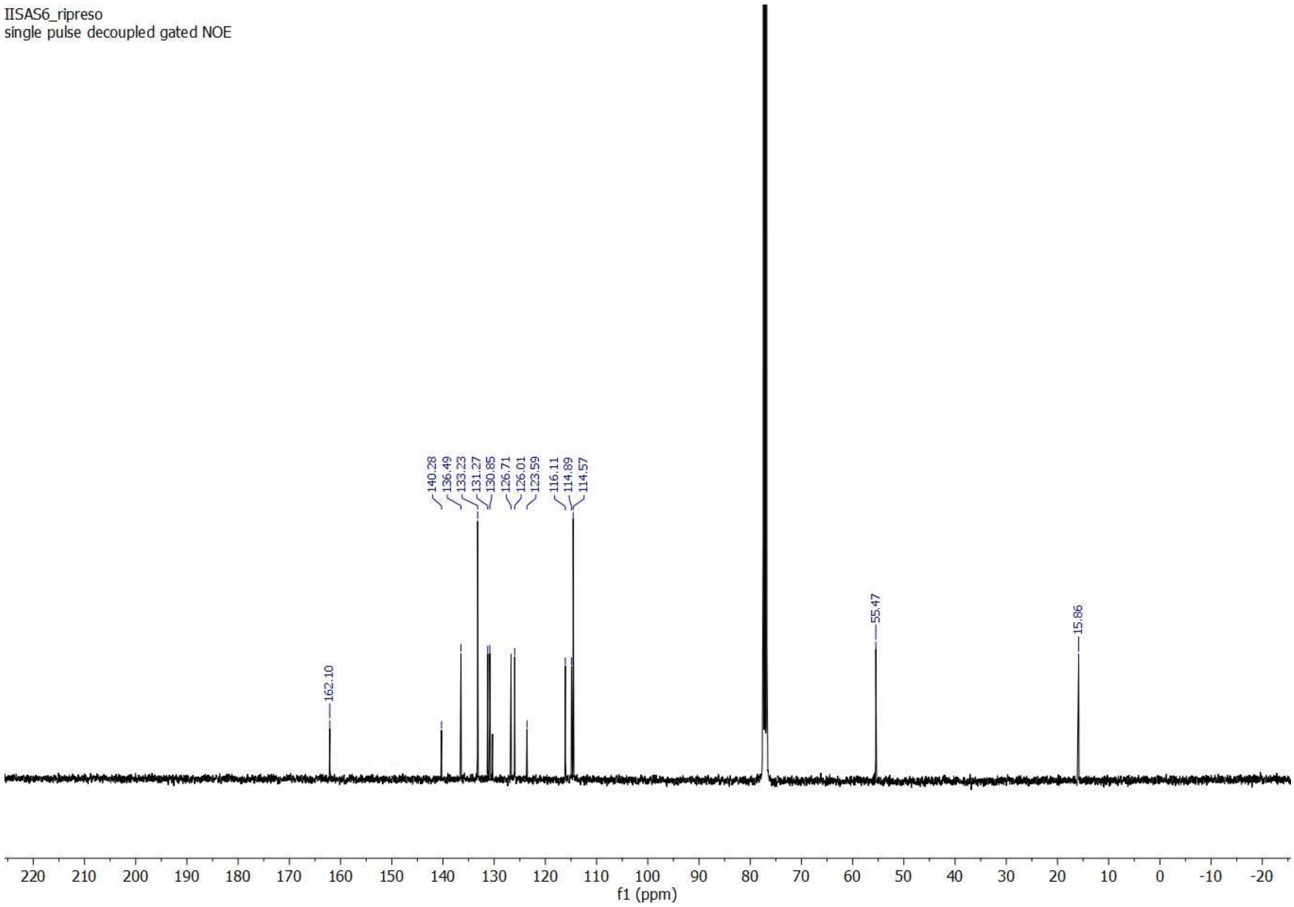

IGDS2_grezzo
single_pulse

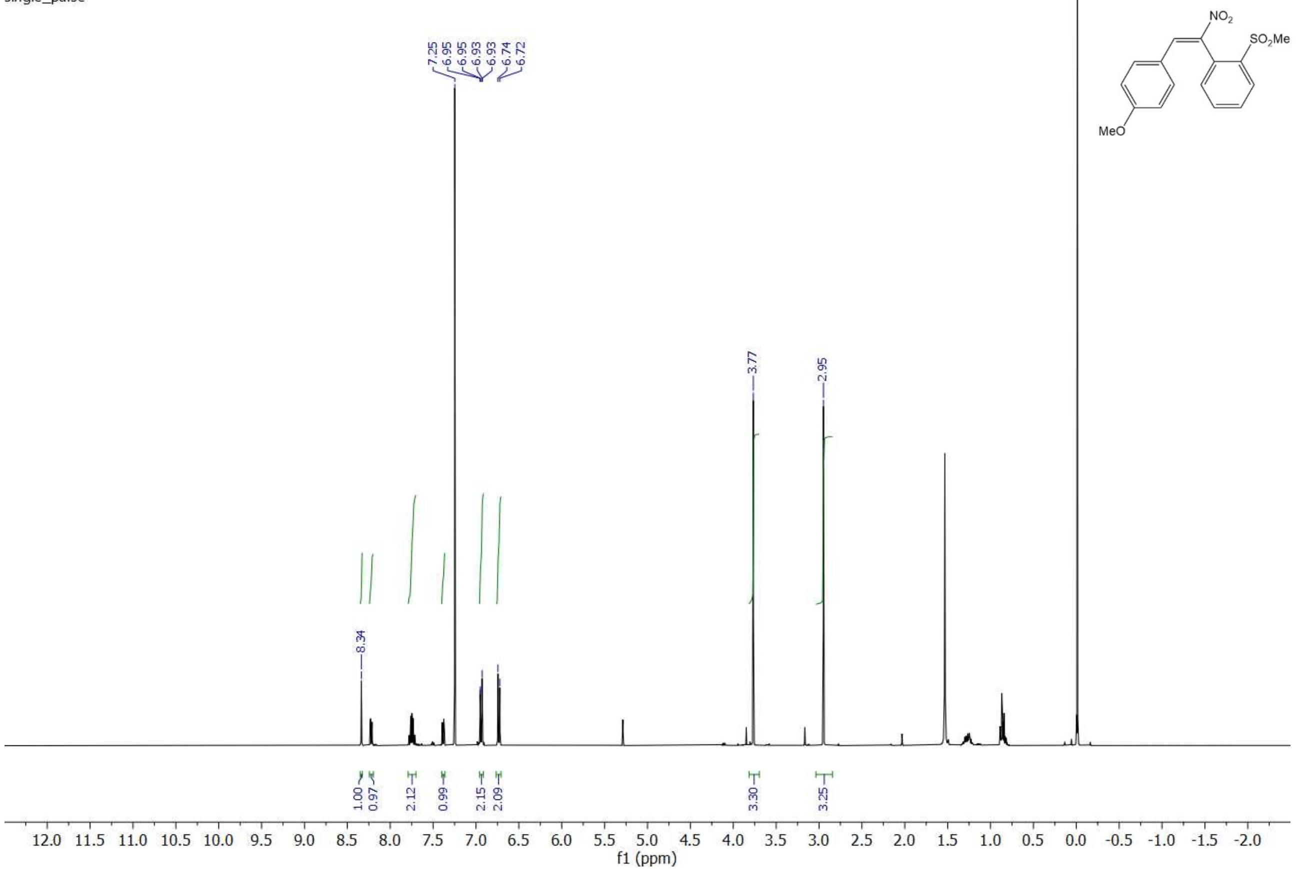

4c

IGDS2
single pulse decoupled gated NOE

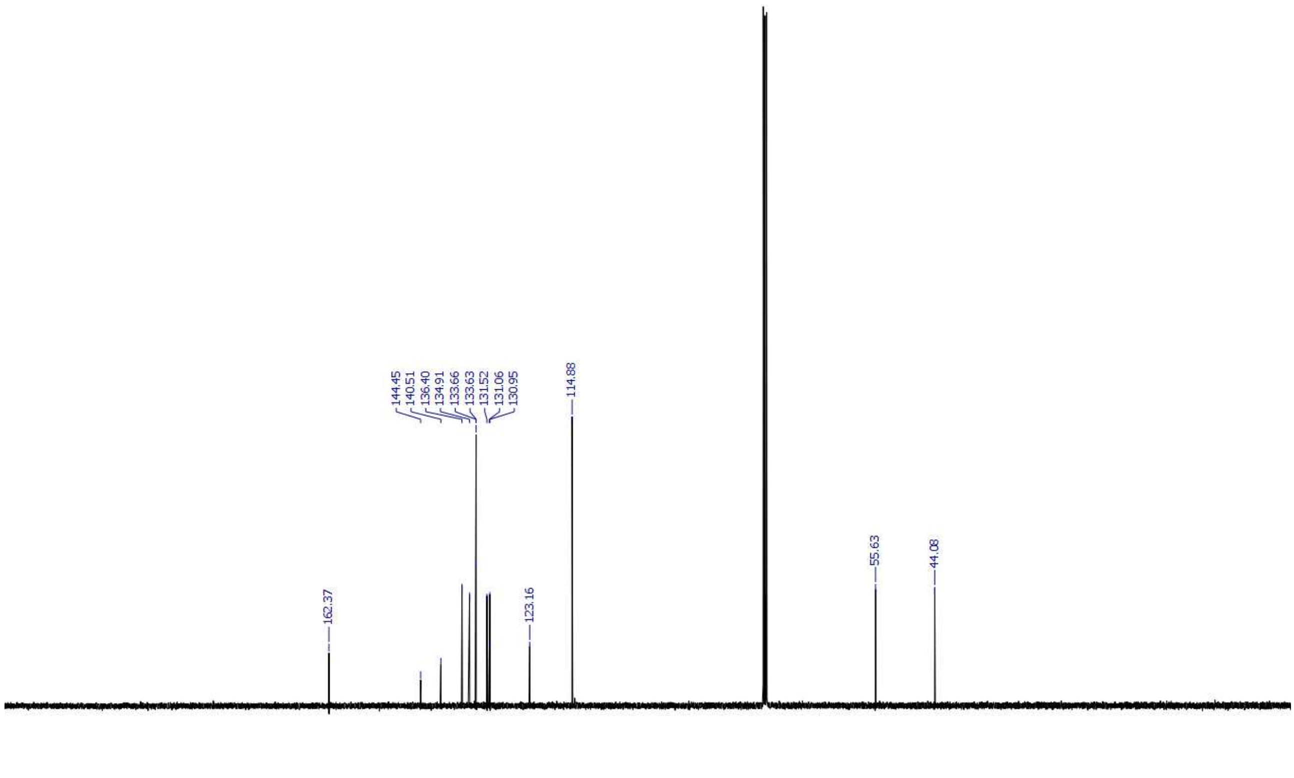

VBNZ68_ripEP

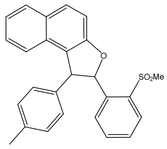

5a

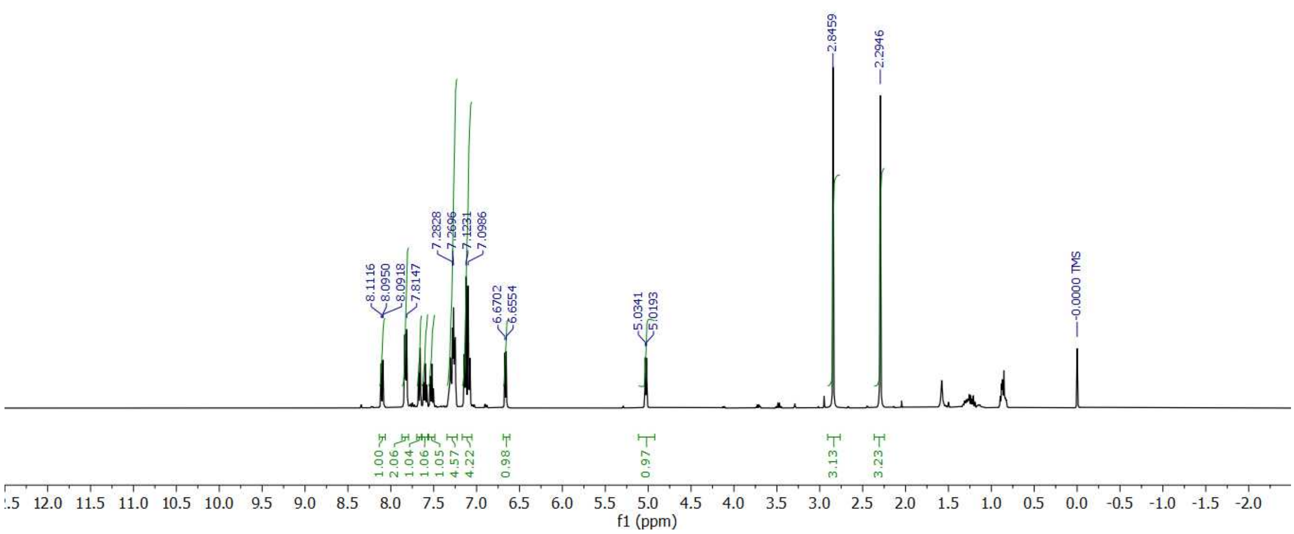

VBNZ68_ripEP

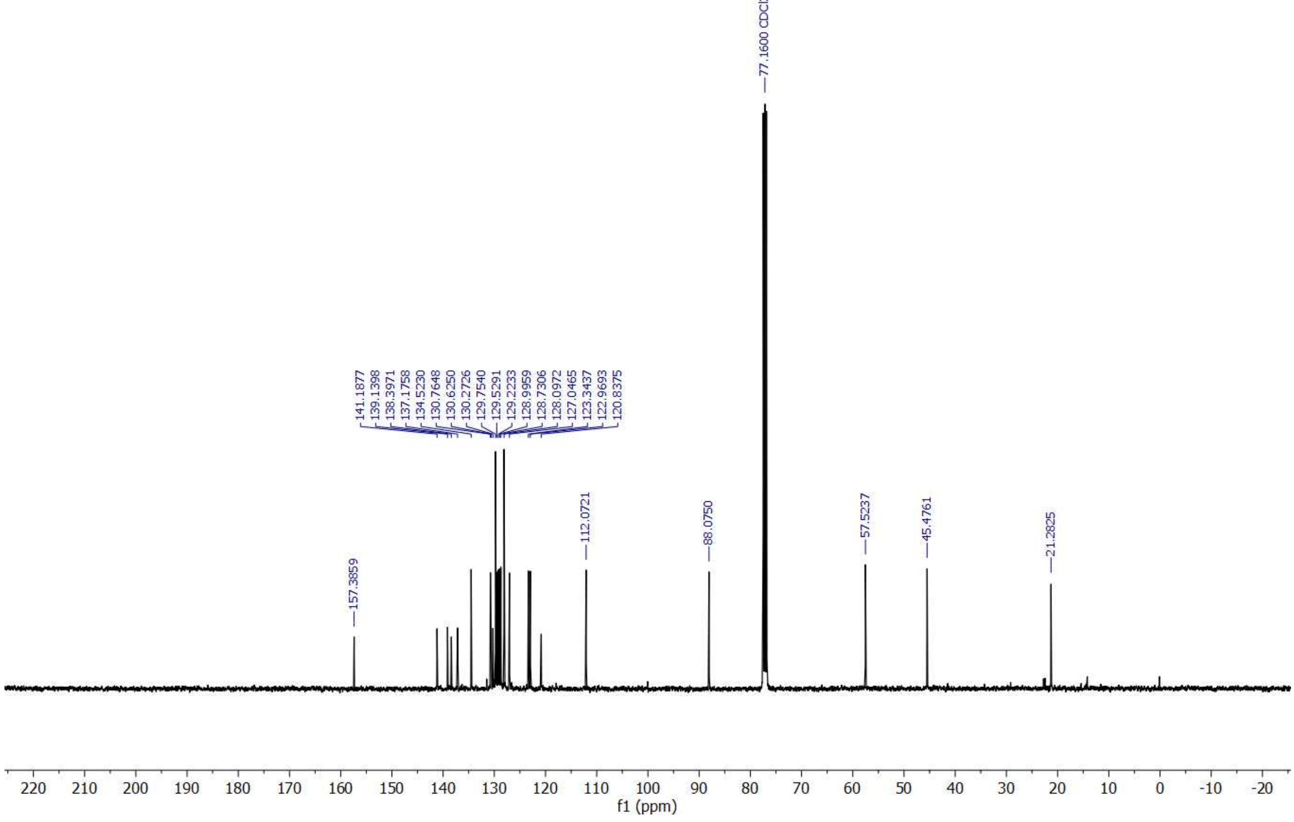

DNF_o-tol_naftolo_rpEPCM

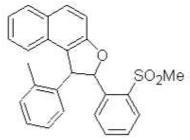

5b

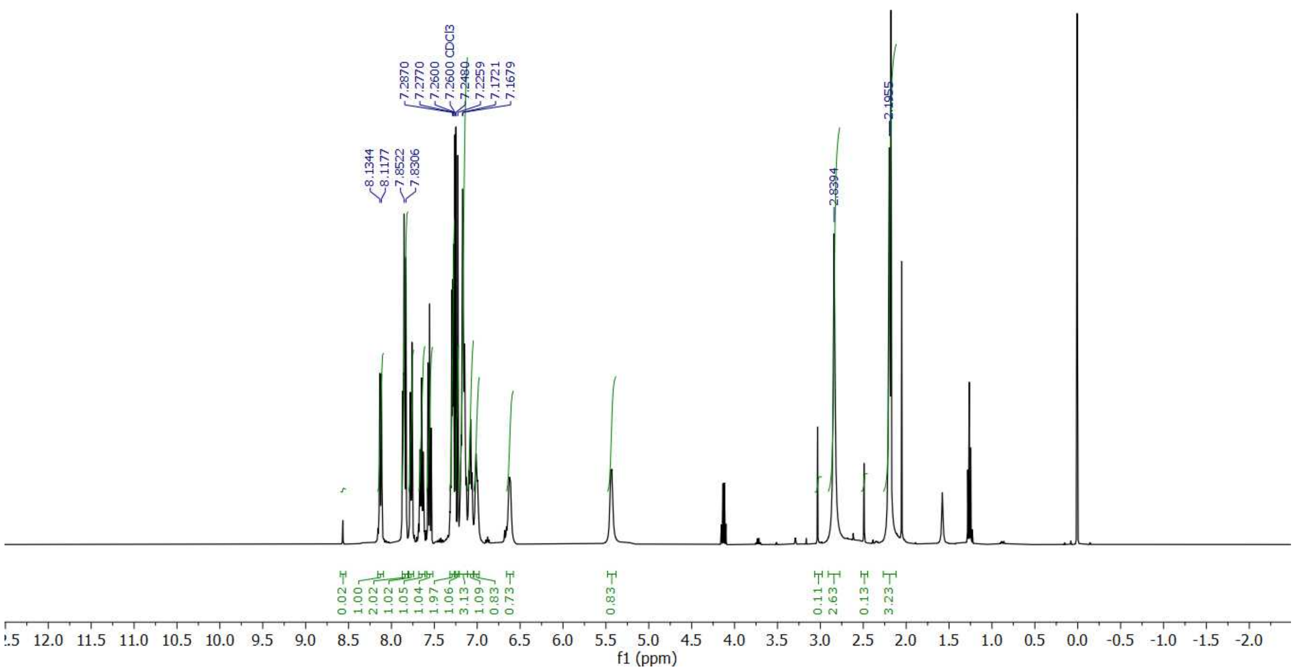

DNF_o-tol_naftolo_rpEPCM

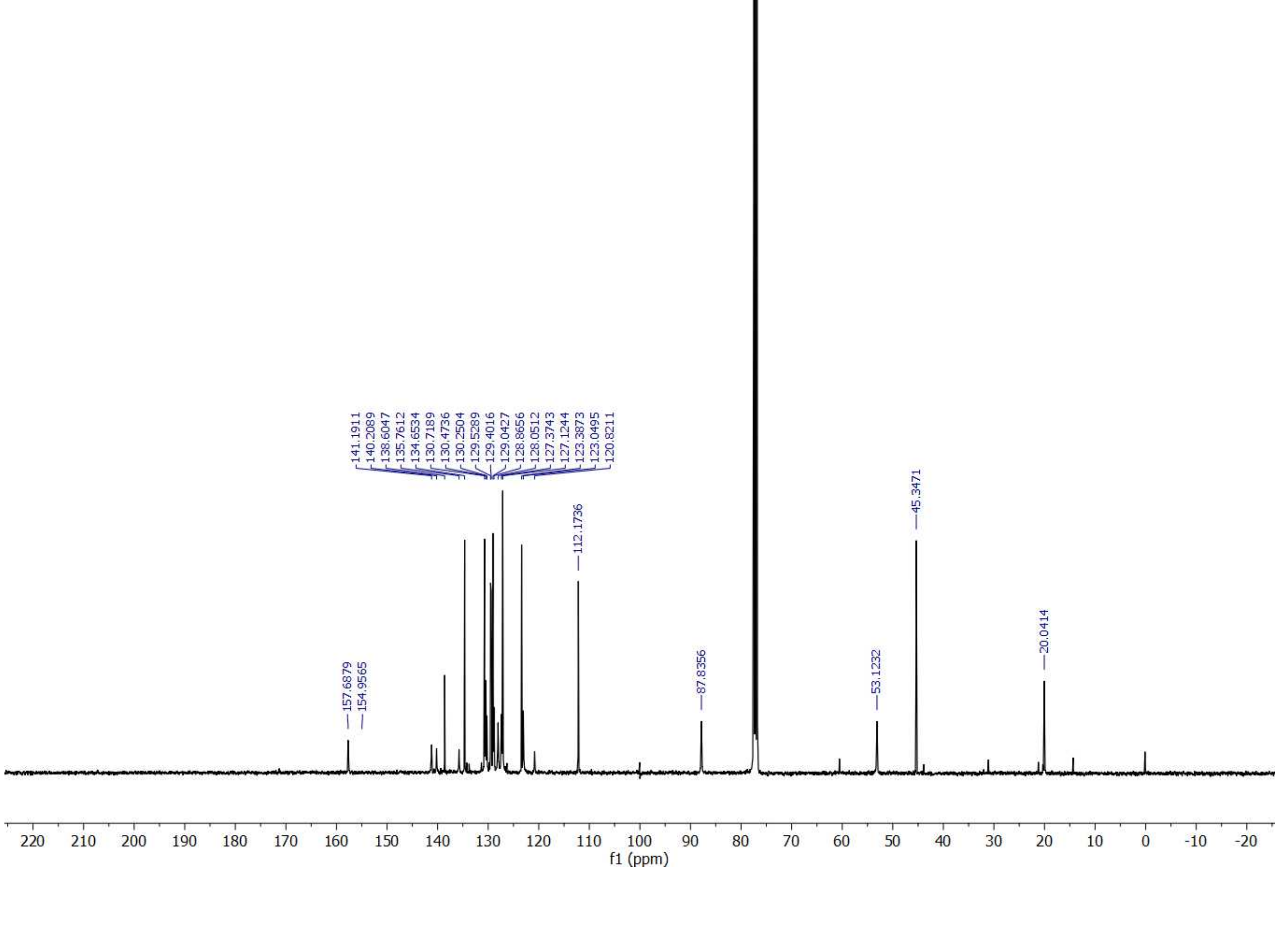

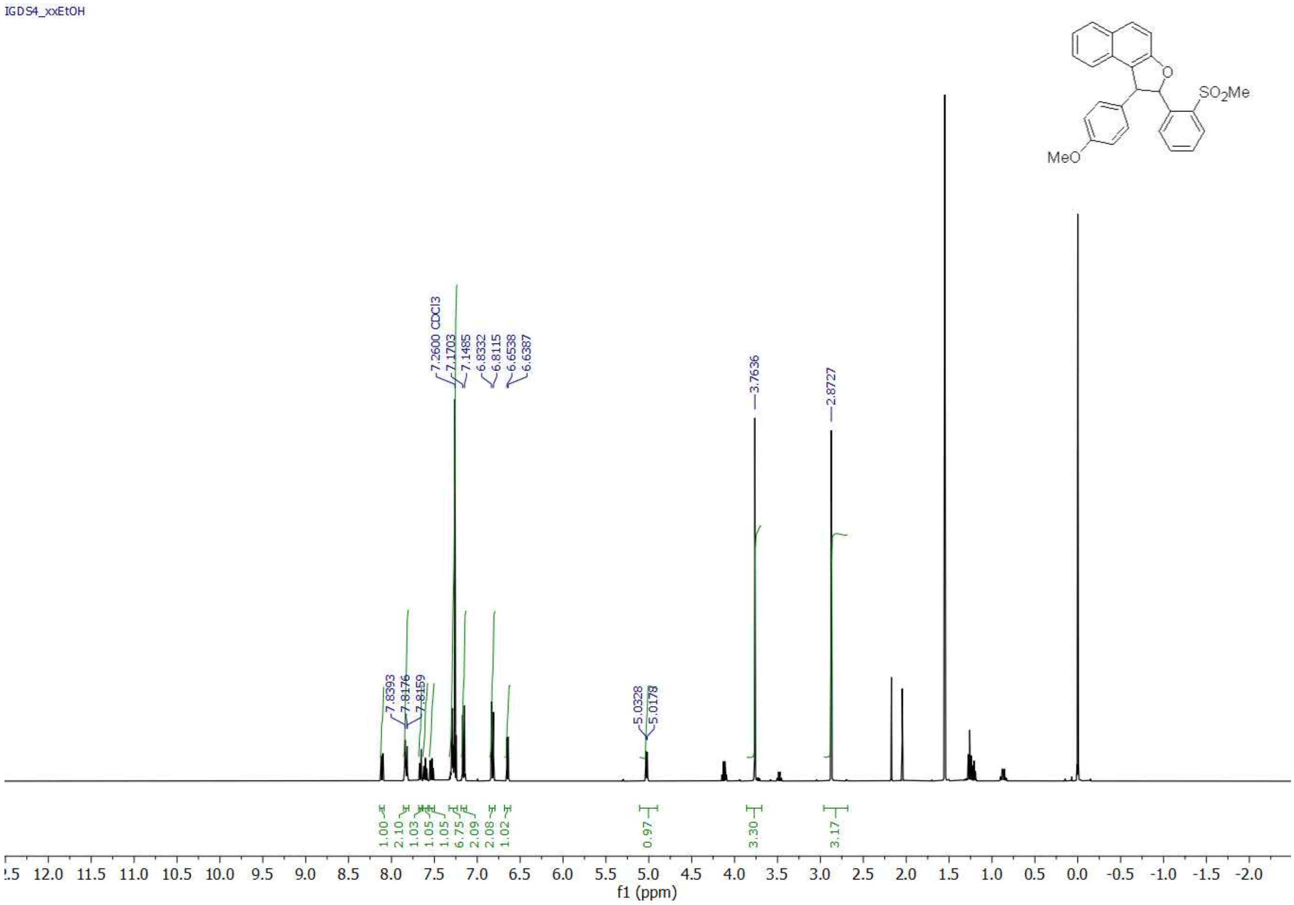
IGDS4_xxEtOH
SO2Me
MeO
O
7.2600 CDCl3
7.1703
7.1485
6.8332
6.8115
6.6538
6.6387
3.7636
2.8727
7.8393
7.8176
7.8159
5.0328
5.0178
1.00
2.10
1.03
1.05
1.05
6.75
2.09
2.08
1.02
0.97
3.30
3.17
f1 (ppm)

5c

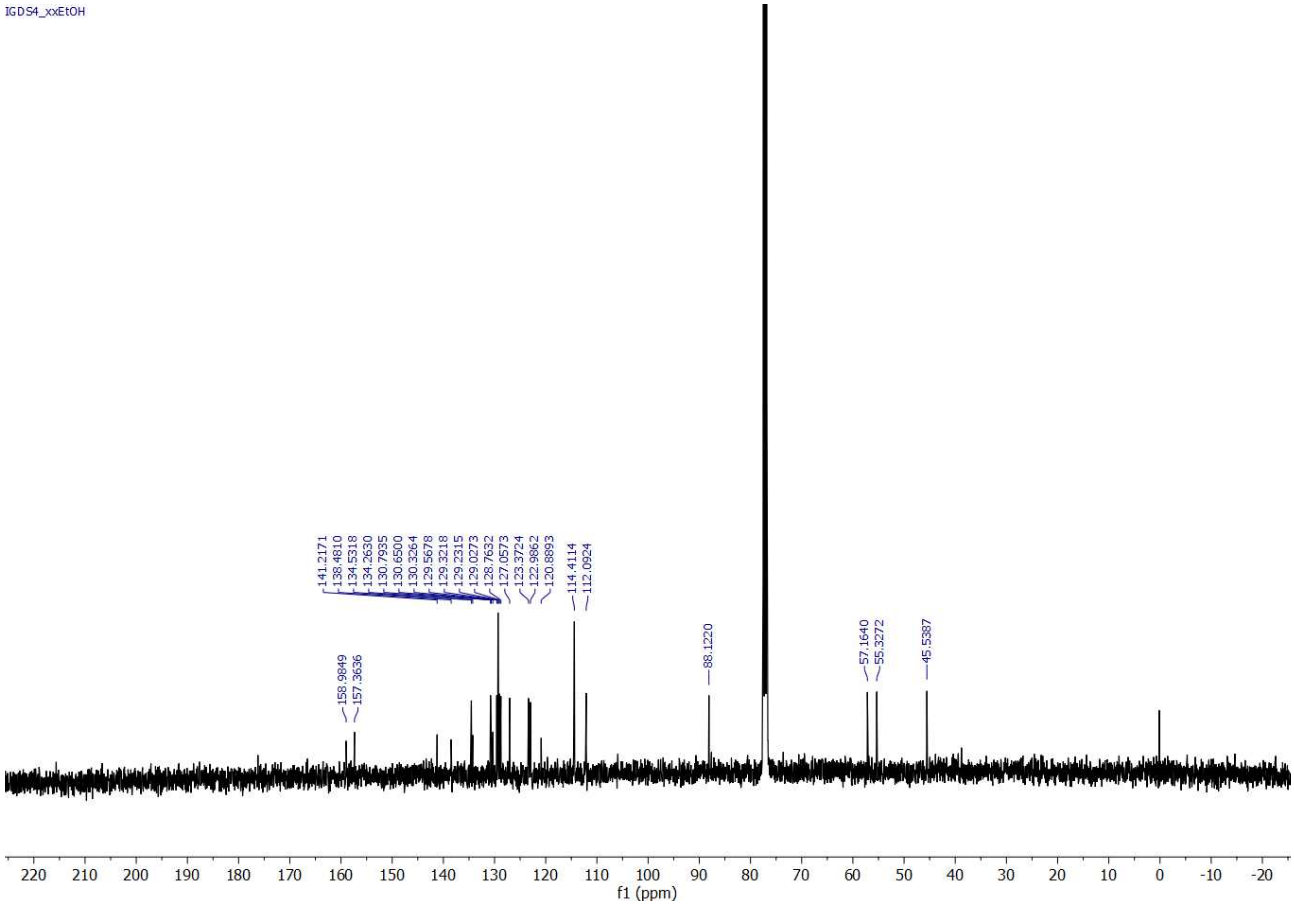
IGDS4_xxEtOH
141.2171
138.4810
134.5318
134.2630
130.7935
130.6500
130.3264
129.5678
129.3218
129.2315
129.0273
128.7632
127.0573
123.3724
122.9862
120.8893
114.4114
112.0924
158.9849
157.3636
88.1220
57.1640
55.3272
45.5387
f1 (ppm)

VIBNZ13_grezzo

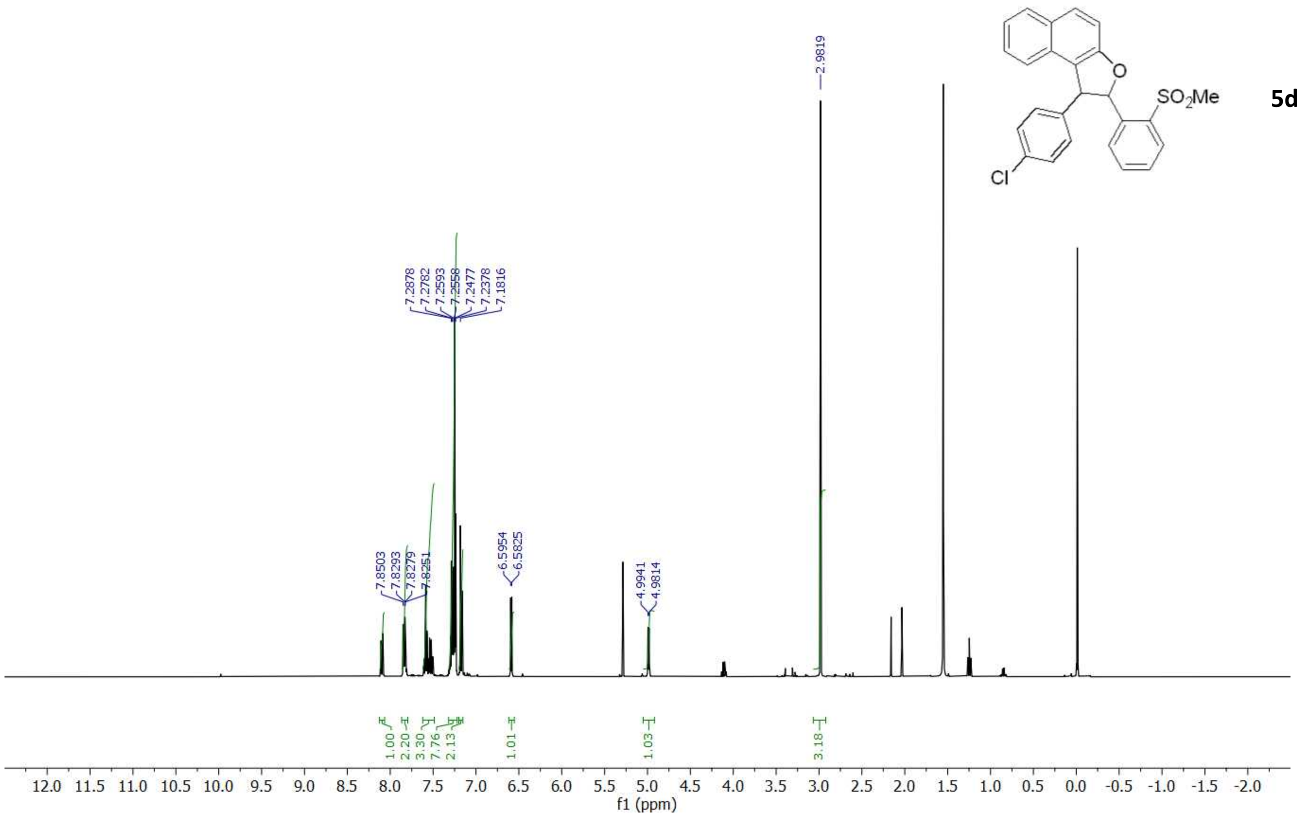

VBNZ77_grezzo

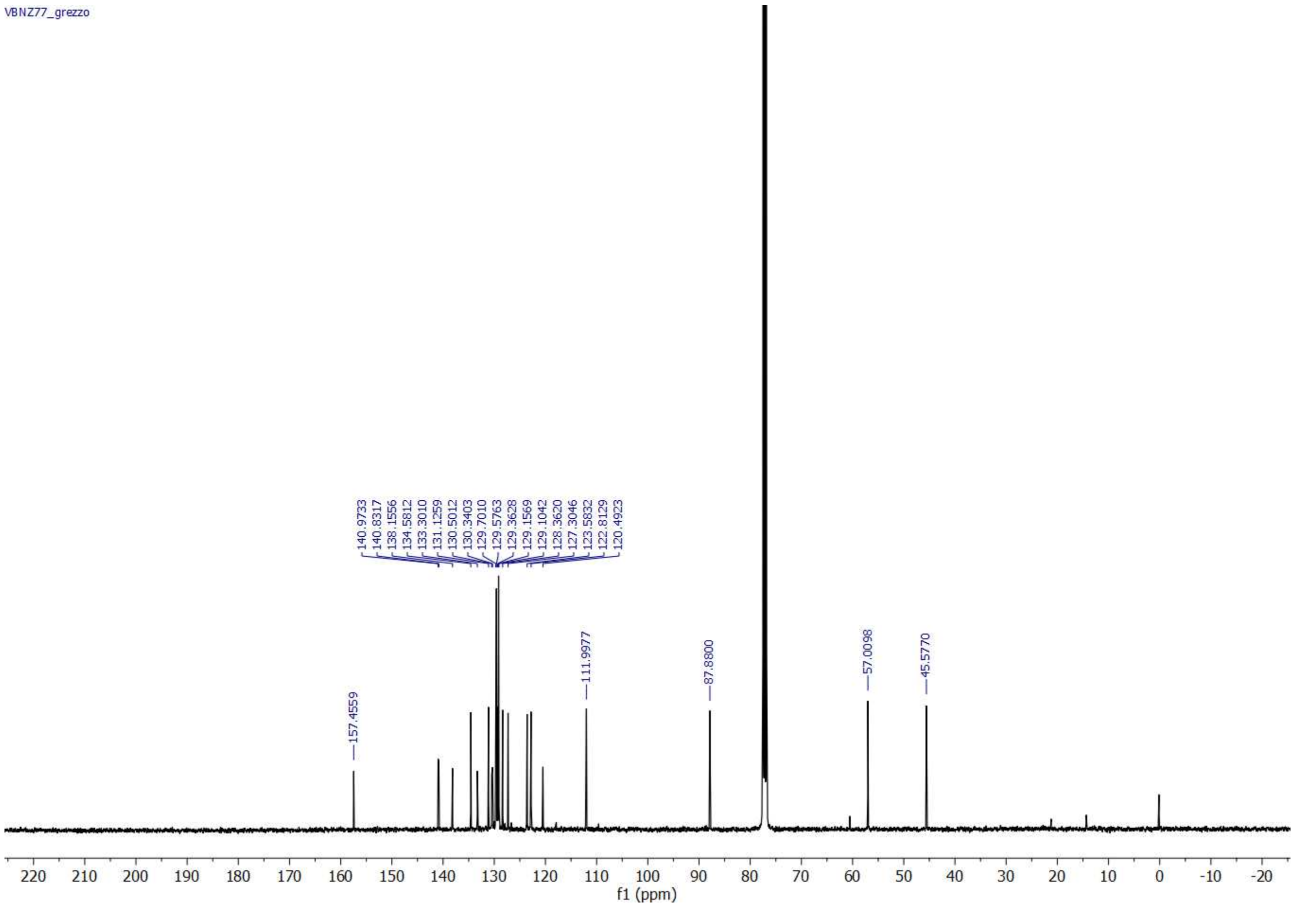

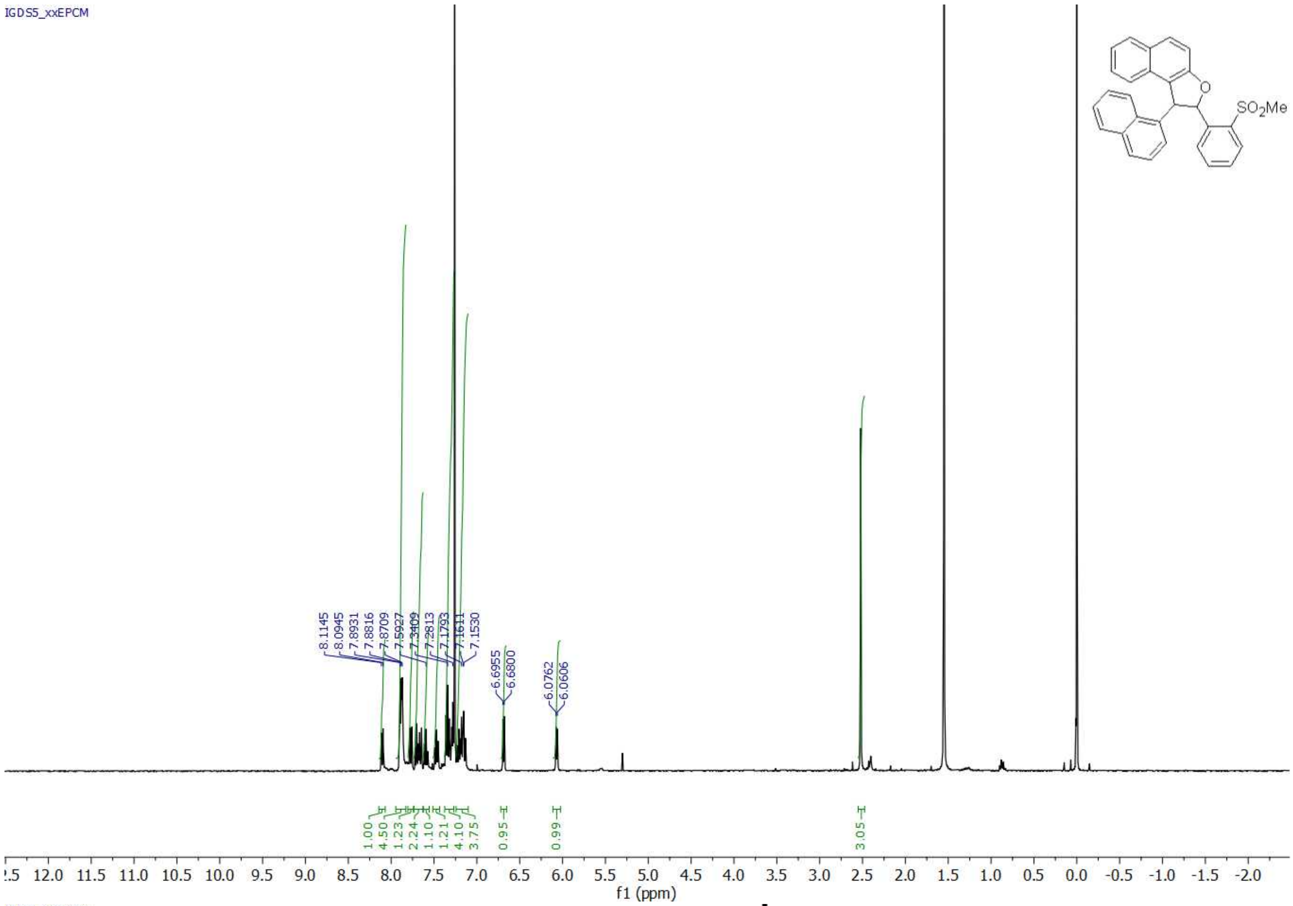
IGDS5_xxEPCM
8.1145
8.0945
7.8931
7.8816
7.8709
7.5927
7.3409
7.2813
7.1793
7.1611
7.1530
6.6955
6.6800
6.0762
6.0606
1.00
4.50
1.23
2.24
1.10
1.21
4.10
3.75
0.95
0.99
3.05
12.0 11.5 11.0 10.5 10.0 9.5 9.0 8.5 8.0 7.5 7.0 6.5 6.0 5.5 5.0 4.5 4.0 3.5 3.0 2.5 2.0 1.5 1.0 0.5 0.0 -0.5 -1.0 -1.5 -2.0
f1 (ppm)
O
SO2Me

5e

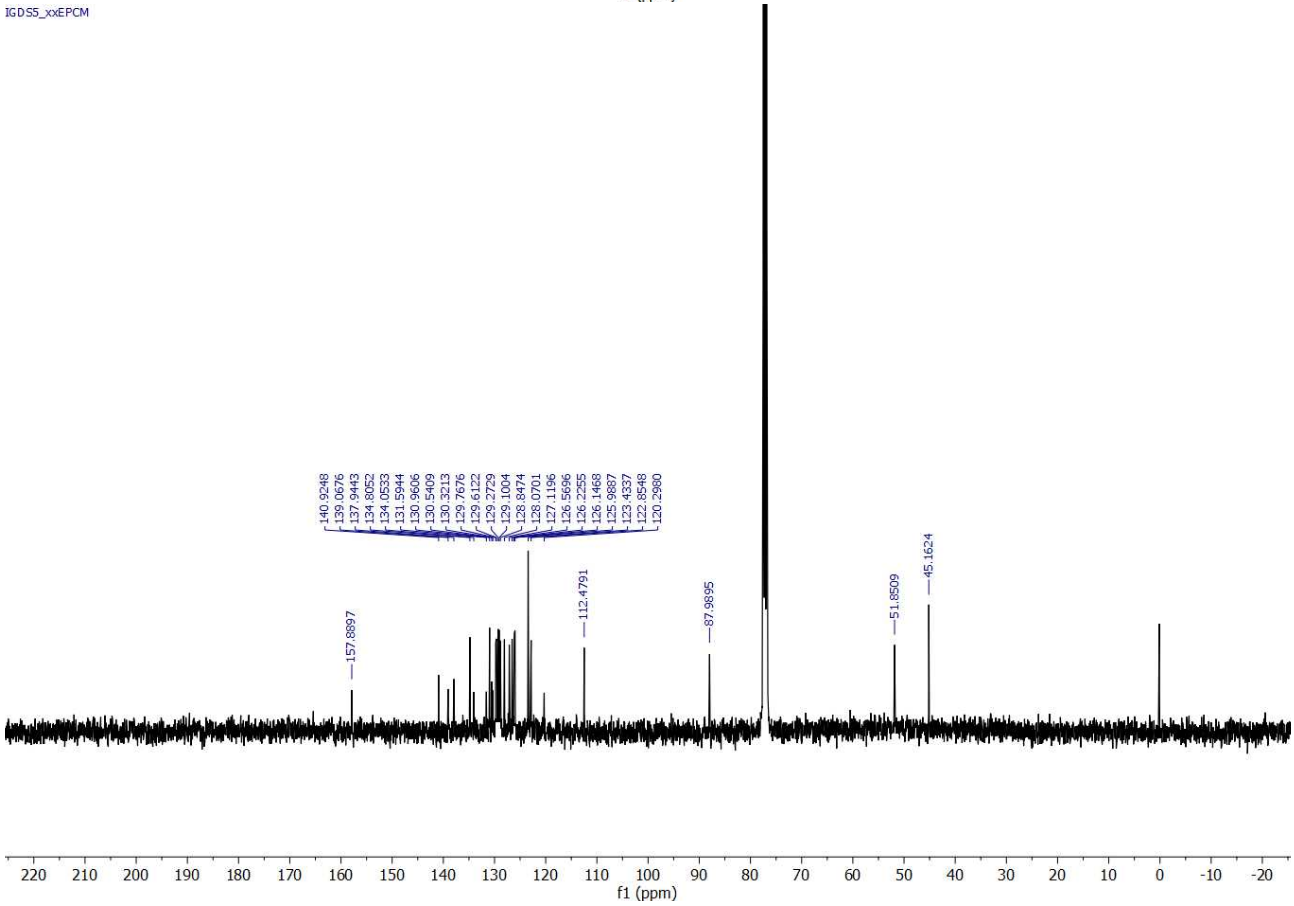
IGDS5_xxEPCM
140.9248
139.0676
137.9443
134.8052
134.0533
131.5944
130.9606
130.5409
130.3213
129.7676
129.6122
129.2729
129.1004
128.8474
128.0701
127.1196
126.5696
126.2255
126.1468
125.9887
123.4337
122.8548
120.2980
157.8897
112.4791
87.9895
51.8509
45.1624
220 210 200 190 180 170 160 150 140 130 120 110 100 90 80 70 60 50 40 30 20 10 0 -10 -20
f1 (ppm)

DNF_2-Th_IGDS14

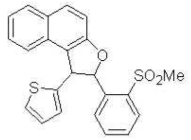

5f

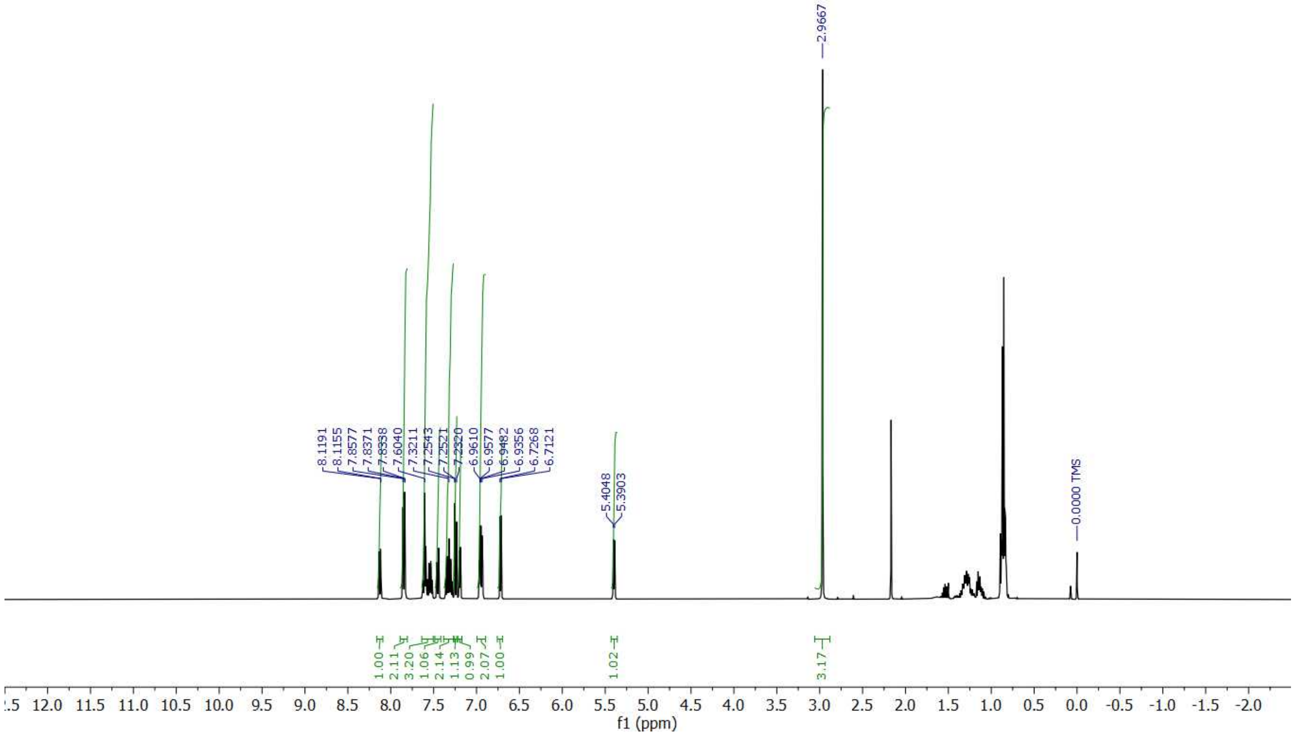

DNF_2-Th_IGDS14

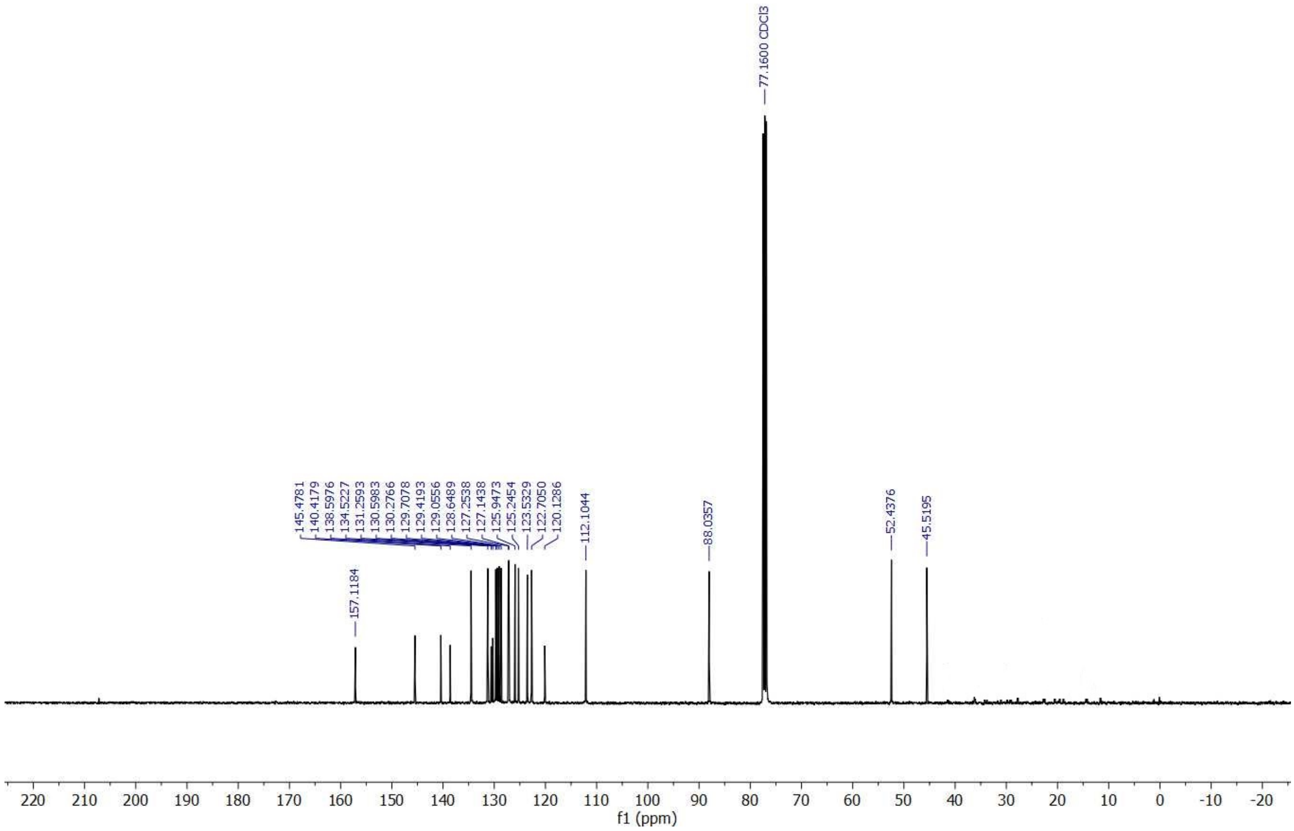

VBNZ71_xxEtOH

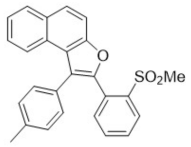

6a

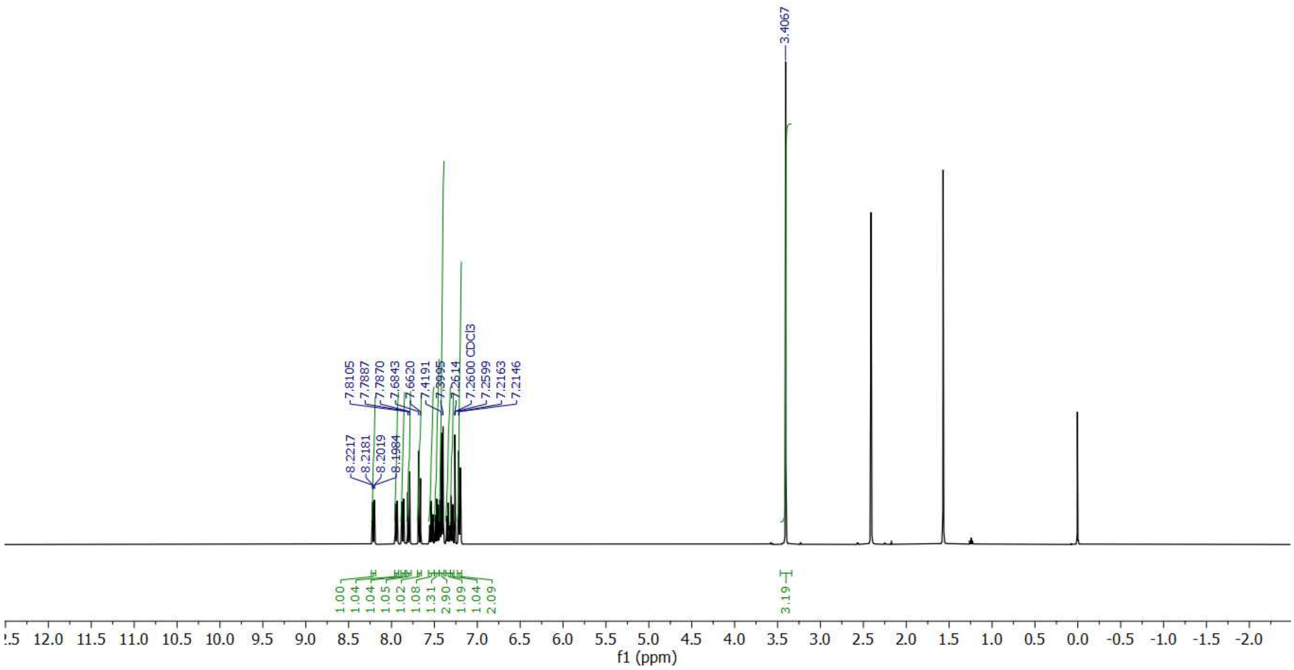

VBNZ71_xxEtOH

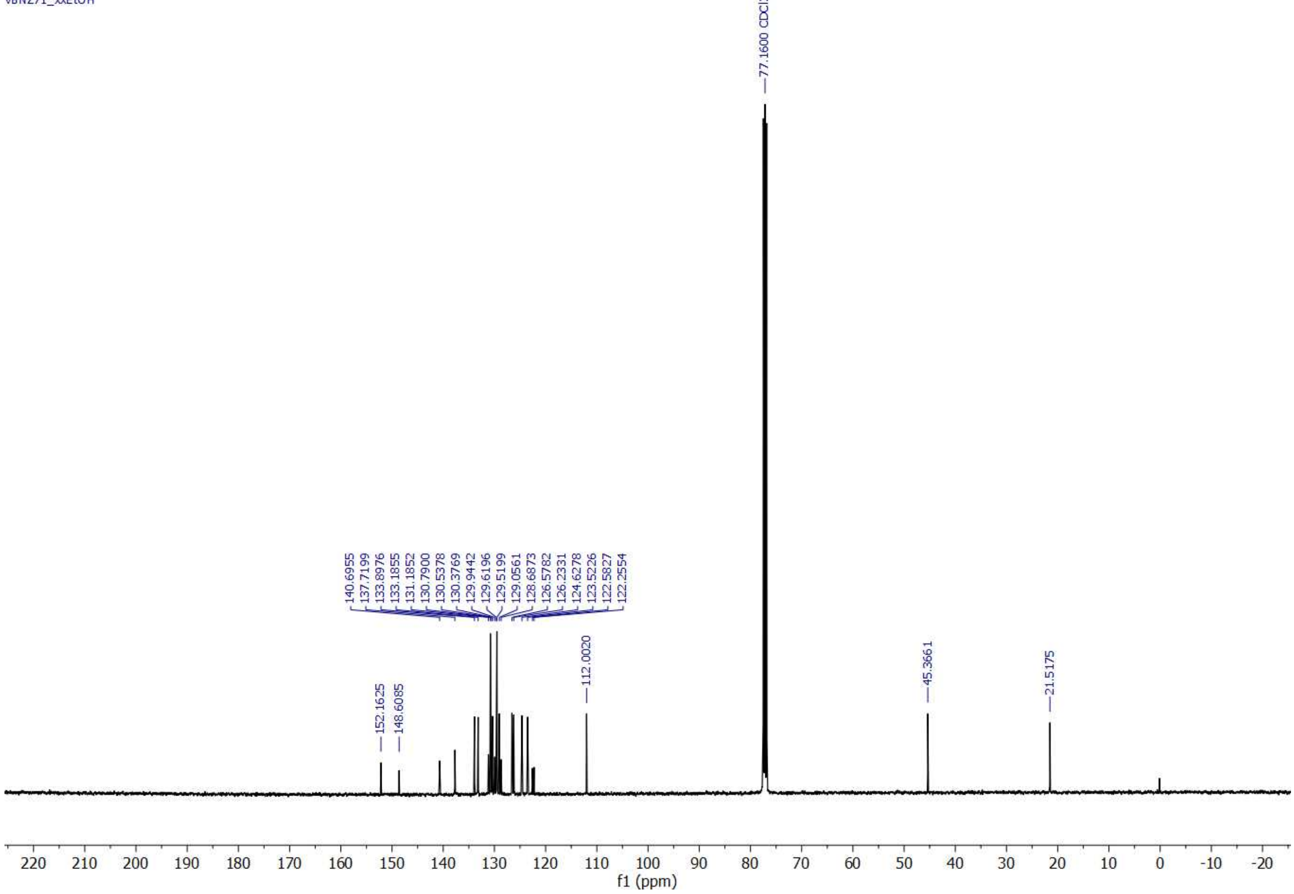

NF_o-tol_naftolo_VIBNZ16

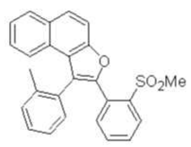

6b

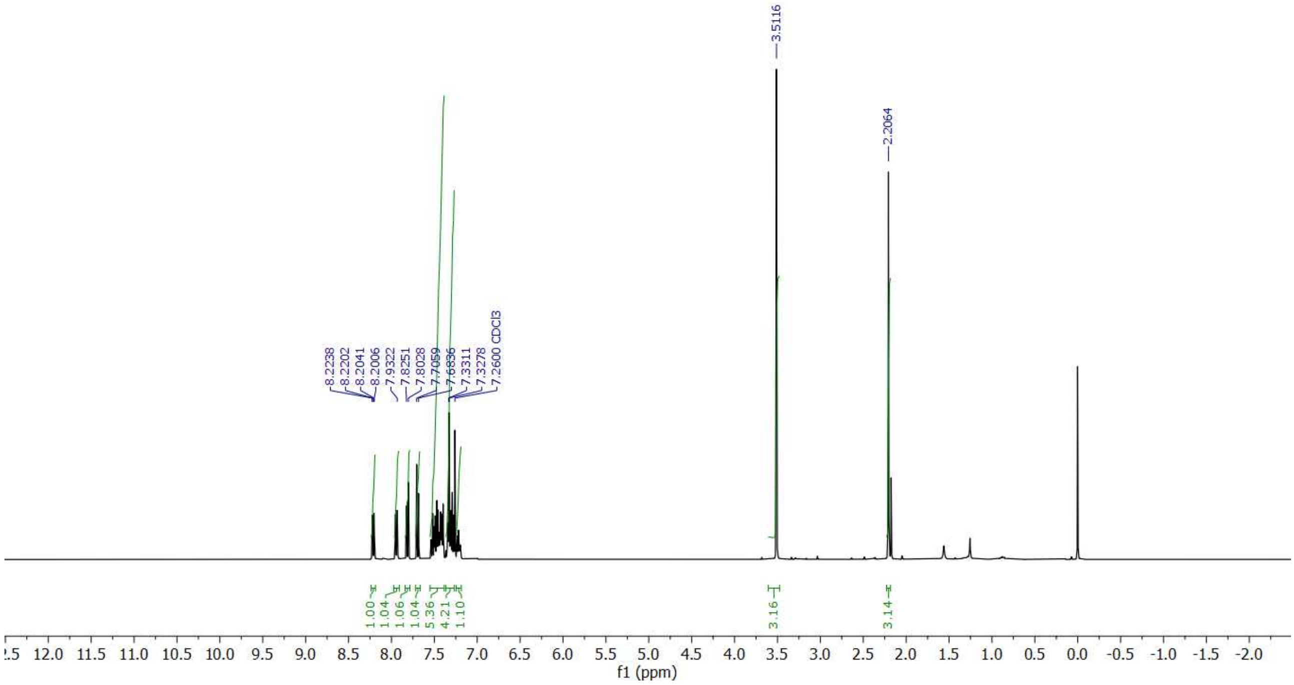

NF_o-tol_naftolo_VIBNZ16

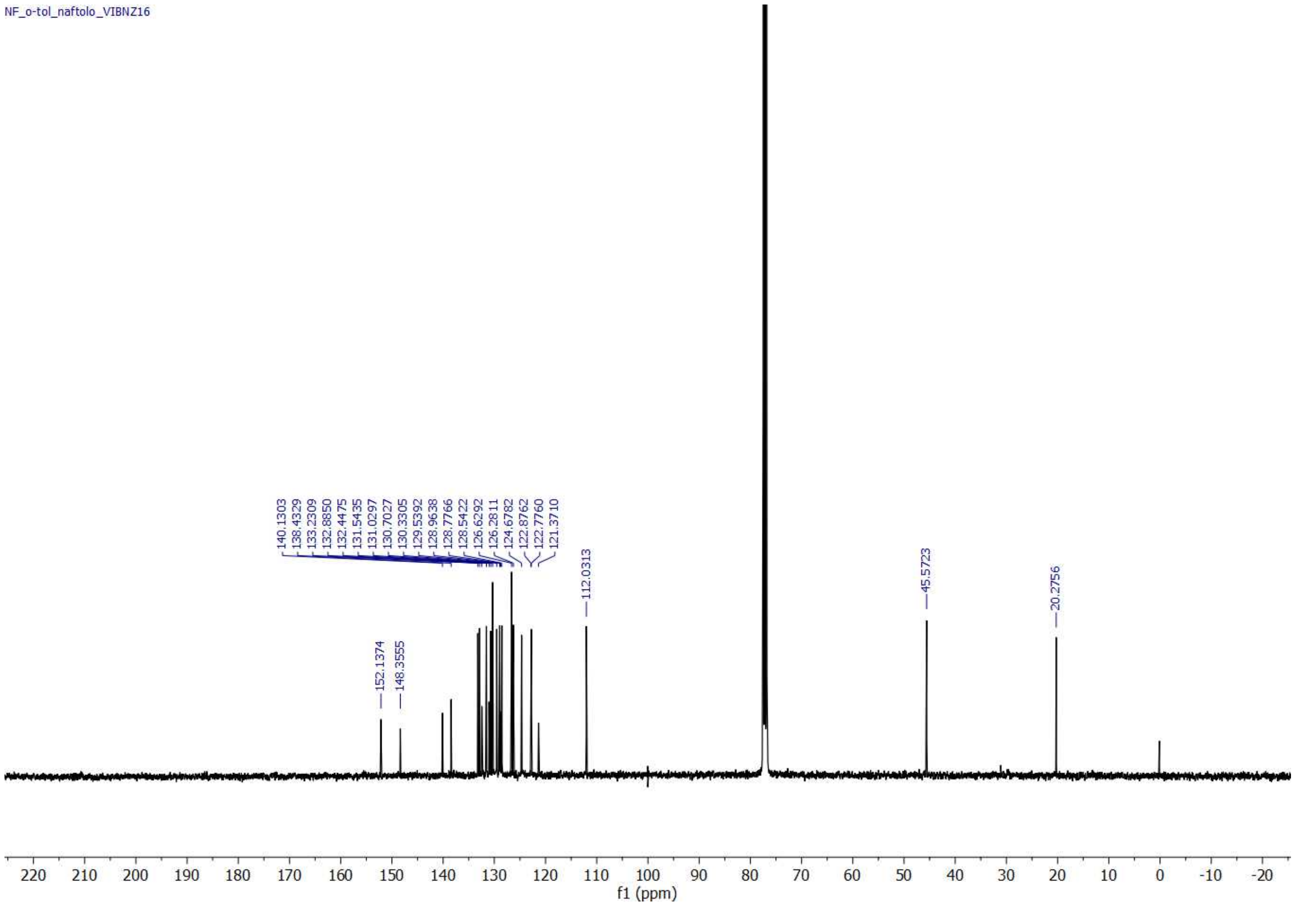

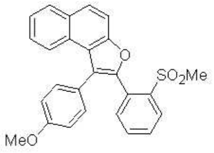
SO2Me
MeO

6c

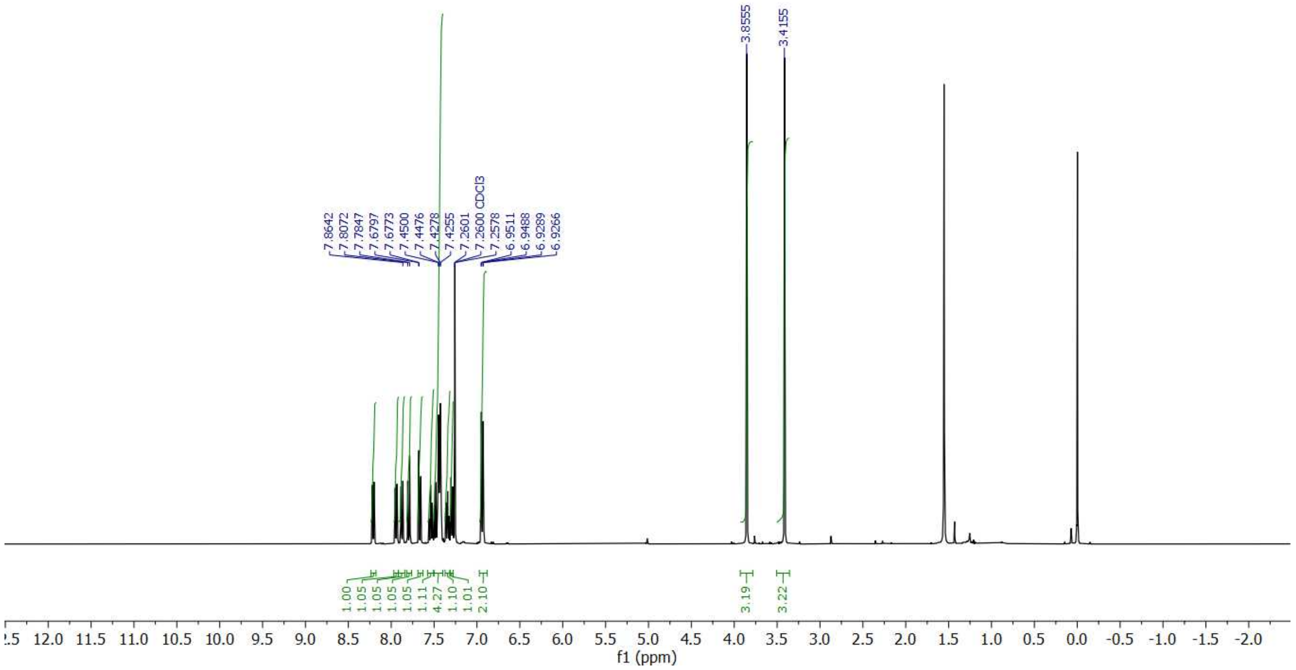
IGDS7grezzo
7.8642
7.8072
7.7847
7.6797
7.6773
7.4500
7.4476
7.4278
7.4255
7.2601
7.2600 CDCl3
7.2578
6.9511
6.9488
6.9289
6.9266
3.8555
3.4155
1.00
1.05
1.05
1.05
1.11
4.27
1.10
1.01
2.10
3.19
3.22
f1 (ppm)

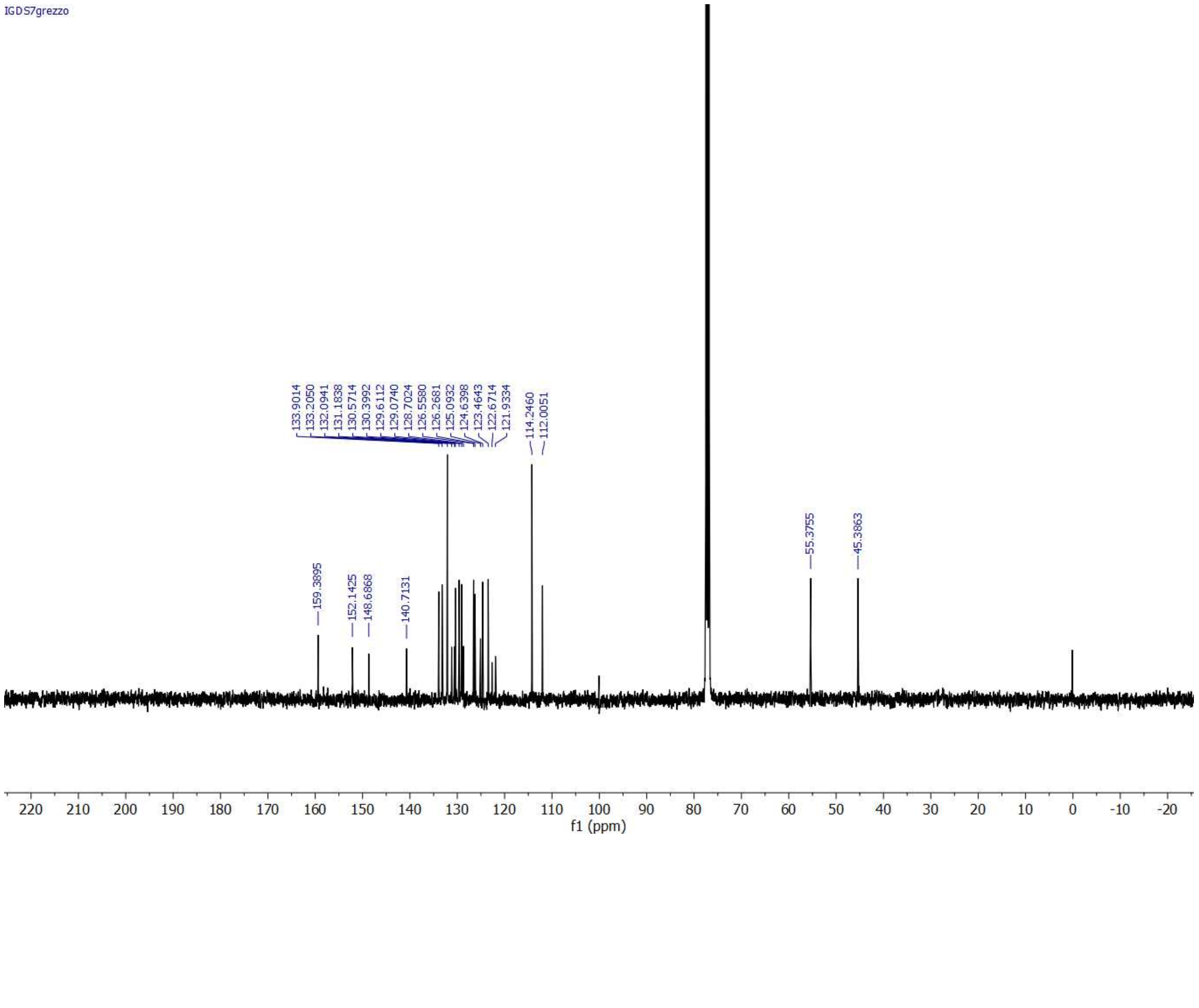
IGDS7grezzo
133.9014
133.2050
132.0941
131.1838
130.5714
130.3992
129.6112
129.0740
128.7024
126.5580
126.2681
125.0932
124.6398
123.4643
122.6714
121.9334
114.2460
112.0051
159.3895
152.1425
148.6868
140.7131
55.3755
45.3863
f1 (ppm)

VIBNZ15_3-10

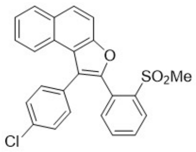

6d

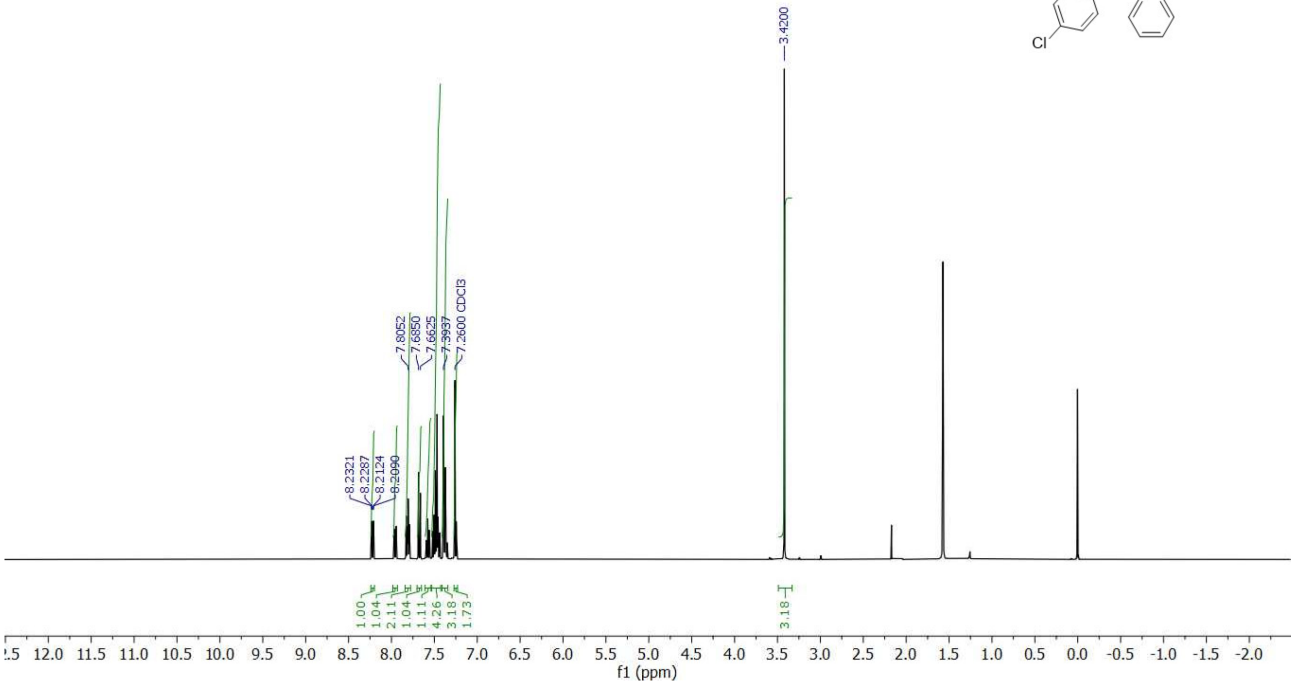

VIBNZ15_3-10

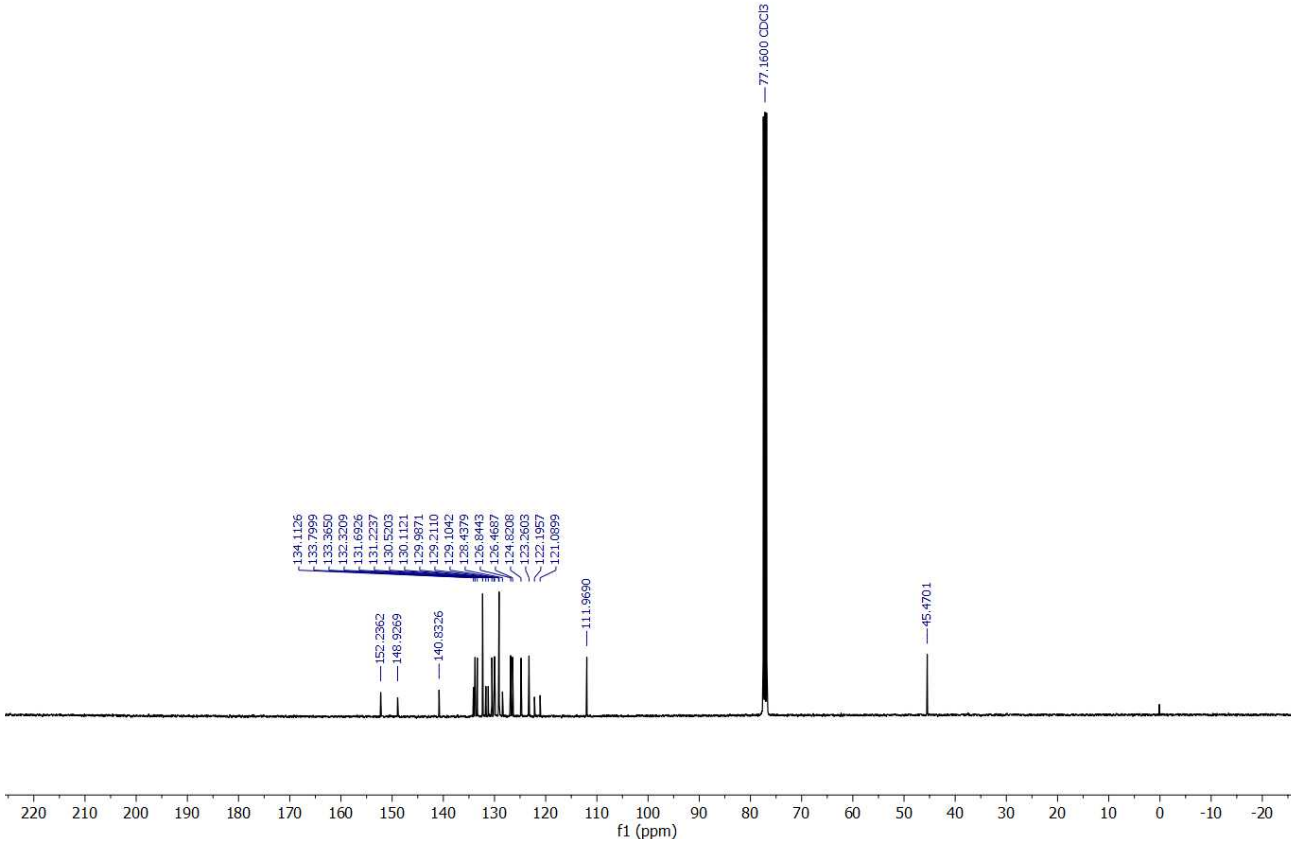

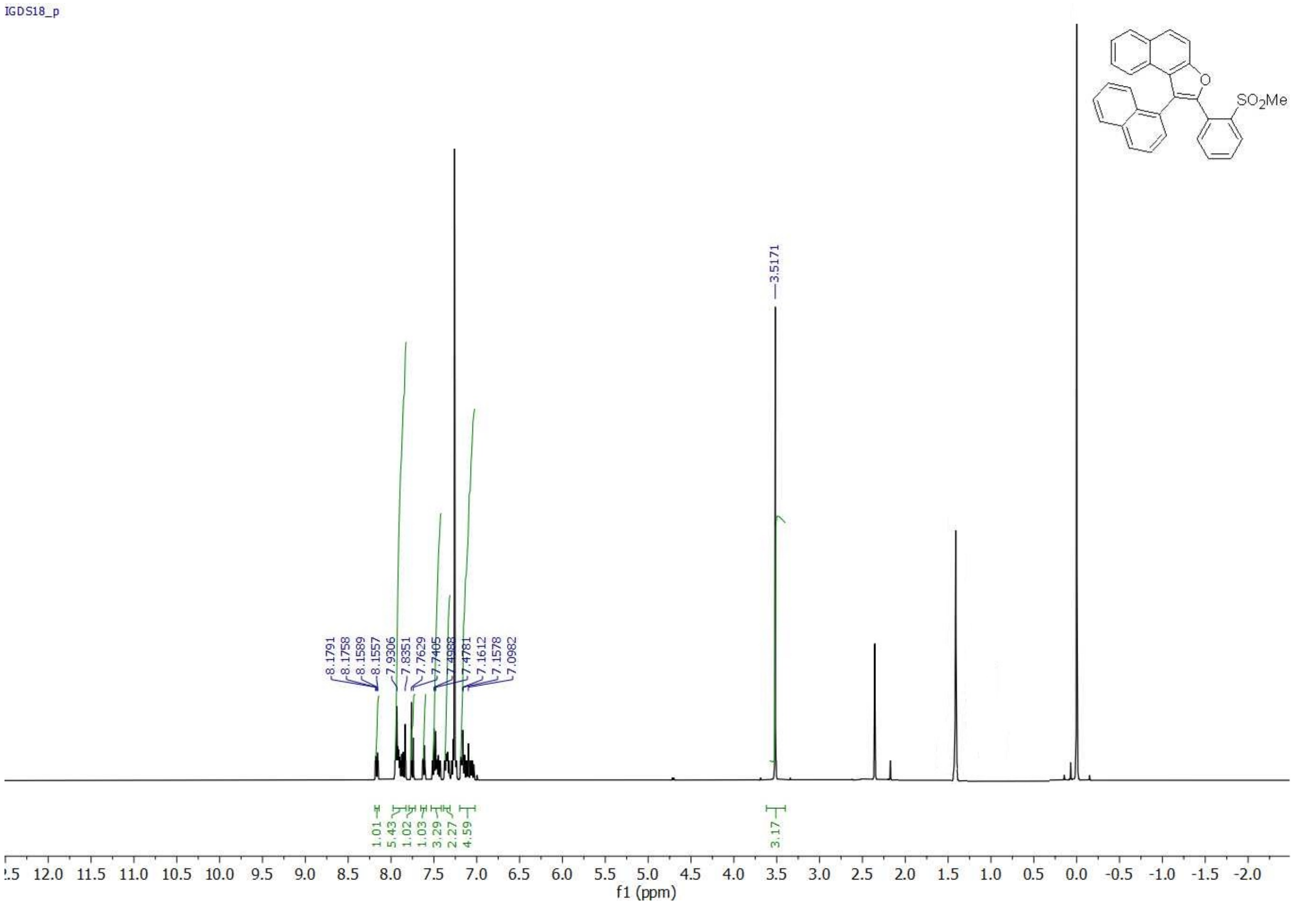

IGDS18_p
SO2Me
8.1791
8.1758
8.1589
8.1557
7.9306
7.8351
7.7629
7.7405
7.4988
7.4781
7.1612
7.1578
7.0982
3.5171
1.01
5.43
1.02
1.03
3.29
2.27
4.59
3.17
f1 (ppm)

6e

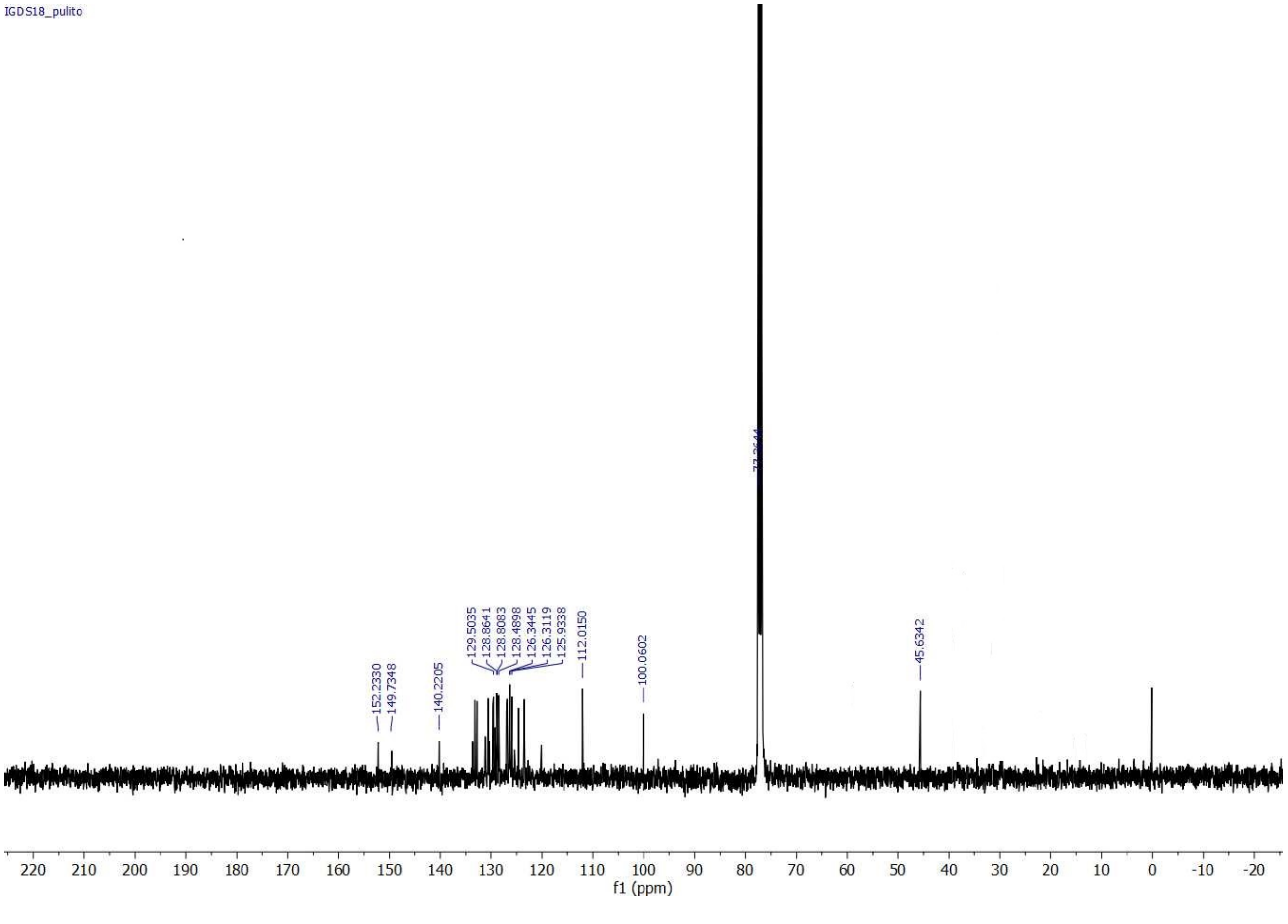

IGDS18_pulito
152.2330
149.7348
140.2205
129.5035
128.8641
128.8083
128.4898
126.3445
126.3119
125.9338
112.0150
100.0602
45.6342
f1 (ppm)

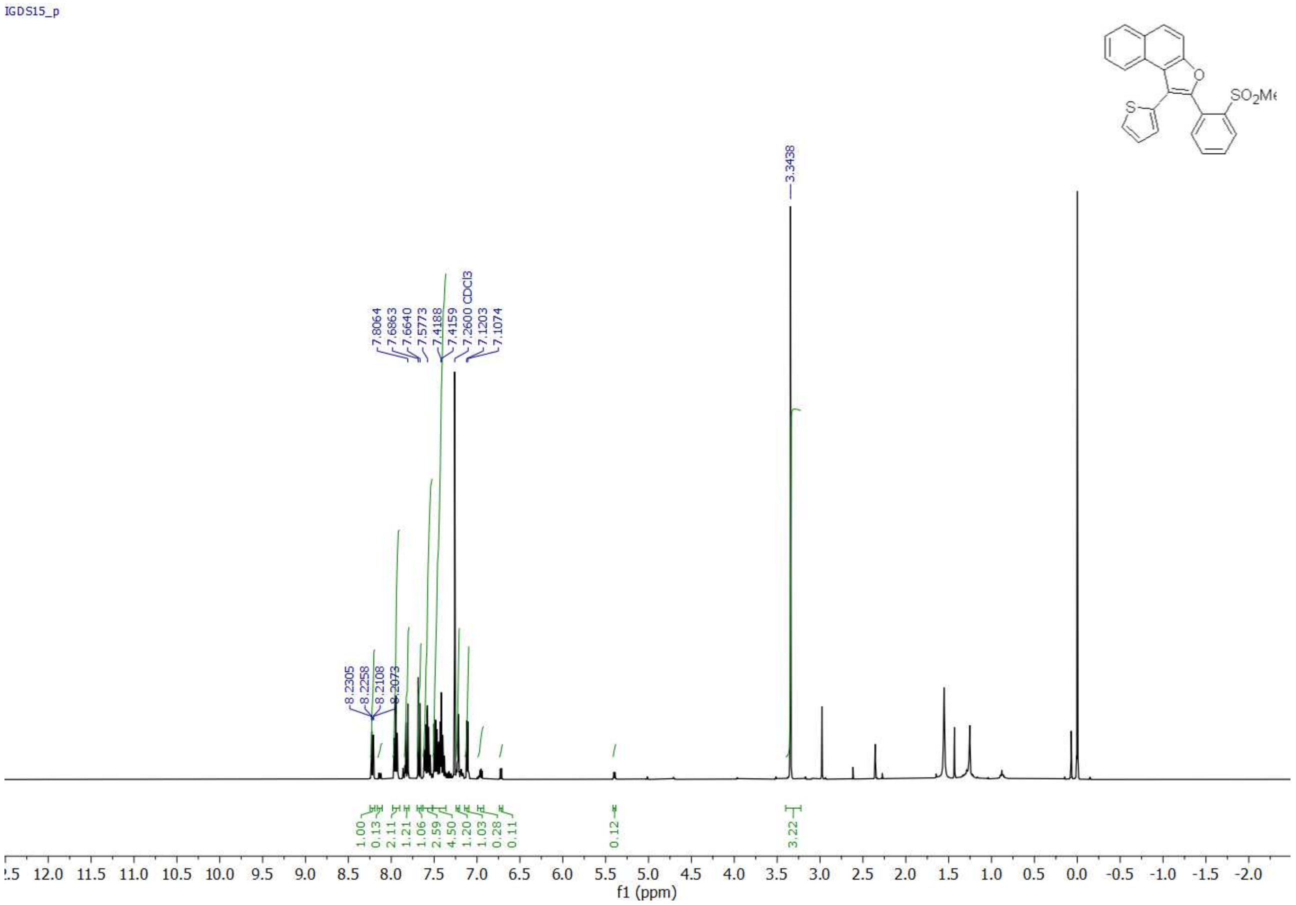
IGDS15_p
SO2Me
6f
3.3438
7.8064
7.6863
7.6640
7.5773
7.4188
7.4159
7.2600 CDCl3
7.1203
7.1074
8.2305
8.2258
8.2108
8.2073
1.00
0.13
2.11
1.21
1.06
2.59
4.50
1.20
1.03
0.28
0.11
0.12
3.22
f1 (ppm)

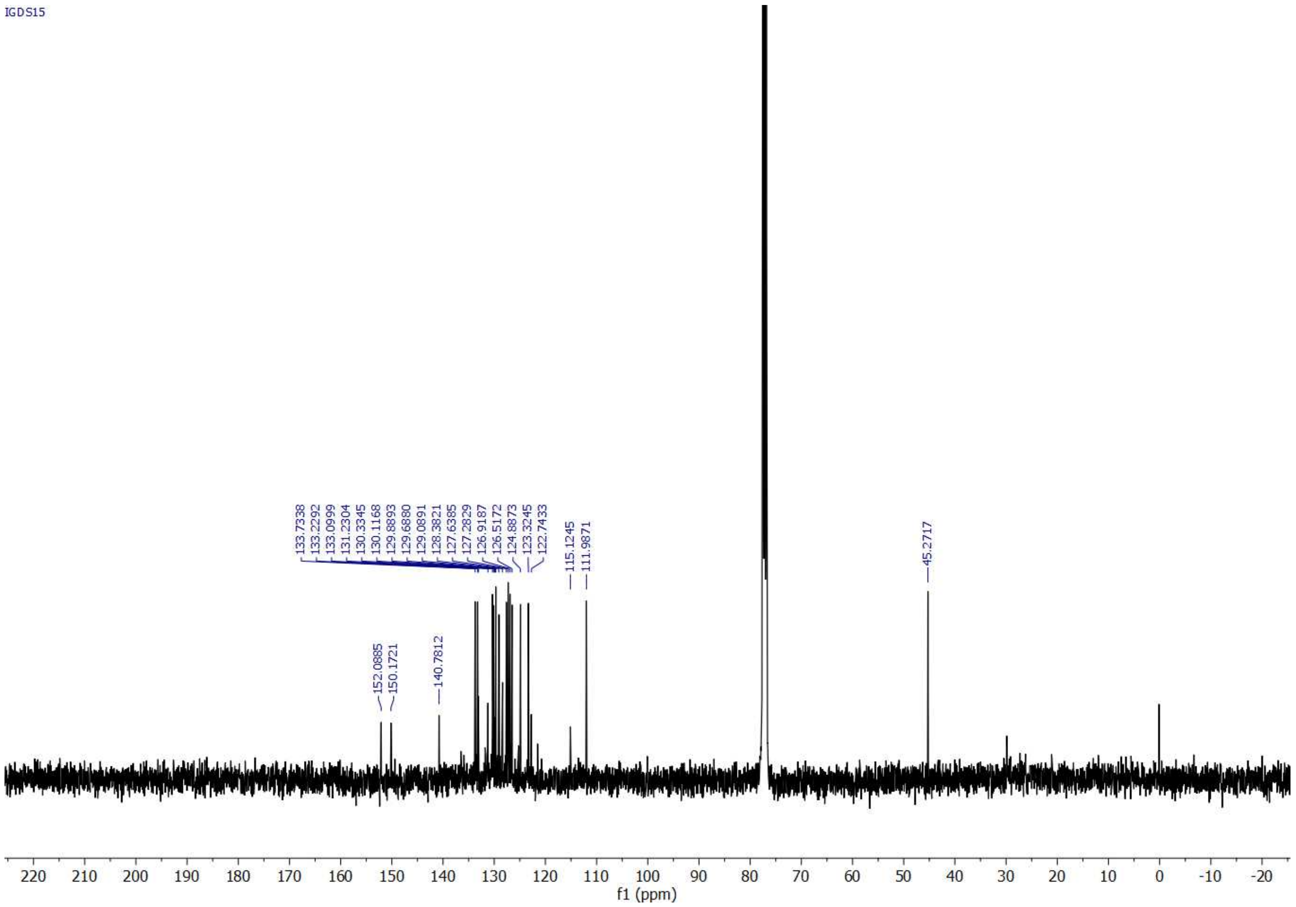
IGDS15
133.7338
133.2292
133.0999
131.2304
130.3345
130.1168
129.8893
129.6880
129.0891
128.3821
127.6385
127.2829
126.9187
126.5172
124.8873
123.3245
122.7433
115.1245
111.9871
45.2717
152.0885
150.1721
140.7812
f1 (ppm)

VBNZ70-72_subs_aromatizz
single_pulse

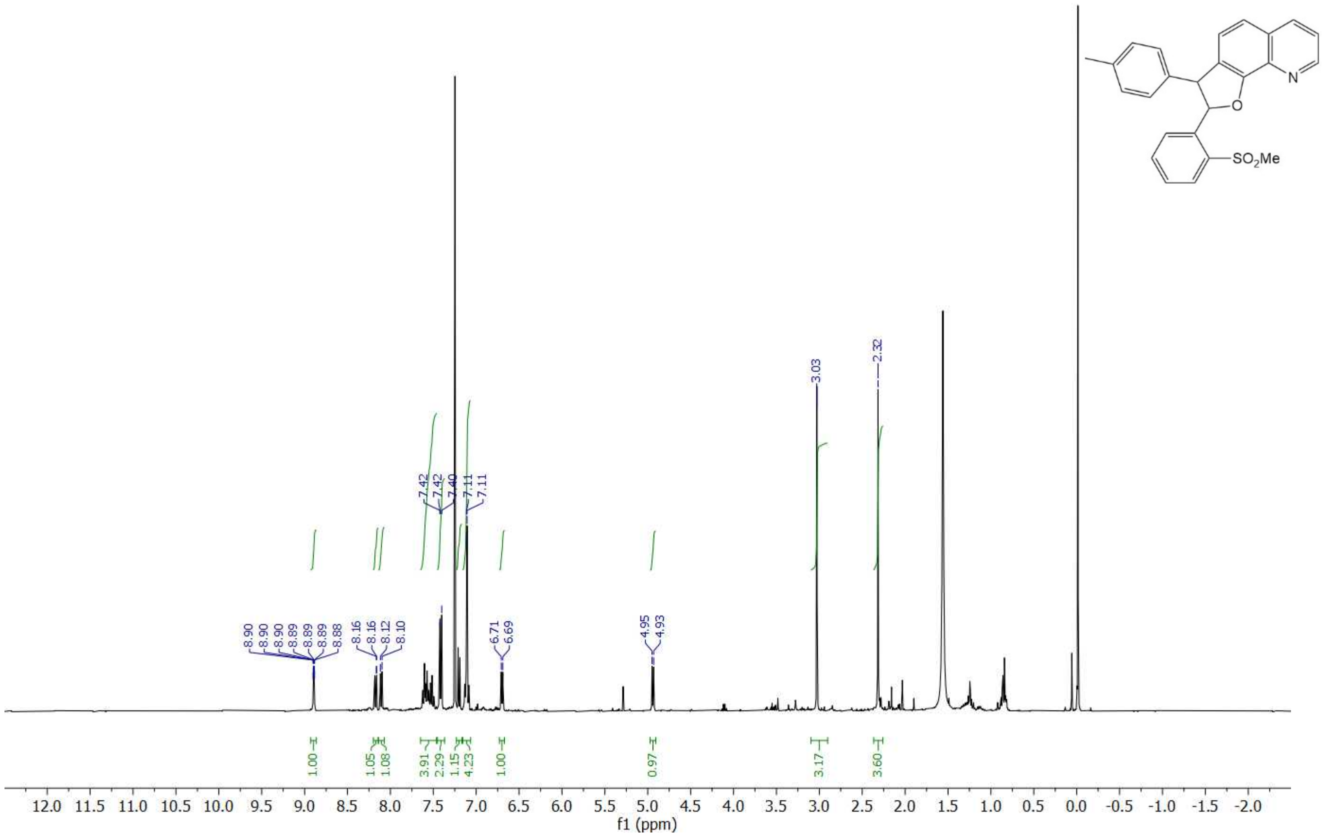

7a

VBNZ70-72_subs_aromatizz
single pulse decoupled gated NOE

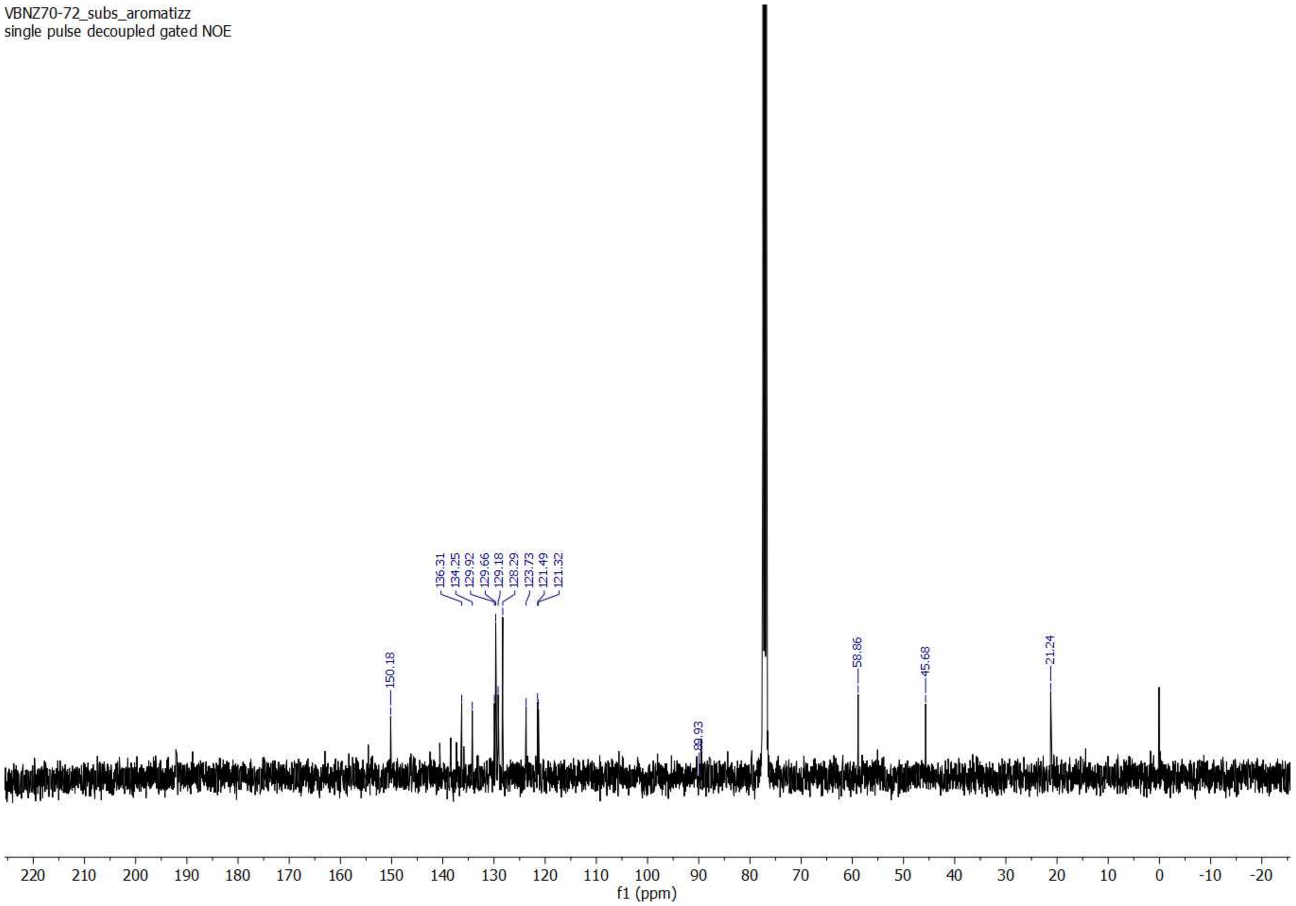

VIBNZ2_grezzo
single_pulse

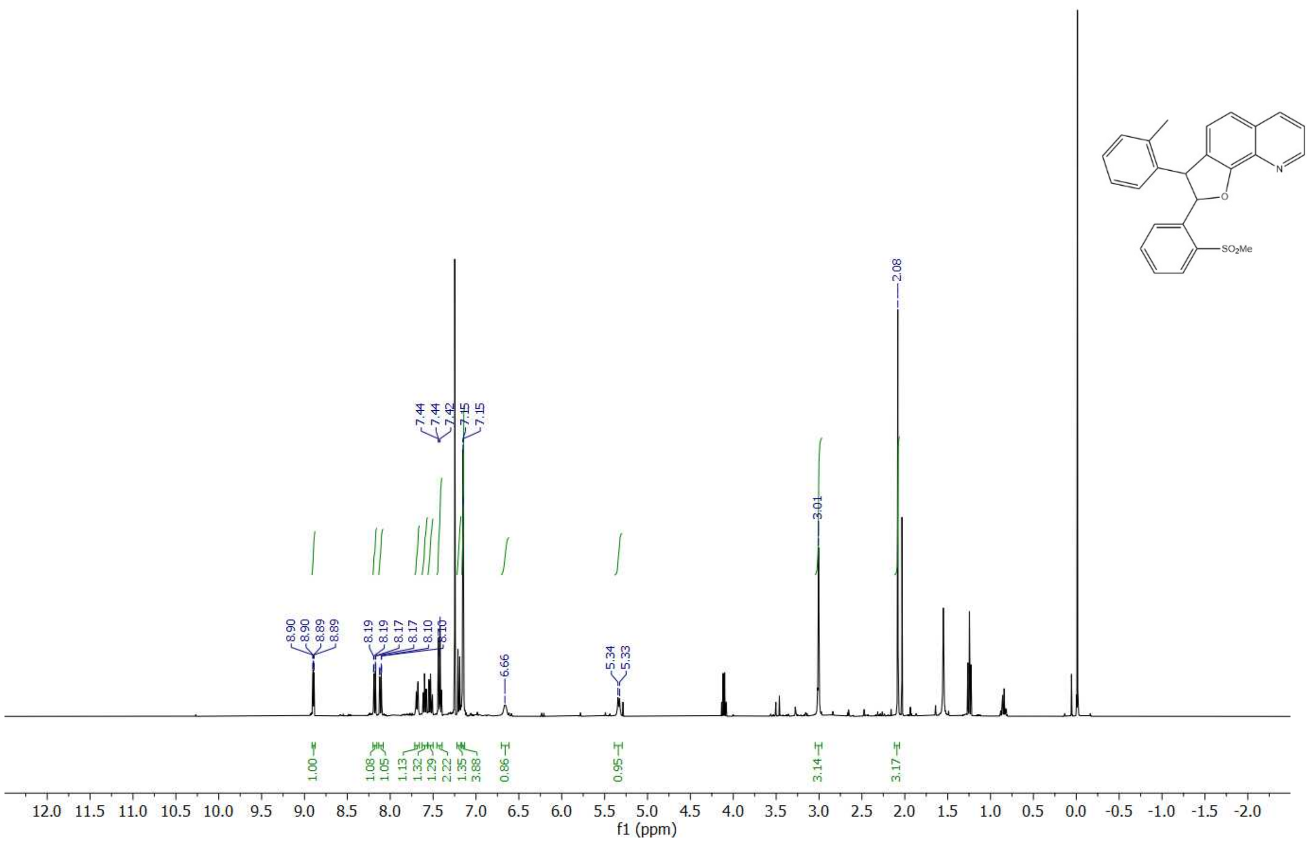

7b

DNF_o-Tol_HQ_ripEP-CM
single pulse decoupled gated NOE

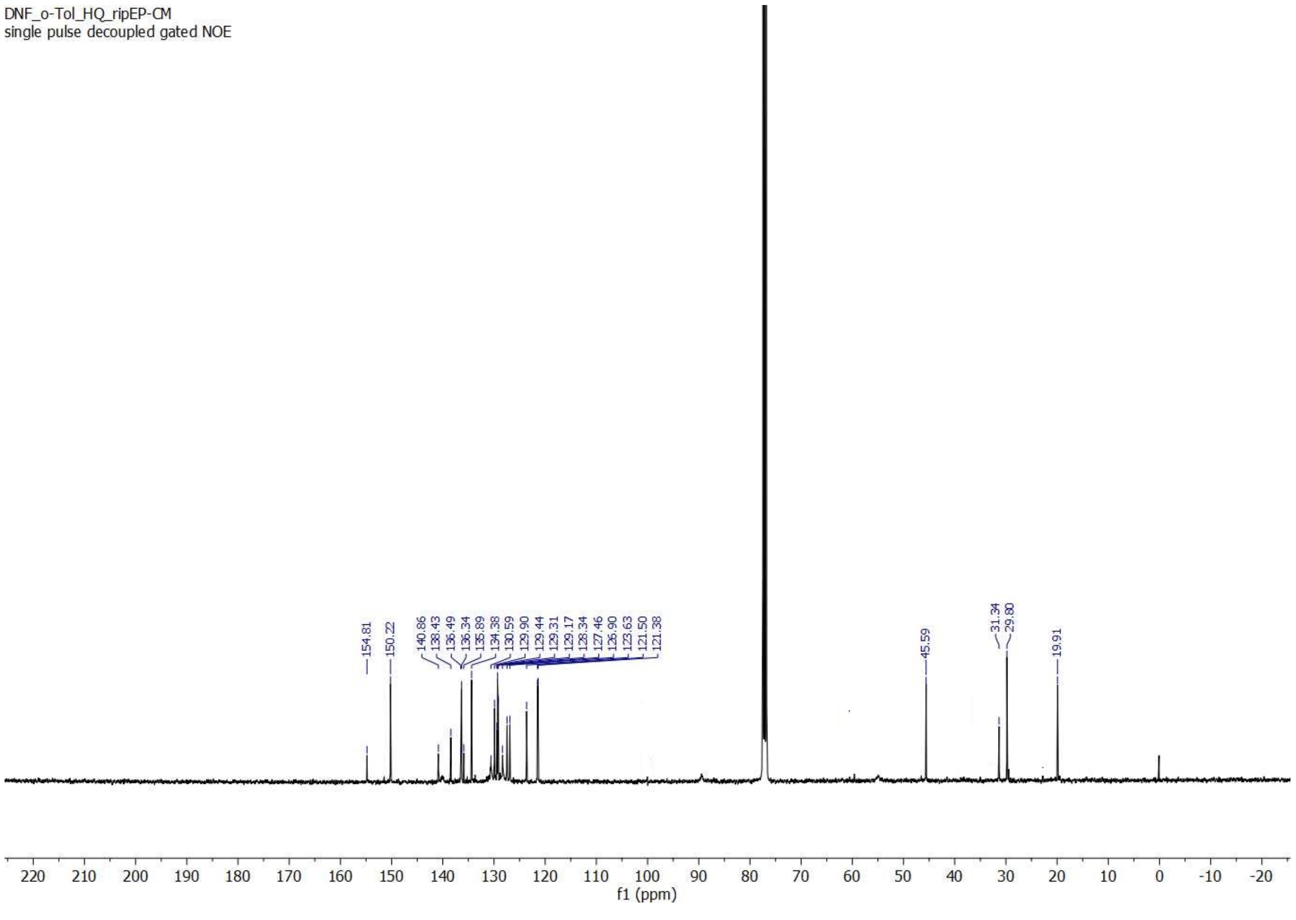

DNF_p-OMe_naftolo_VIBNZ29

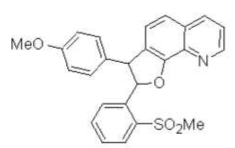

7c

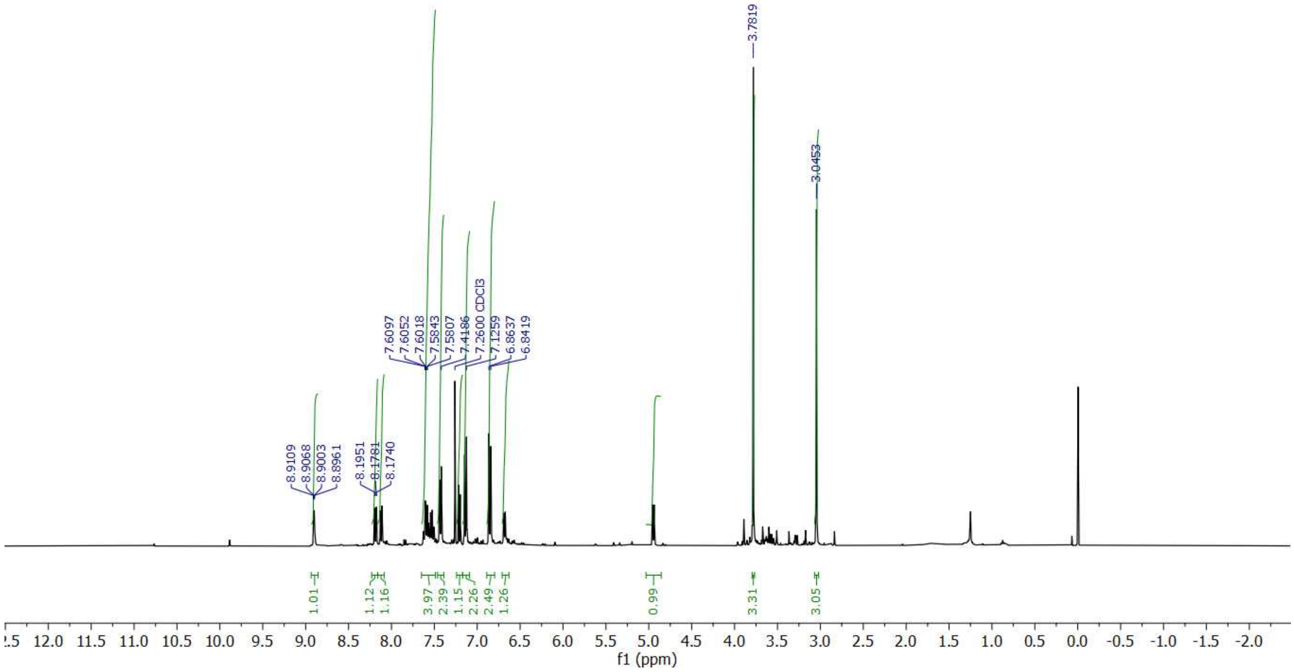

DNF_p-OMe_naftolo_VIBNZ29

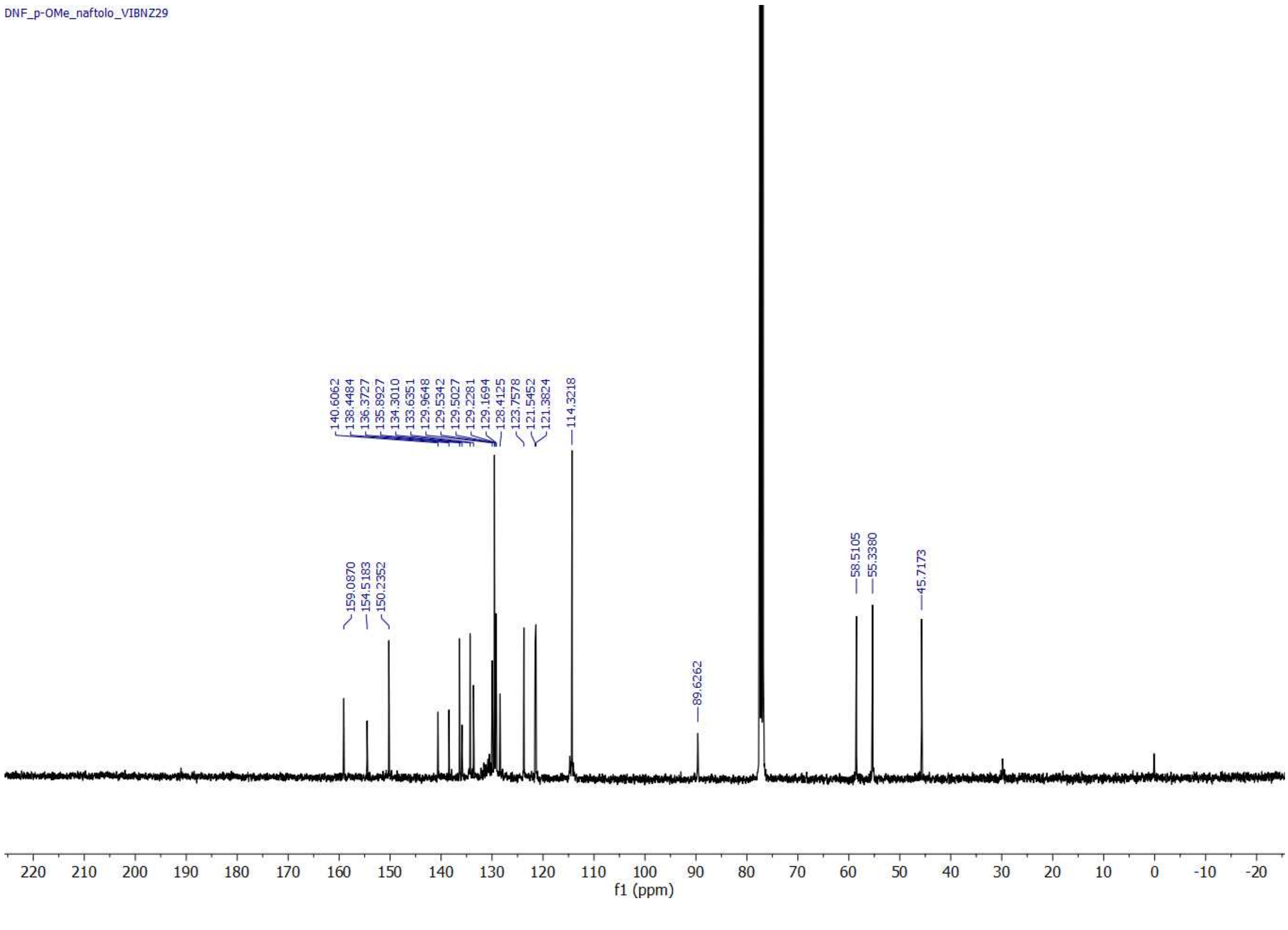

DFQ_HQ-pCl_xxEtOH
single_pulse

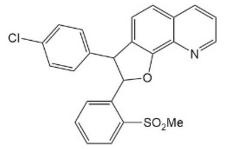

7d

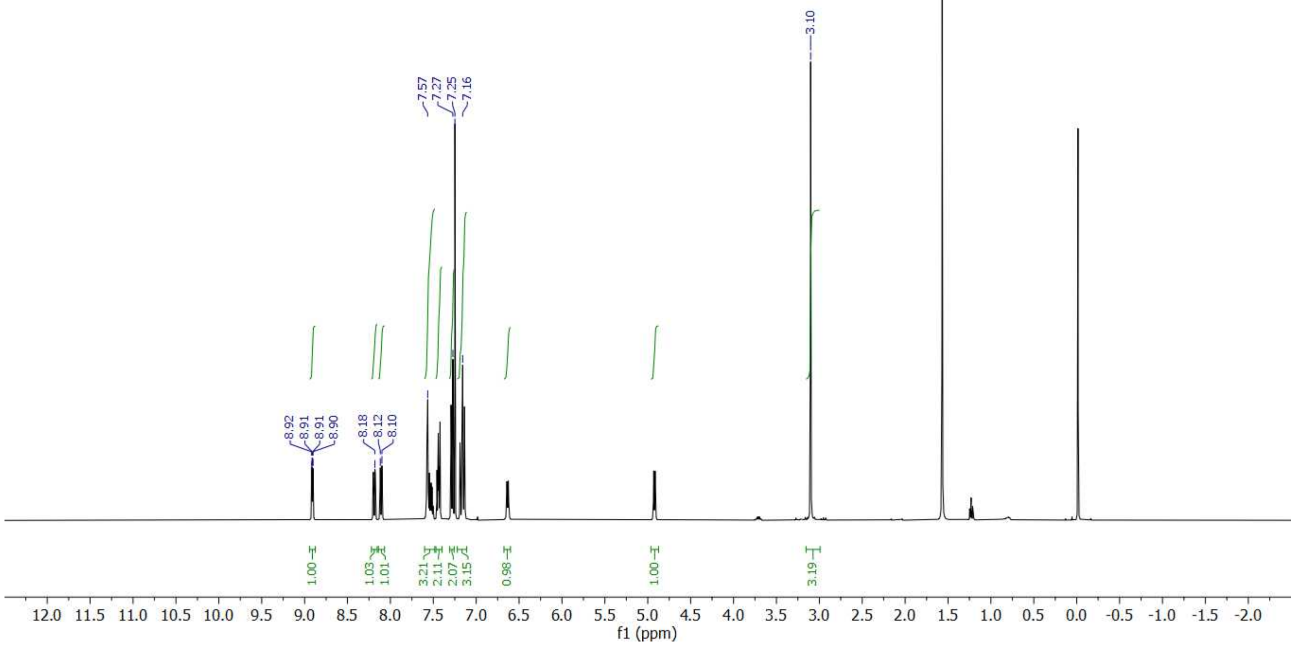

DFQ_pCl_HQ
single pulse decoupled gated NOE

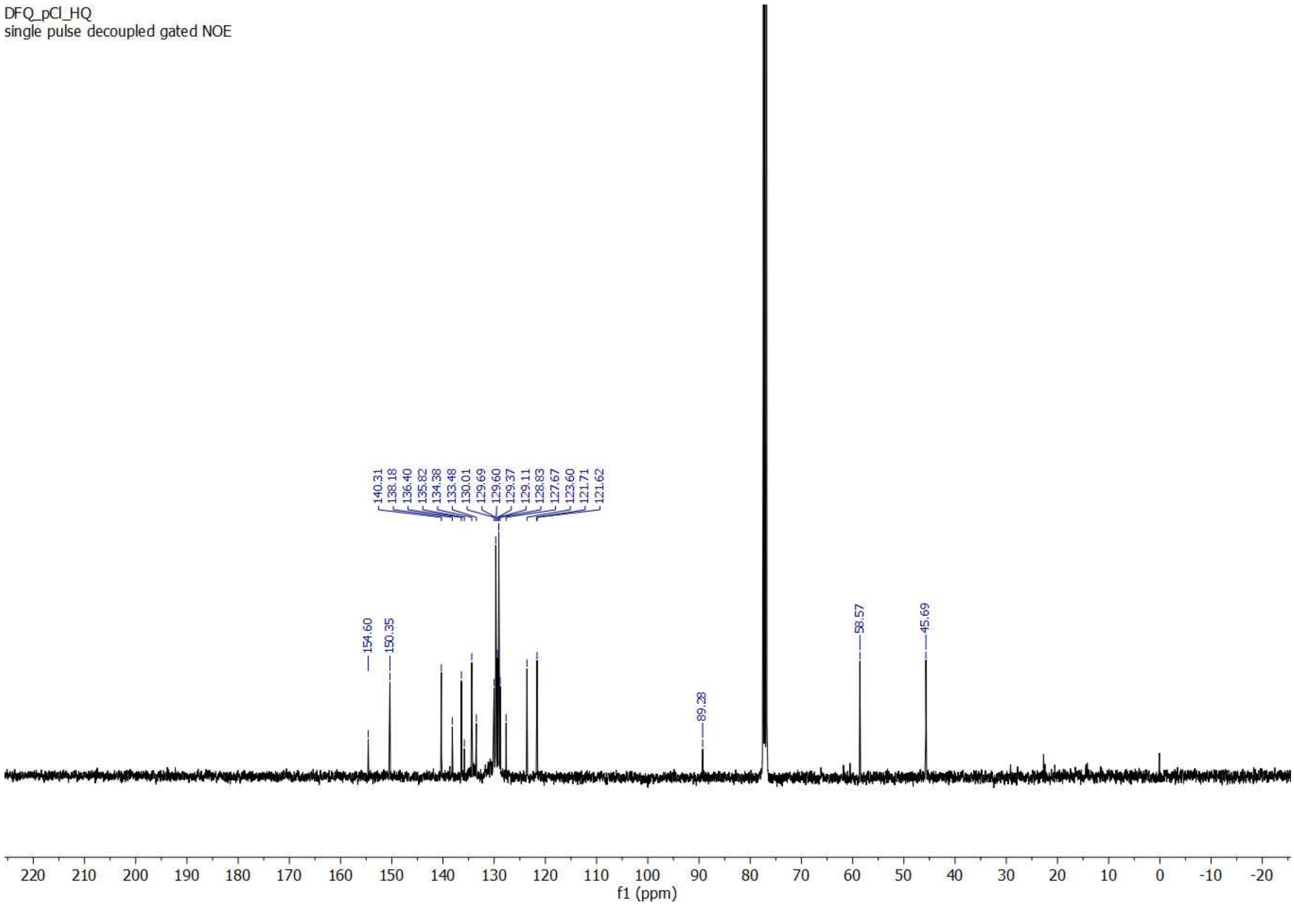

NF_ptol_HQ_VIBNZ27

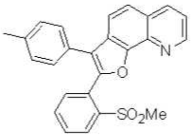

8a

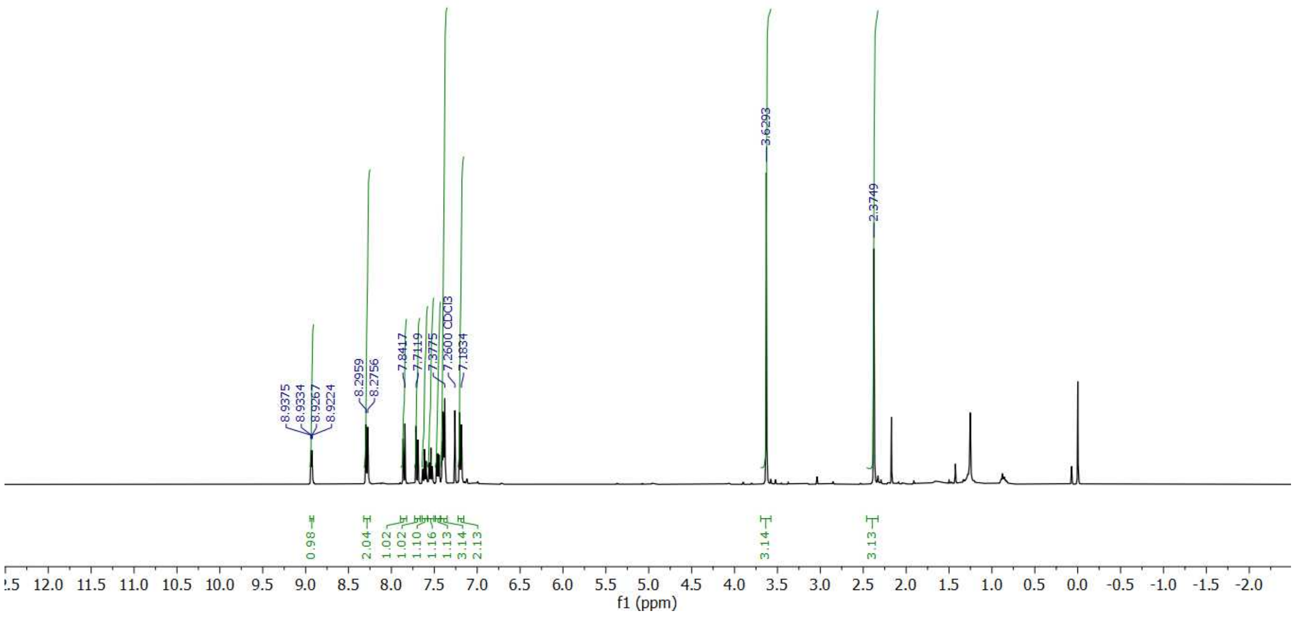

NF_ptol_HQ_VIBNZ27

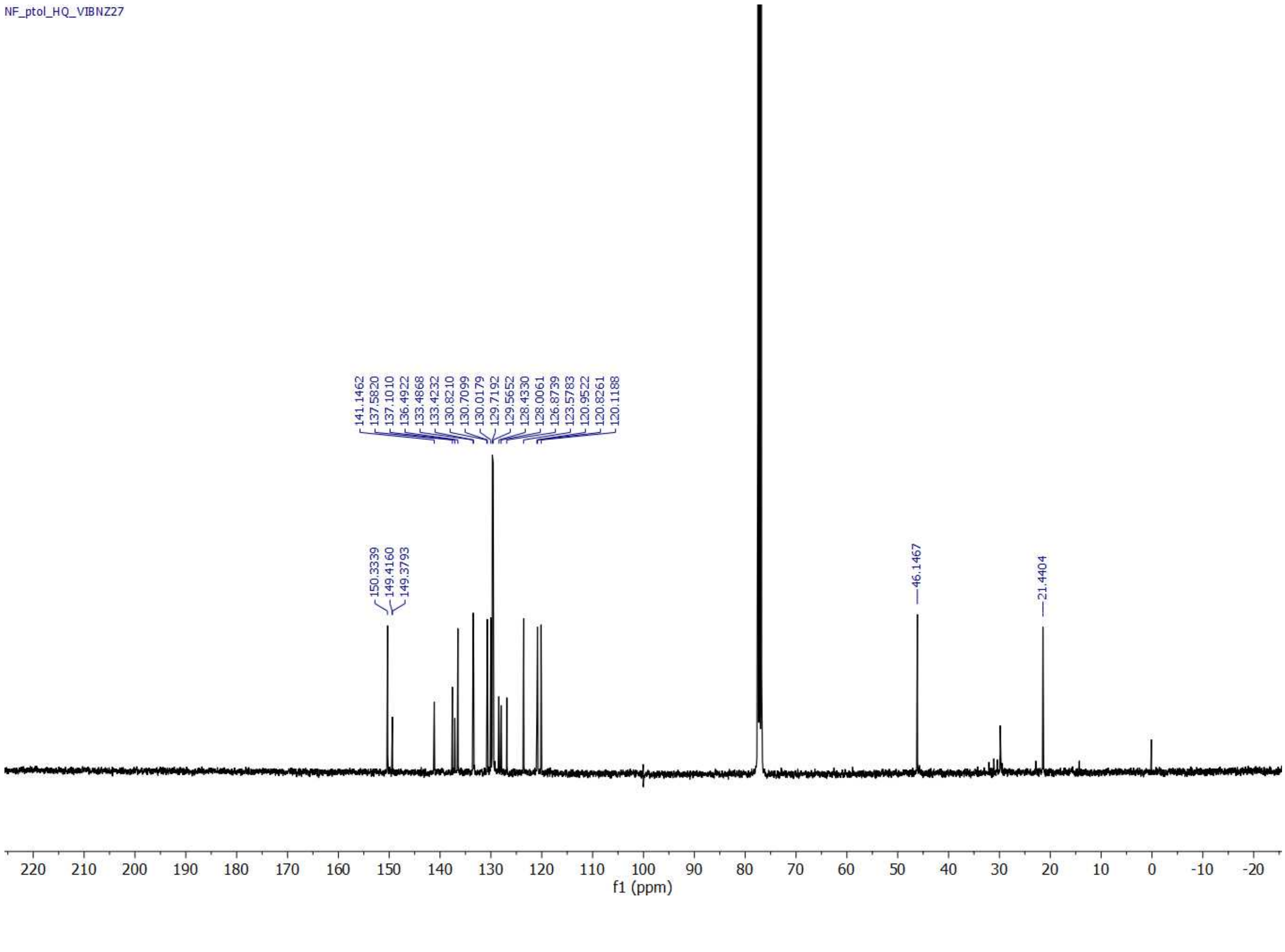

VIBNZ33

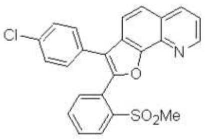

8d

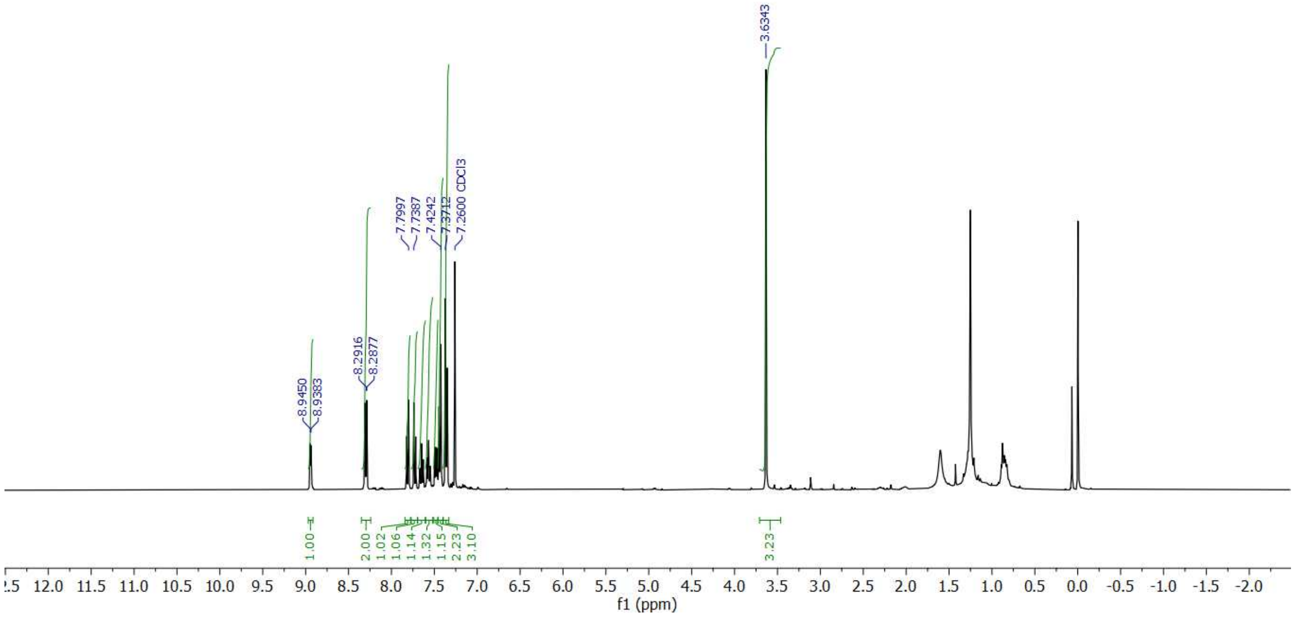

VIBNZ33

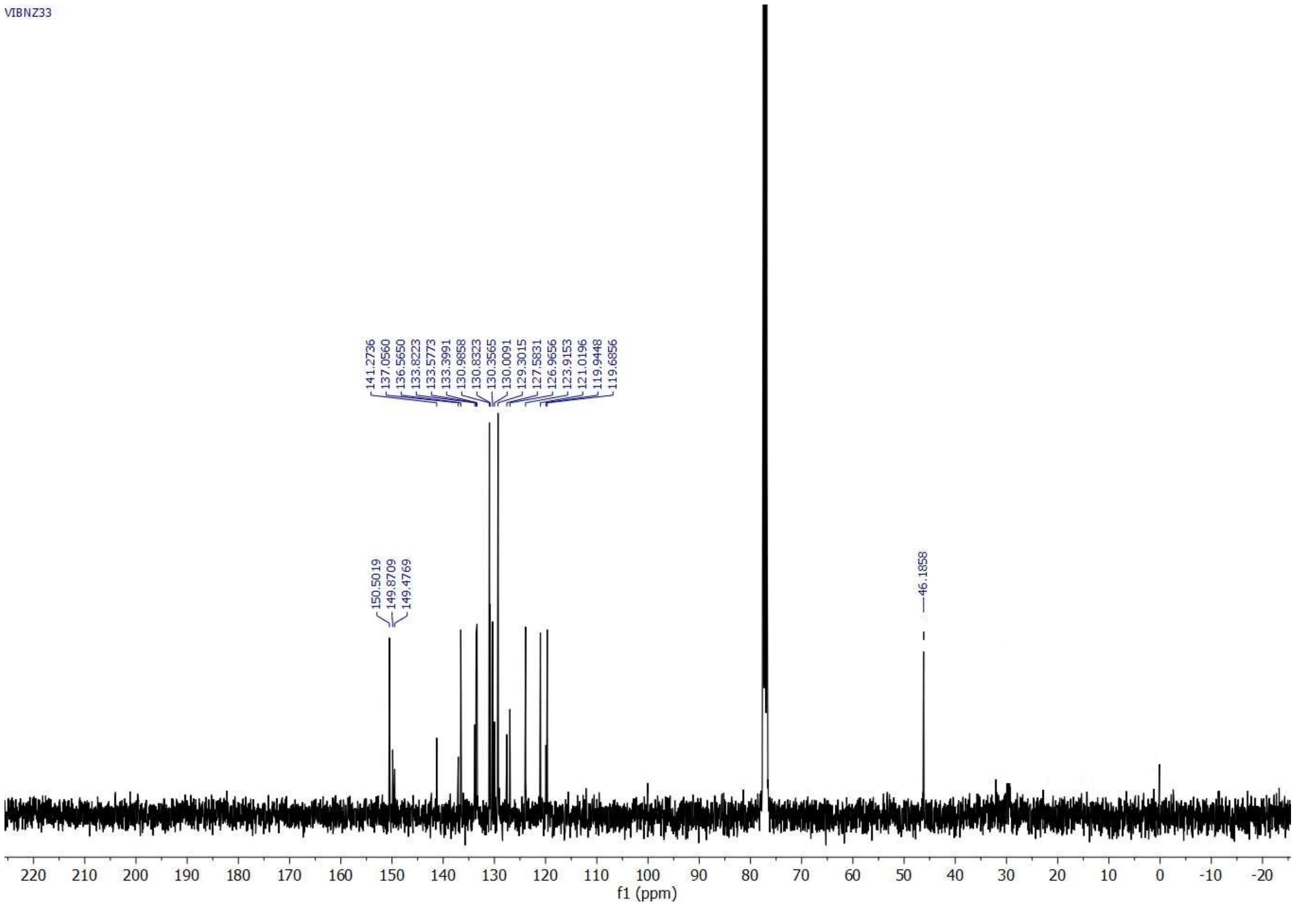

VBNZ80_hydroxycoumarin
single_pulse

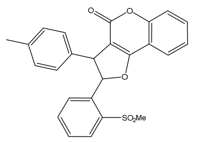

9a

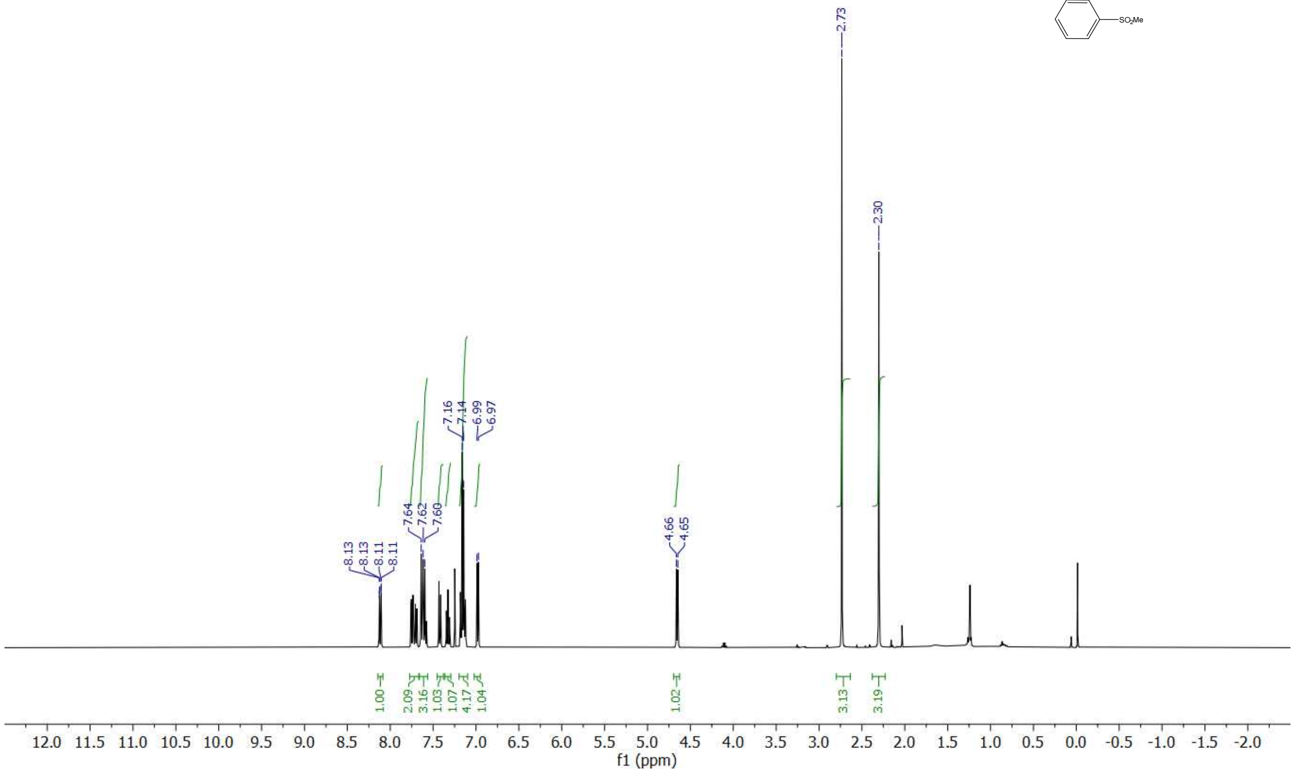

VBNZ80_hydroxycoumarin
single pulse decoupled gated NOE

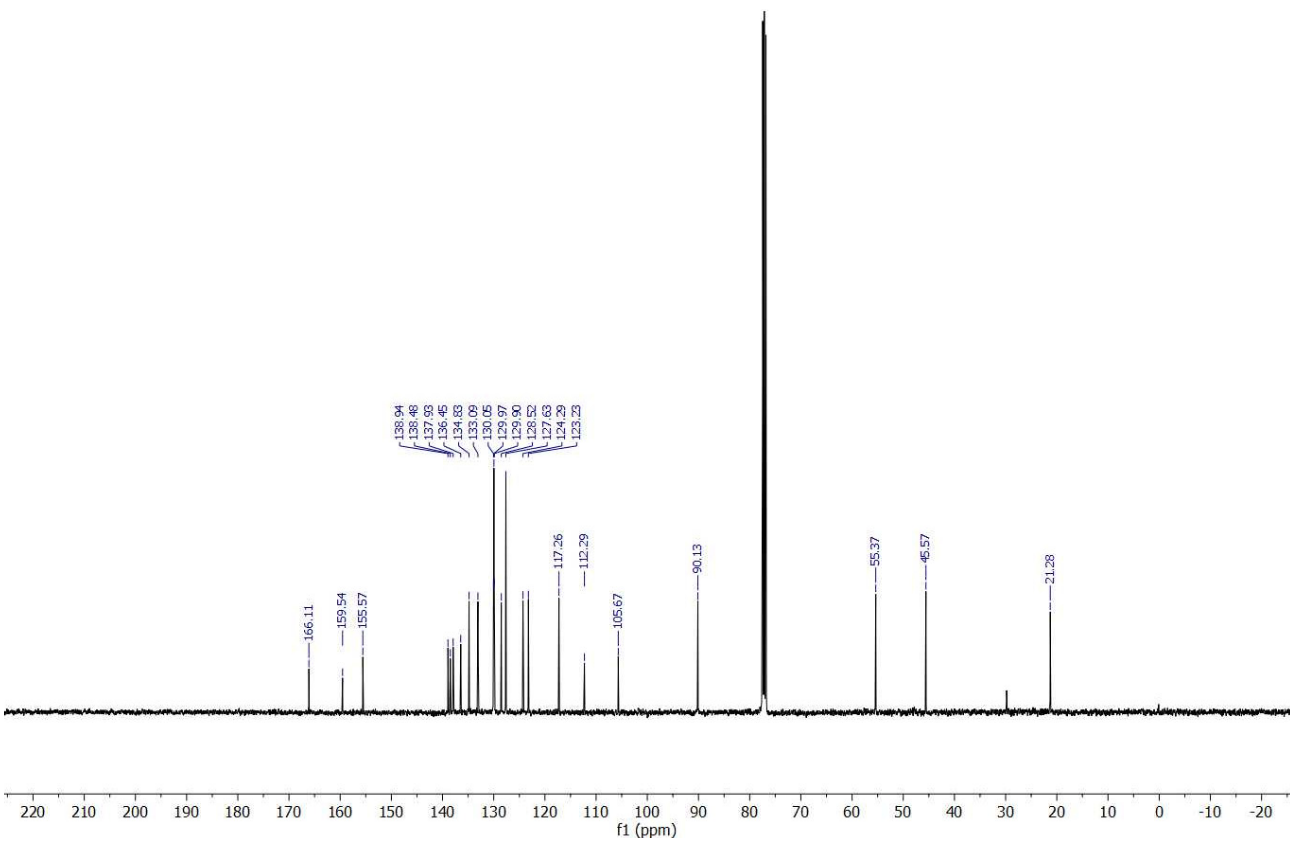

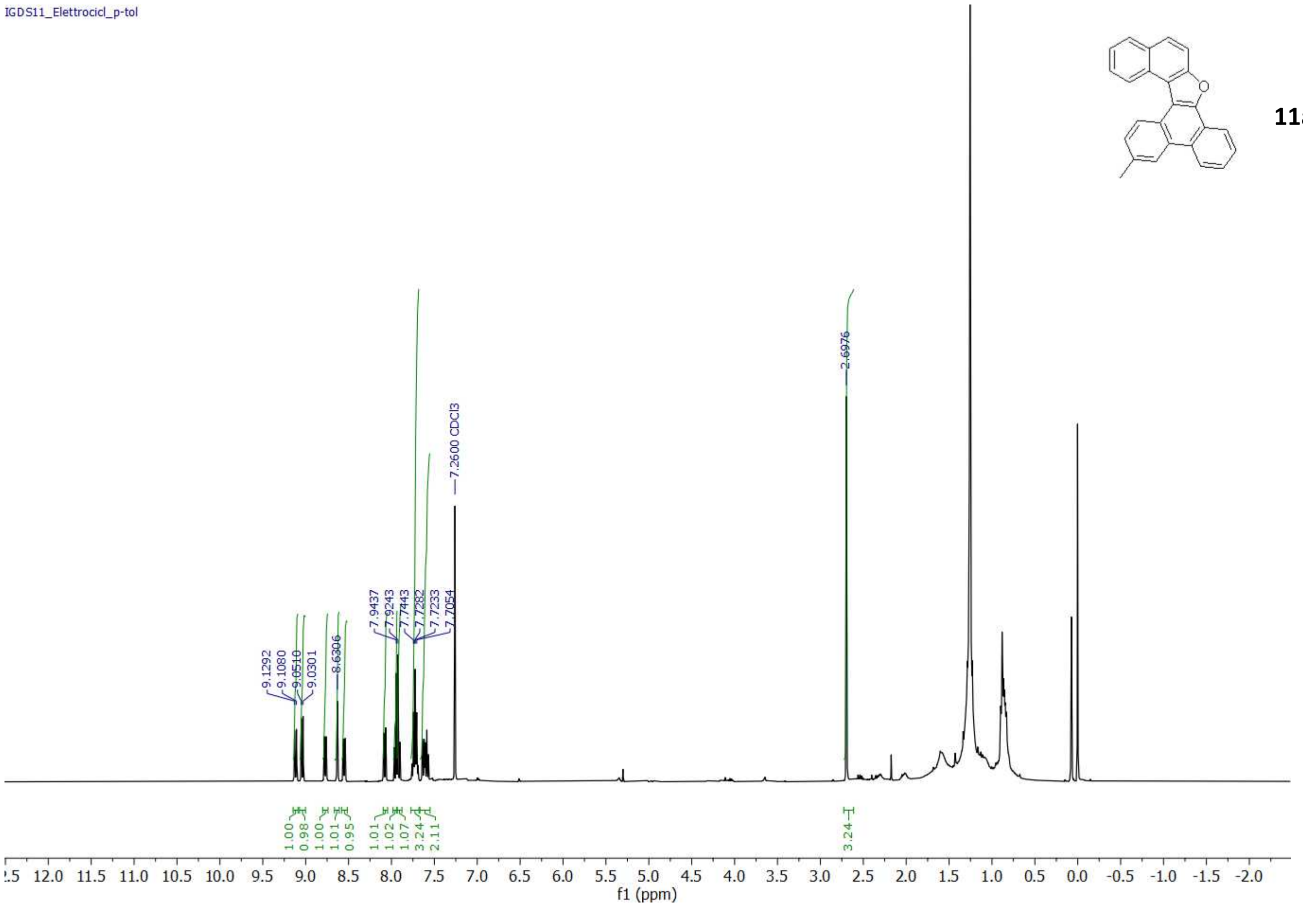
IGDS11_Elettrocicl_p-tol
9.1292
9.1080
9.0510
9.0301
8.6306
7.9437
7.9243
7.7443
7.7282
7.7233
7.7054
7.2600 CDCl3
2.6976
1.00
0.98
1.00
1.01
0.95
1.01
1.02
1.07
3.24
2.11
3.24
f1 (ppm)

11a

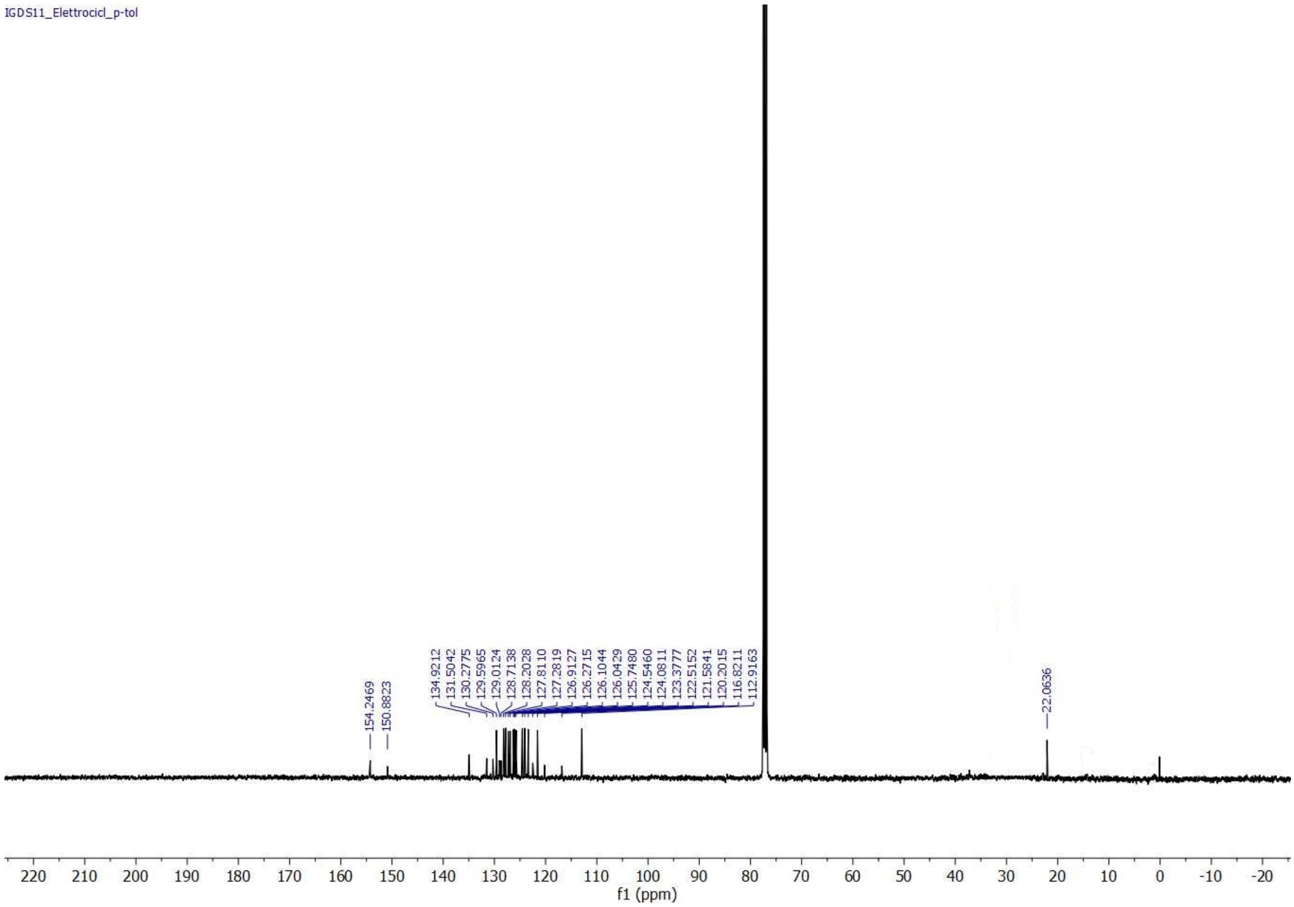
IGDS11_Elettrocicl_p-tol
154.2469
150.8823
134.9212
131.5042
130.2775
129.5965
129.0124
128.7138
128.2028
127.8110
127.2819
126.9127
126.2715
126.1044
126.0429
125.7480
124.5460
124.0811
123.3777
122.5152
121.5841
120.2015
116.8211
112.9163
22.0636
f1 (ppm)

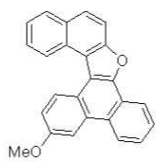
MeO
O

11c

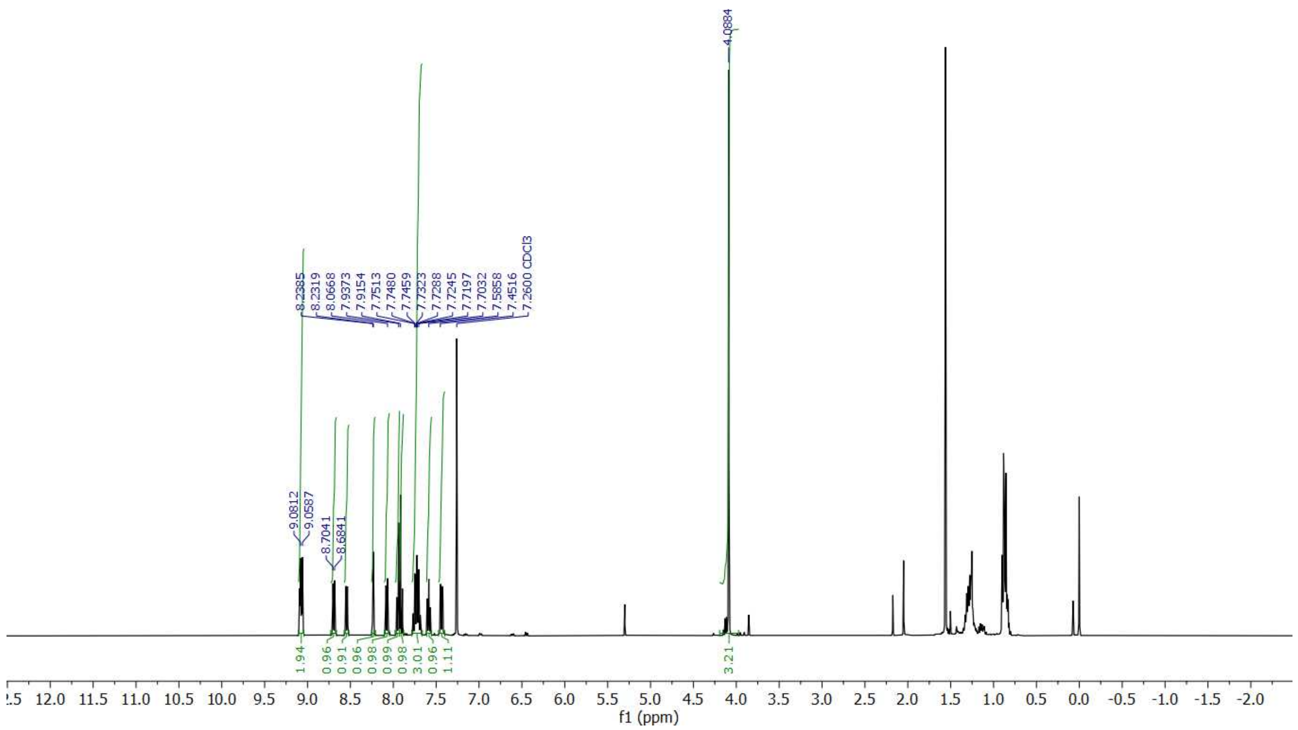
IGDS20_fr3,4-5
9.0812
9.0587
8.7041
8.6841
8.2385
8.2319
8.0668
7.9373
7.9154
7.7513
7.7480
7.7459
7.7323
7.7288
7.7245
7.7197
7.7032
7.5858
7.4516
7.2600 CDCl3
4.0884
1.94
0.96
0.91
0.96
0.98
0.99
0.98
3.01
0.96
1.11
3.21
f1 (ppm)

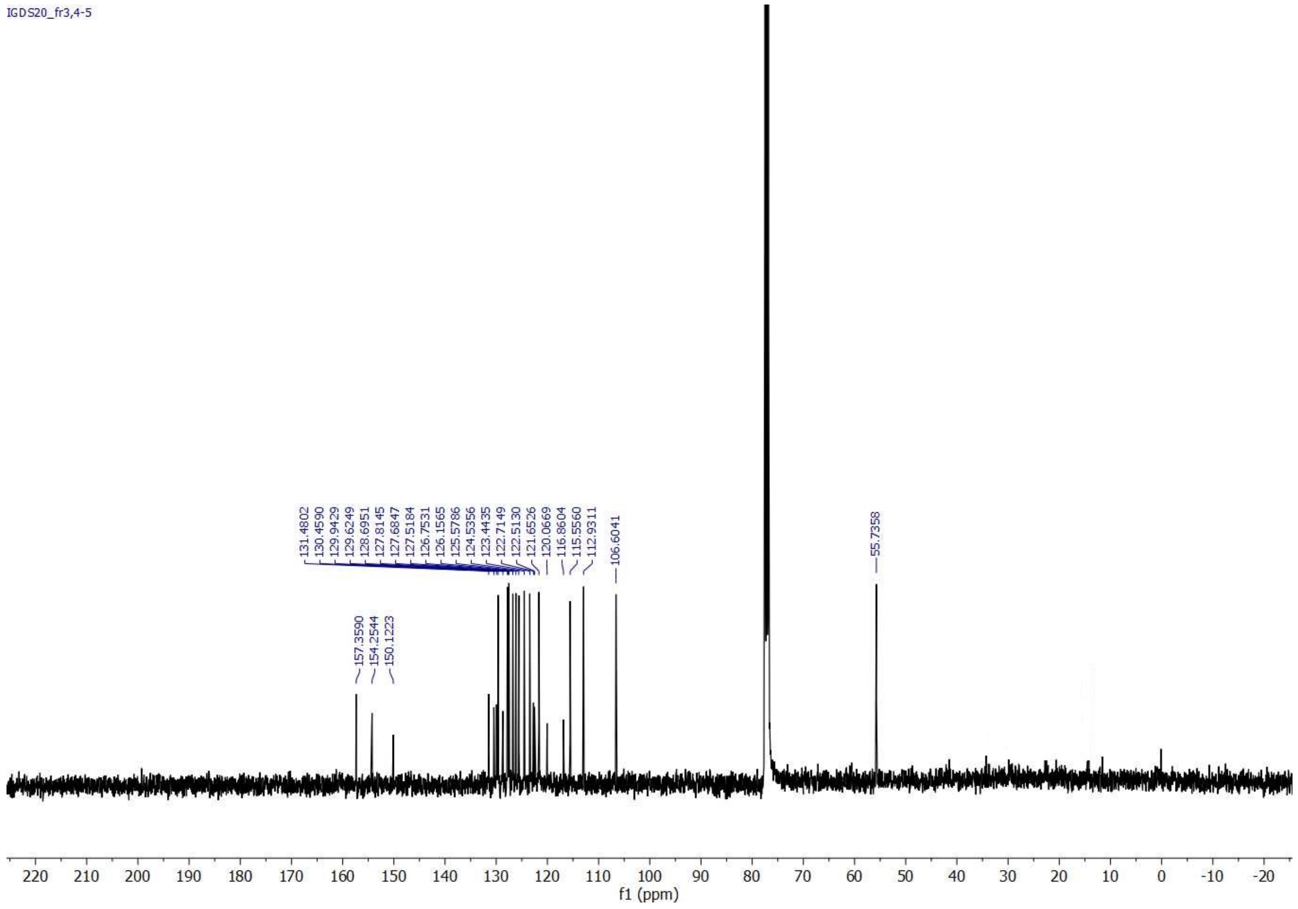
IGDS20_fr3,4-5
157.3590
154.2544
150.1223
131.4802
130.4590
129.9429
129.6249
128.6951
127.8145
127.6847
127.5184
126.7531
126.1565
125.5786
124.5356
123.4435
122.7149
122.5130
121.6526
120.0669
116.8604
115.5560
112.9311
106.6041
55.7358
f1 (ppm)

IGDS_pCl_pulito
single_pulse

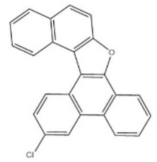

11d

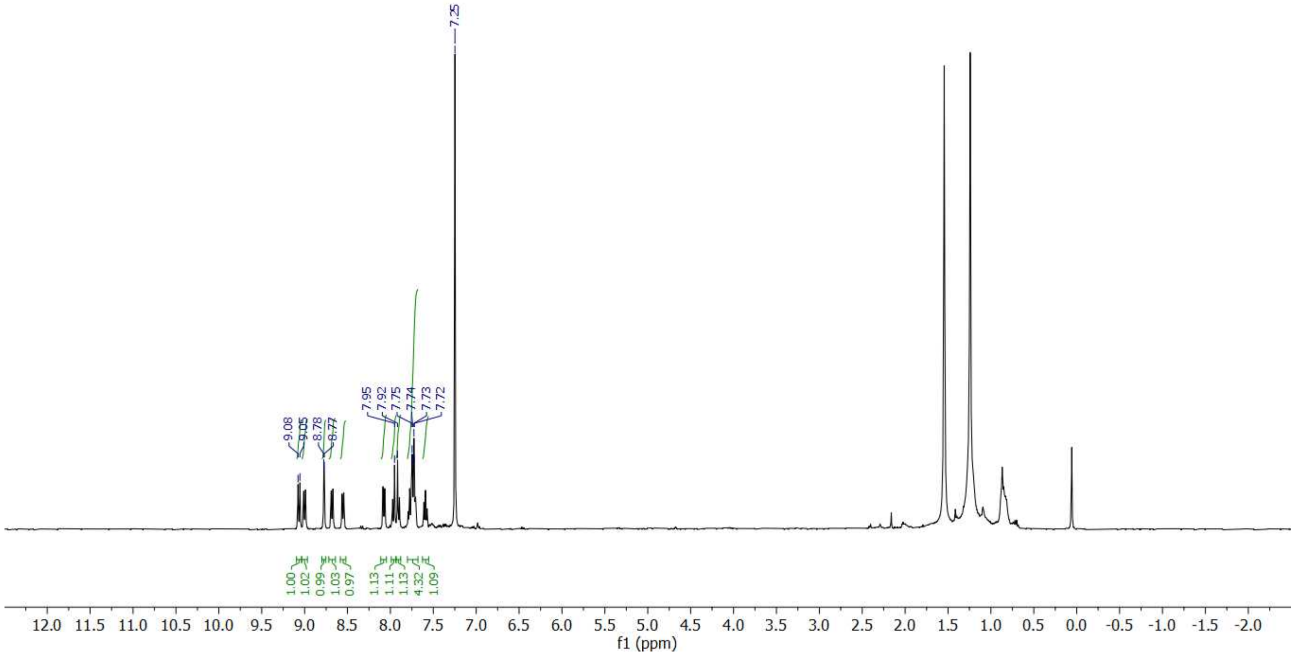

IGDS_p-Cl_elettrociclizzato_concentrato
single pulse decoupled gated NOE

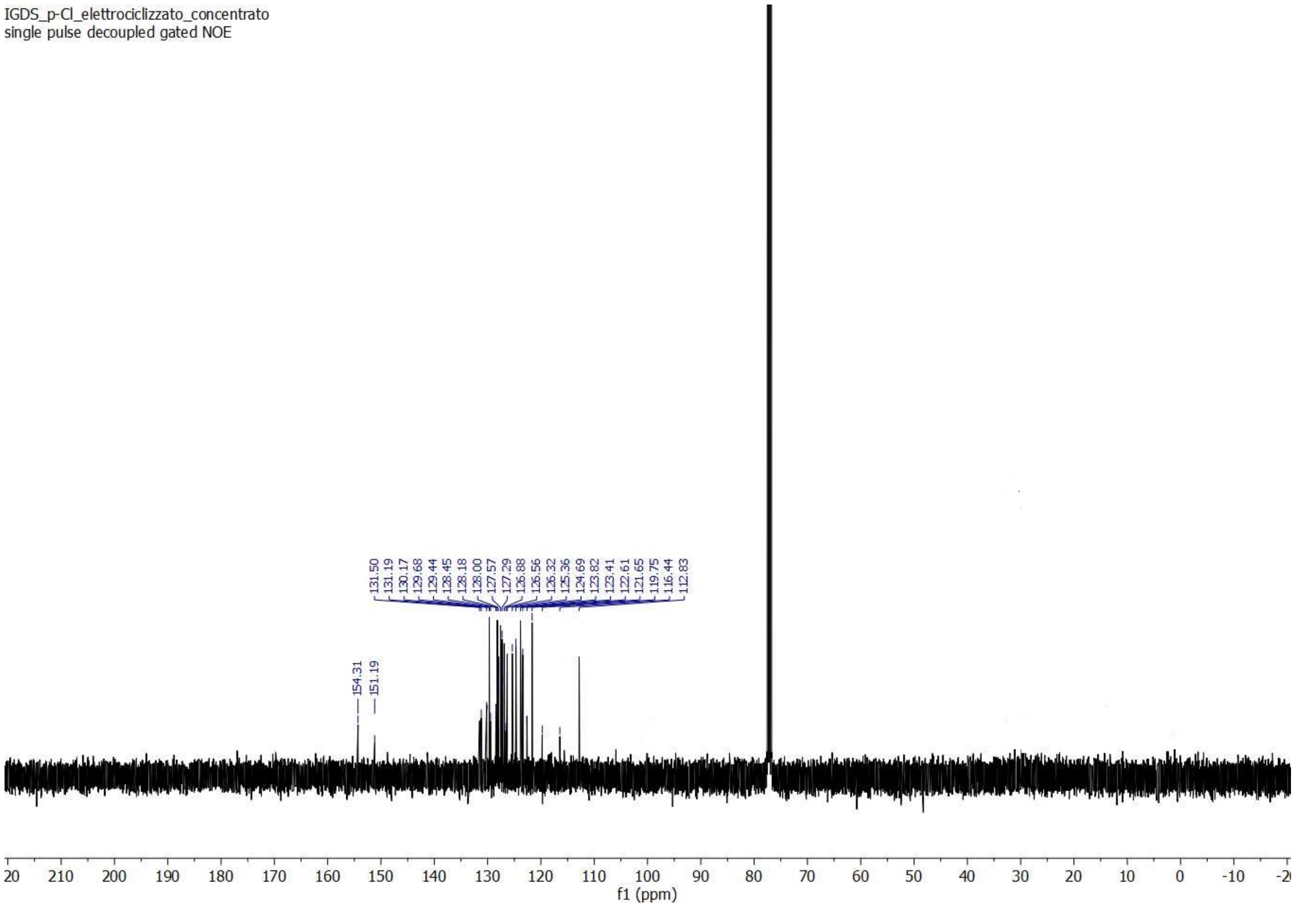

IGDS19_Elettrocicl_1-naftile

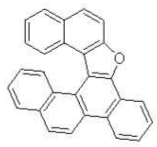

11e

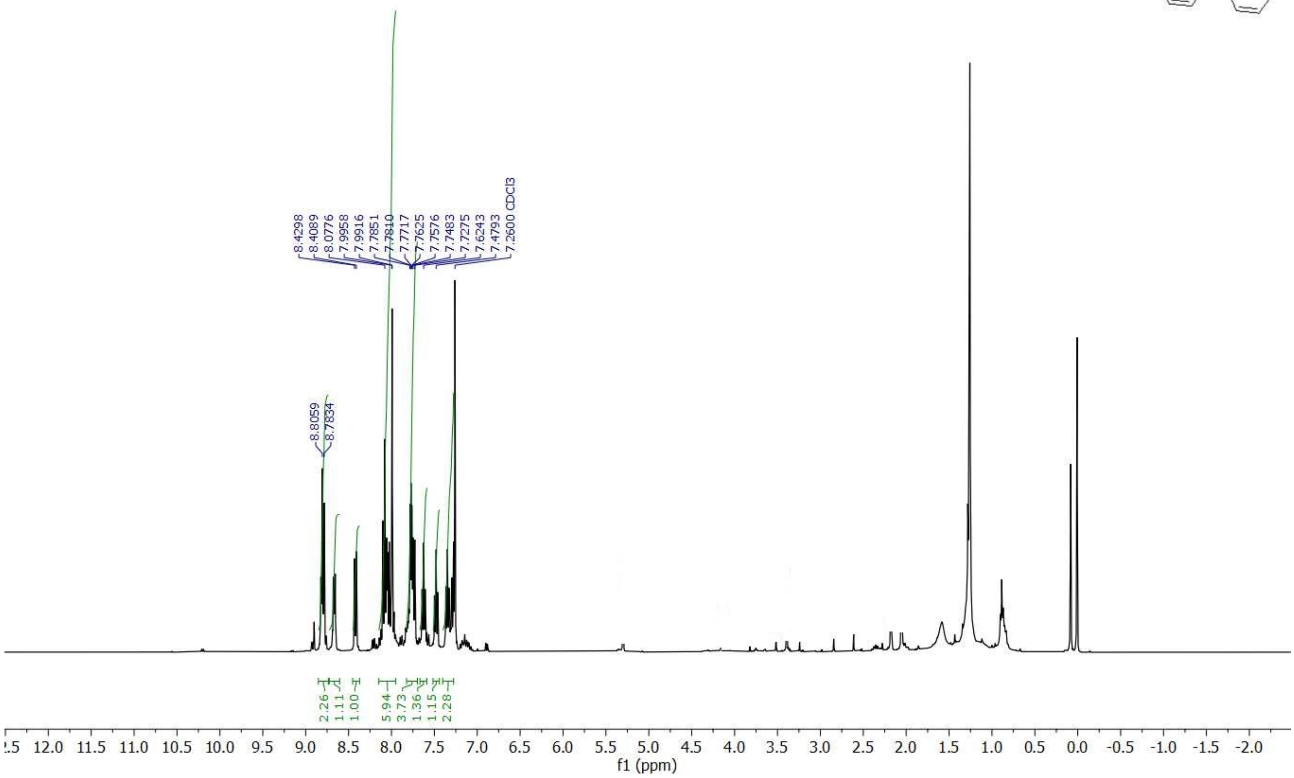

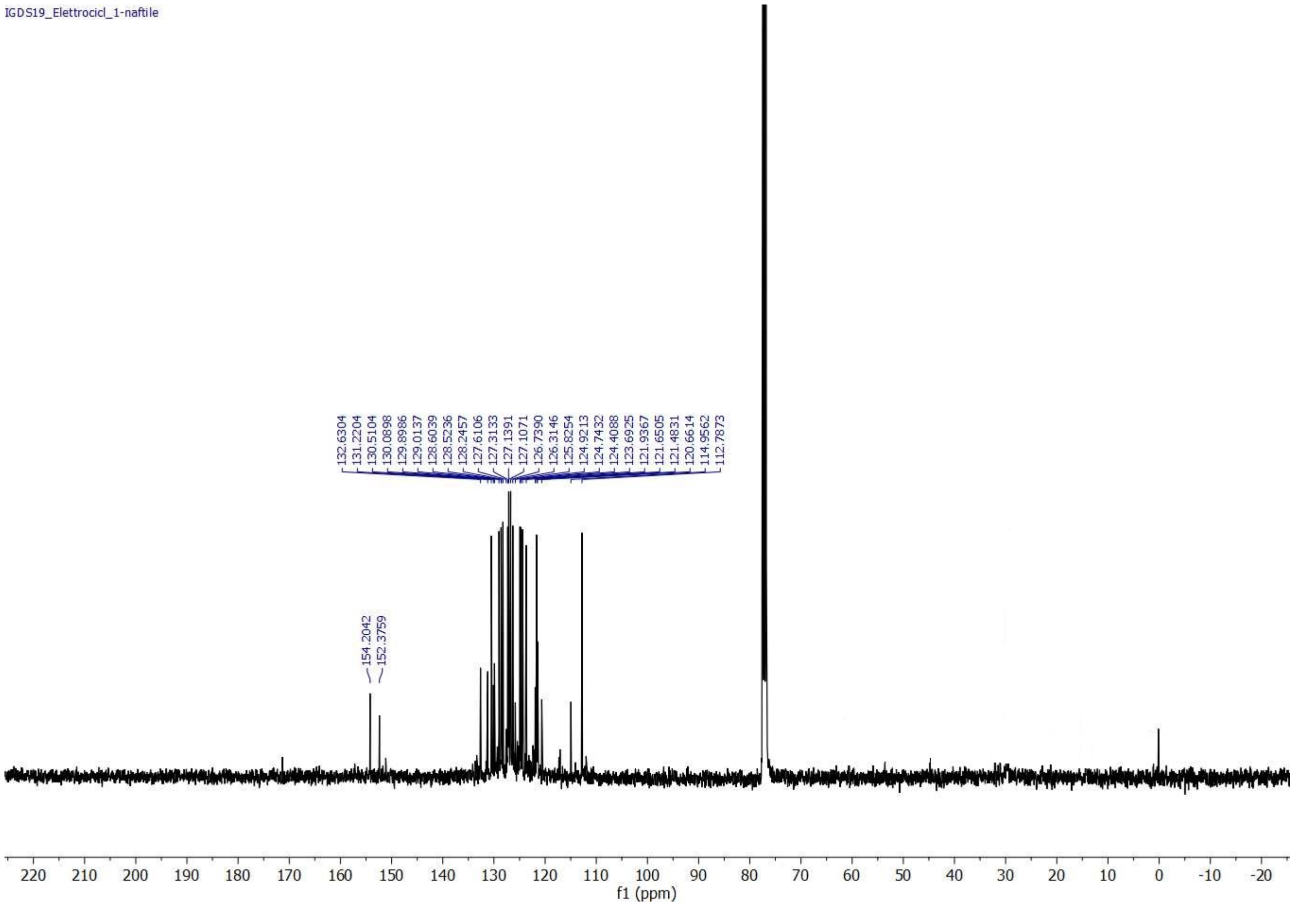

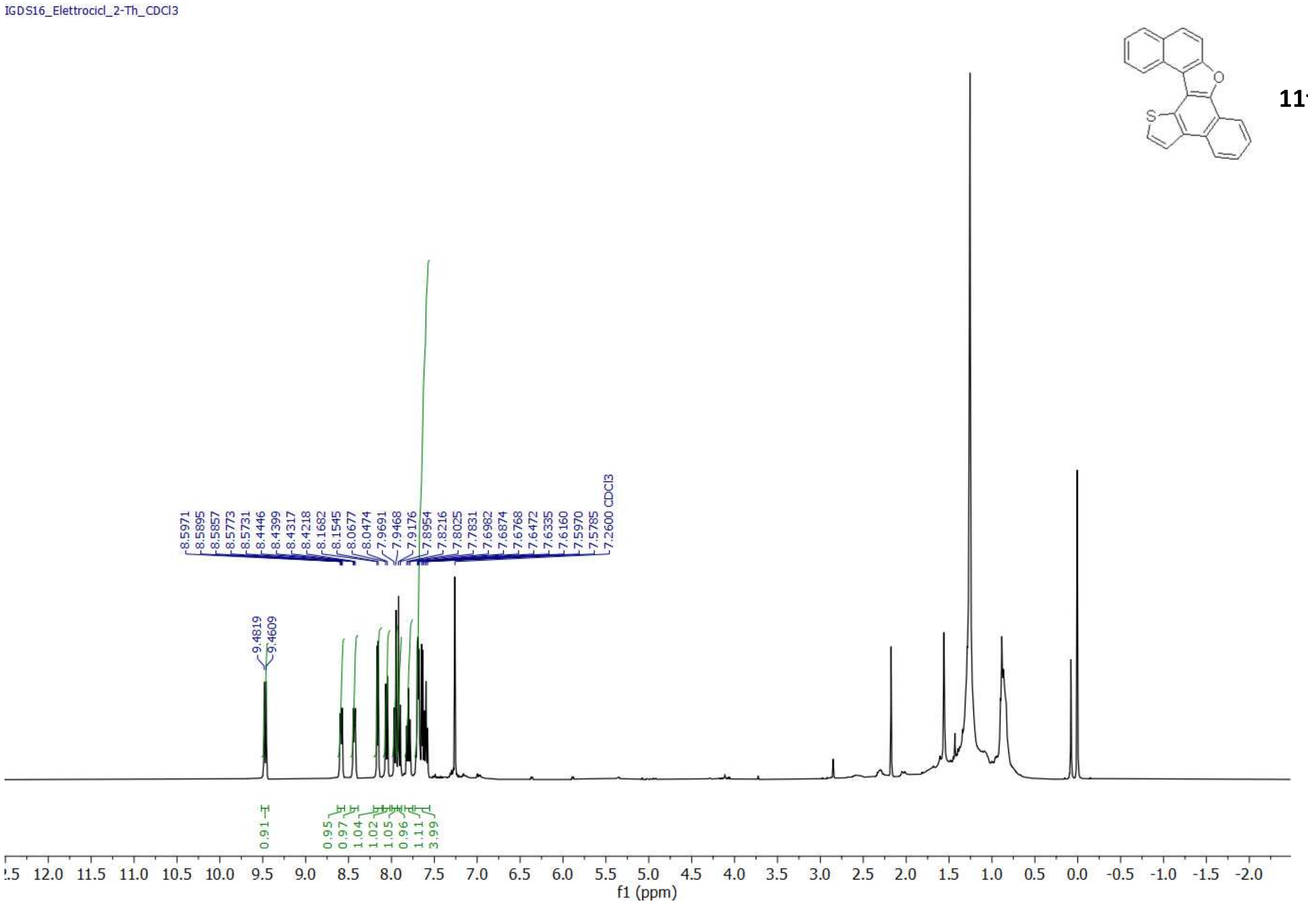
IGDS16_Elettrocicl_2-Th_CDCl3
11f
9.4819
9.4609
8.5971
8.5895
8.5857
8.5773
8.5731
8.4446
8.4399
8.4317
8.4218
8.1682
8.1545
8.0677
8.0474
7.9691
7.9468
7.9176
7.8954
7.8216
7.8025
7.7831
7.6982
7.6874
7.6768
7.6472
7.6335
7.6160
7.5970
7.5785
7.2600 CDCl3
0.91
0.95
0.97
1.04
1.02
1.05
0.96
1.11
3.99
f1 (ppm)

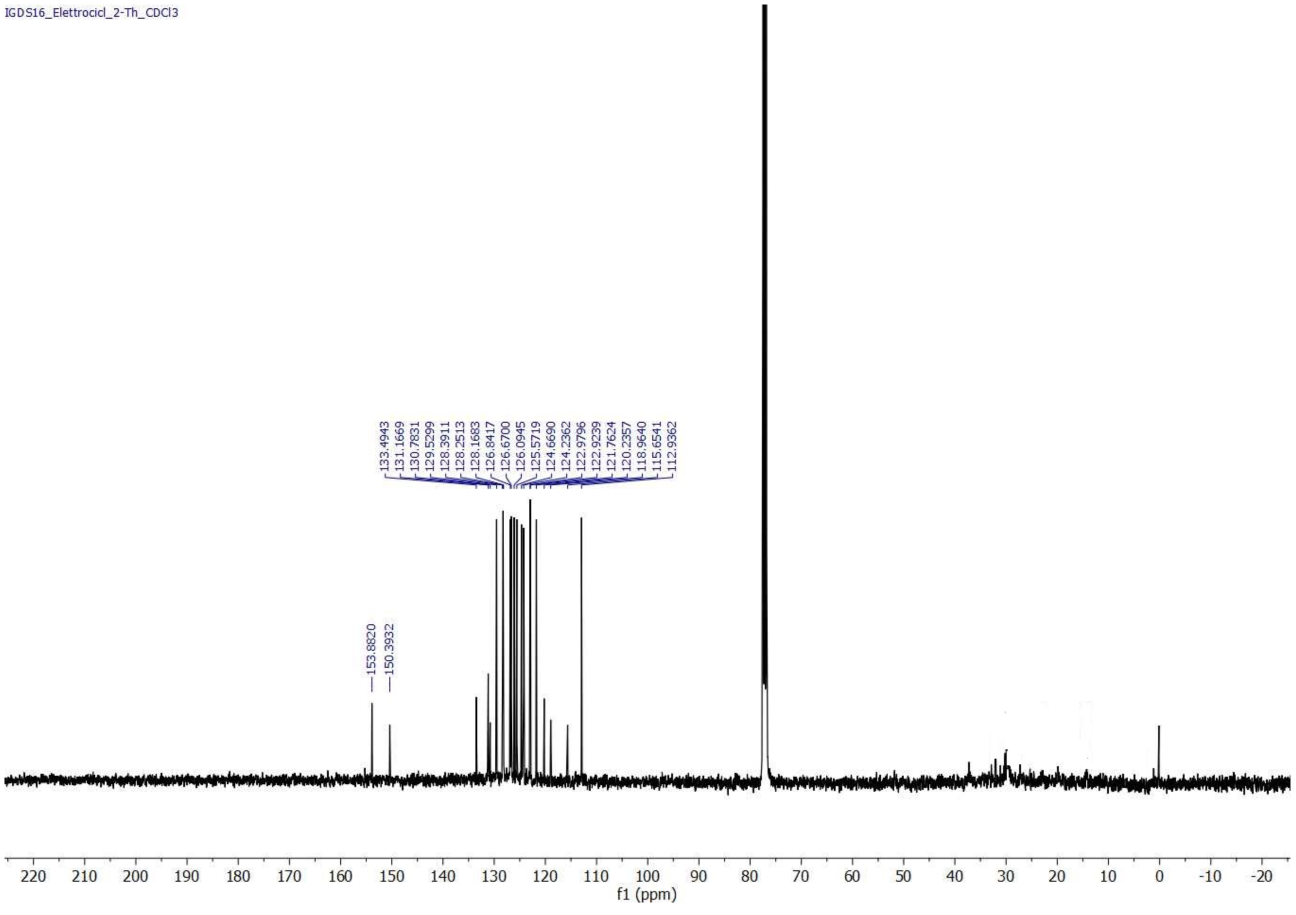
IGDS16_Elettrocicl_2-Th_CDCl3
153.8820
150.3932
133.4943
131.1669
130.7831
129.5299
128.3911
128.2513
128.1683
126.8417
126.6700
126.0945
125.5719
124.6690
124.2362
122.9796
122.9239
121.7624
120.2357
118.9640
115.6541
112.9362
f1 (ppm)

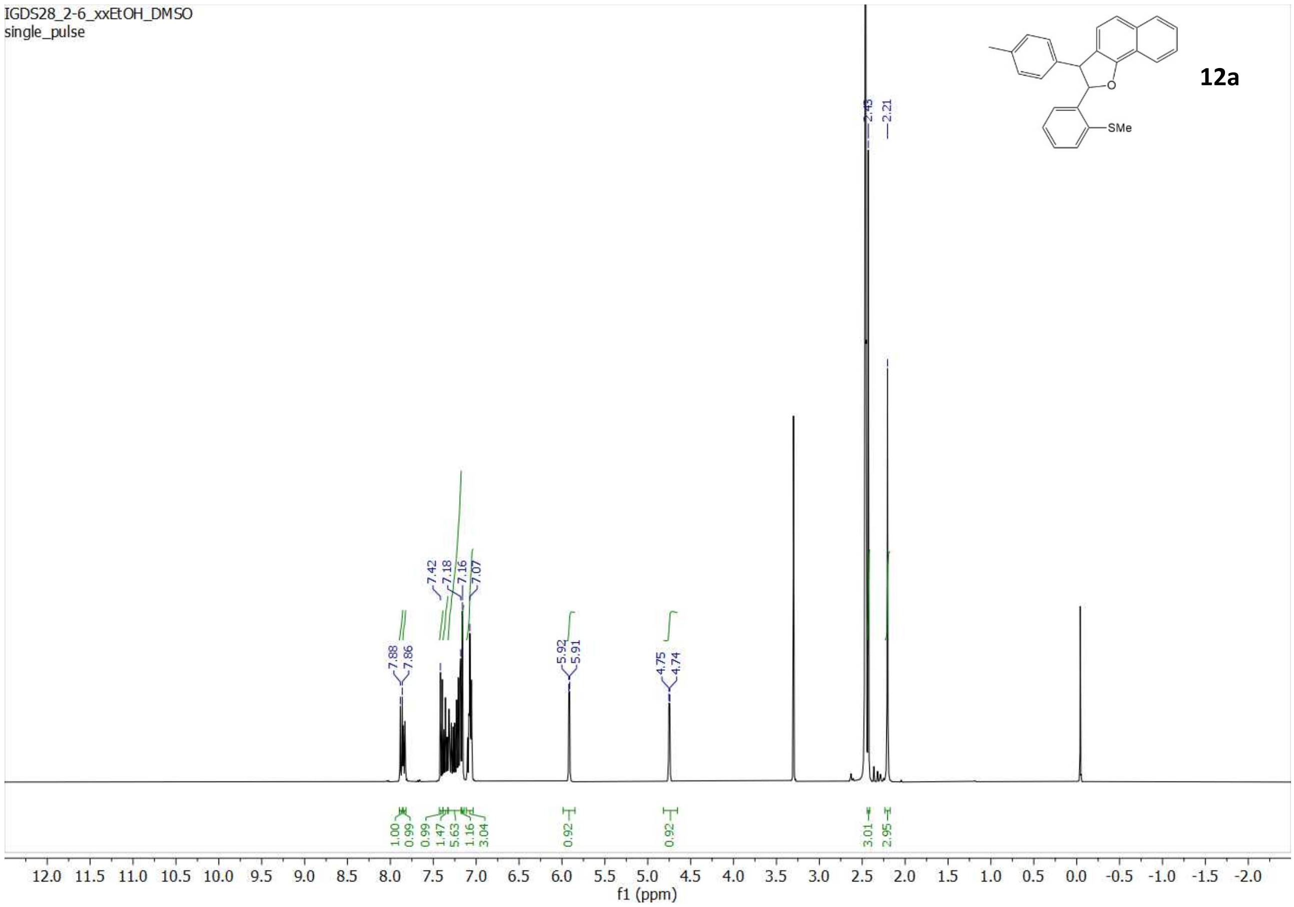
IGDS28_2-6_xxEtOH_DMSO
single_pulse
12a
SMe
7.88
7.86
7.42
7.18
7.16
7.07
5.92
5.91
4.75
4.74
2.43
2.21
1.00
0.99
0.99
1.47
5.63
1.16
3.04
0.92
0.92
3.01
2.95
12.0 11.5 11.0 10.5 10.0 9.5 9.0 8.5 8.0 7.5 7.0 6.5 6.0 5.5 5.0 4.5 4.0 3.5 3.0 2.5 2.0 1.5 1.0 0.5 0.0 -0.5 -1.0 -1.5 -2.0
f1 (ppm)

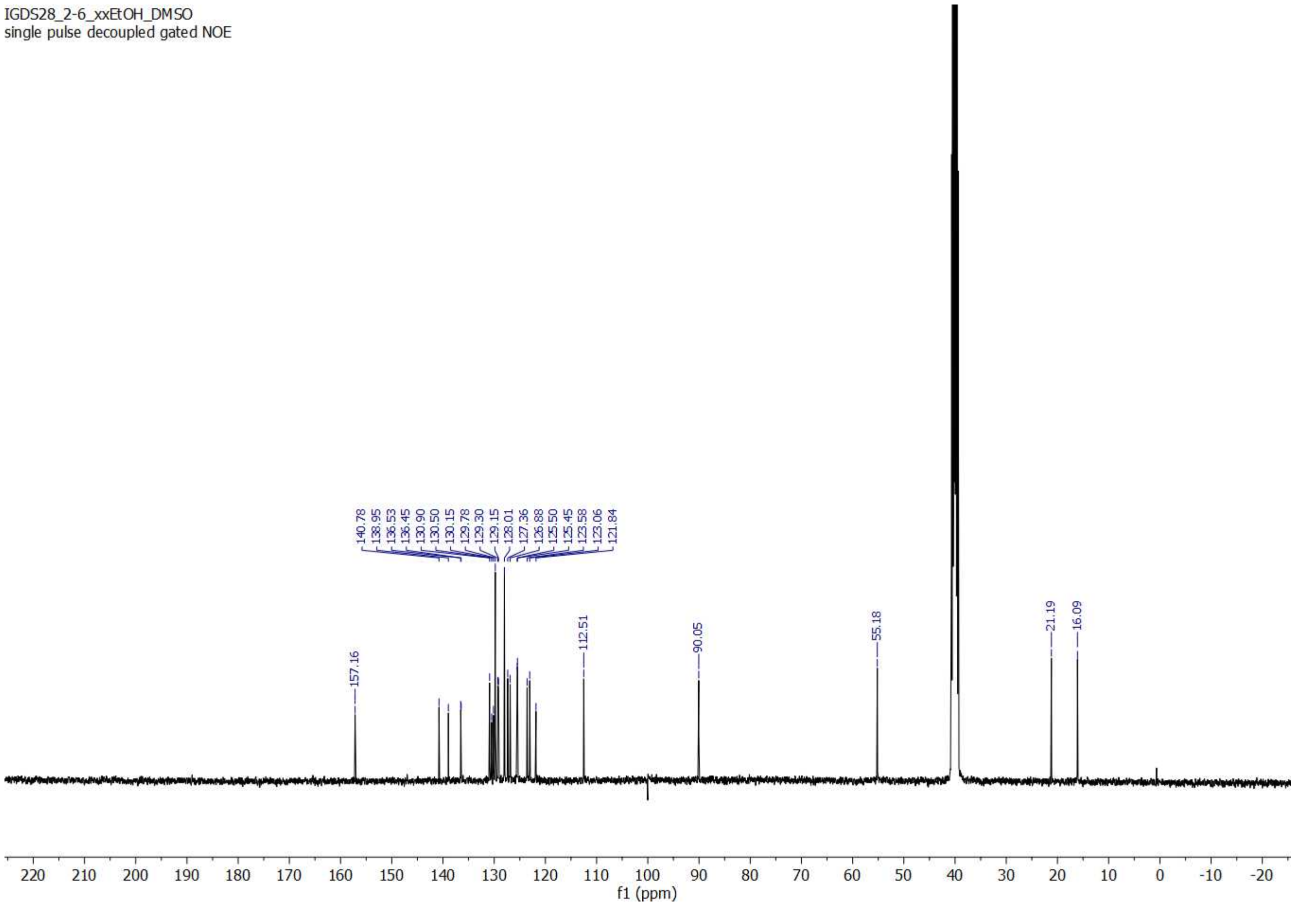
IGDS28_2-6_xxEtOH_DMSO
single pulse decoupled gated NOE
140.78
138.95
136.53
136.45
130.90
130.50
130.15
129.78
129.30
129.15
128.01
127.36
126.88
125.50
125.45
123.58
123.06
121.84
157.16
112.51
90.05
55.18
21.19
16.09
220 210 200 190 180 170 160 150 140 130 120 110 100 90 80 70 60 50 40 30 20 10 0 -10 -20
f1 (ppm)

IISAS4
single_pulse

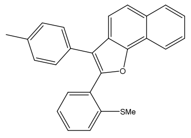

13a

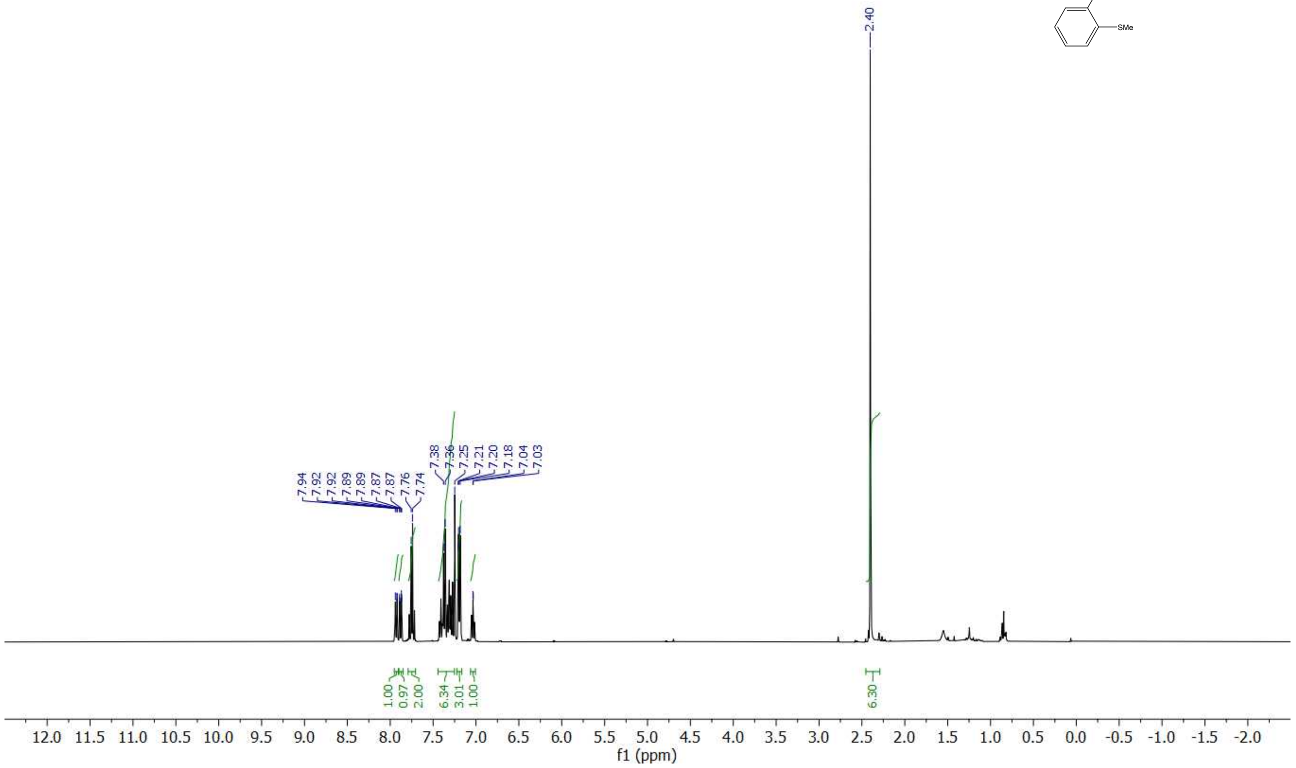

IISAS4

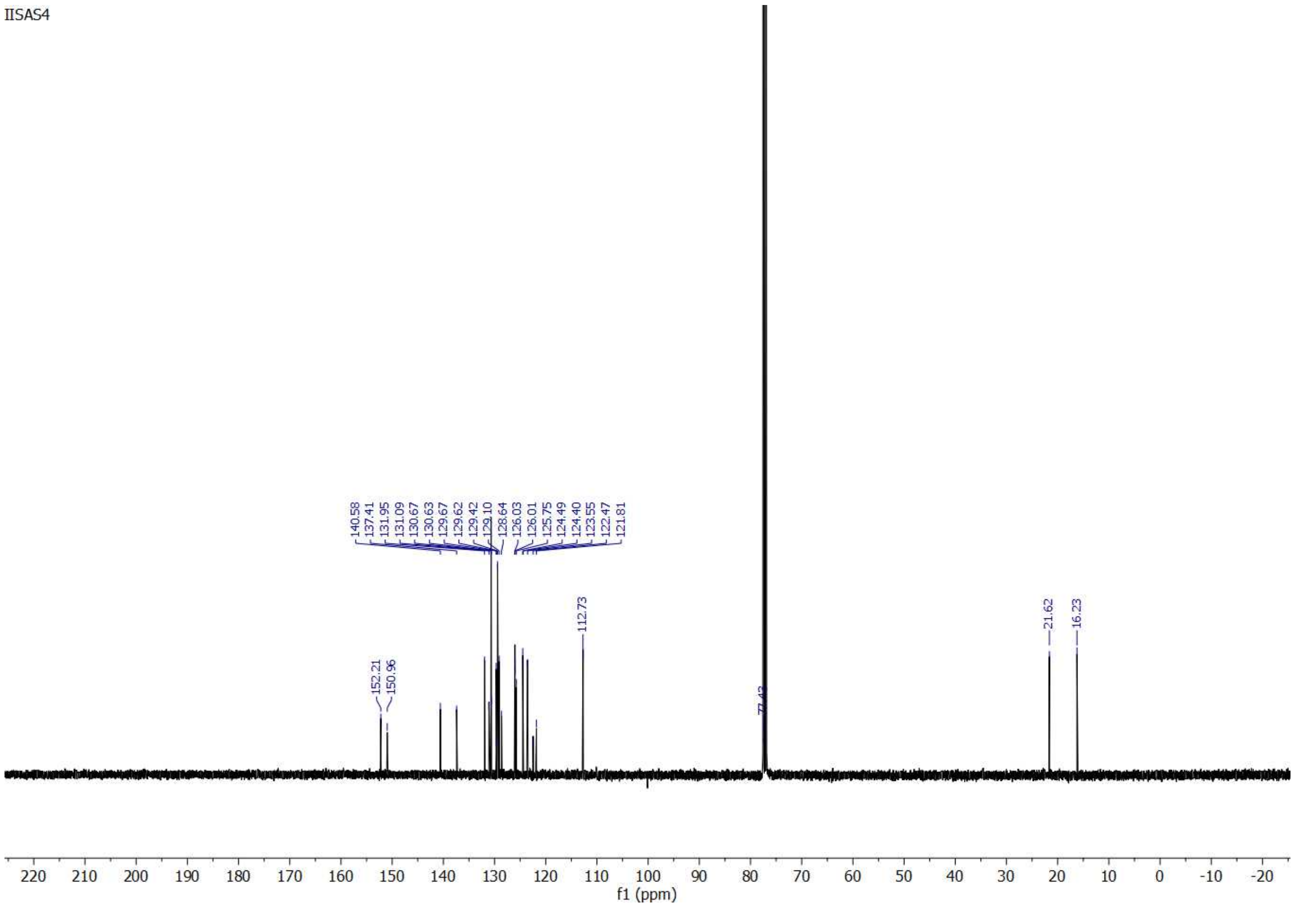

Supplement: Supplementary file 1 [file molecules-27-03147-s001.zip › molecules-1702192-SI.pdf]
